# Epigenetic Regulation of Aging and its Rejuvenation

**DOI:** 10.1002/mco2.70369

**Published:** 2025-09-01

**Authors:** Yongpan An, Qian Wang, Ke Gao, Chi Zhang, Yanan Ouyang, Ruixiao Li, Zhou Ma, Tong Wu, Lifan Zhou, Zhengwei Xie, Rui Zhang, Guojun Wu

**Affiliations:** ^1^ Department of Urology Xi'an People's Hospital (Xi'an Fourth Hospital) School of Life Sciences and Medicine Northwest University Xi'an China; ^2^ State Key Laboratory of Cancer Biology Department of Biochemistry and Molecular Biology Air Force Medical University Xi'an China; ^3^ Peking University International Cancer Institute Peking University Health Science Center Peking University Beijing China

**Keywords:** aging, epigenetic rejuvenation, epigenetic clocks, epigenetic mechanisms, emerging rejuvenation strategies

## Abstract

Aging increases the global burden of disease, yet its molecular basis remains incompletely understood. Recent studies indicate that reversible epigenetic drift—spanning DNA methylation clocks, histone codes, three‐dimensional chromatin, and noncoding RNA networks—constitutes a central regulator of organismal decline and age‐related diseases. How these epigenetic layers interact across different tissues—and how best to translate them into therapeutic strategies—are still open questions. This review outlines the specific mechanisms by which epigenetic changes influence aging, highlighting their impact on genomic instability, stem‐cell exhaustion, and mitochondrial dysfunction. We critically evaluate emerging rejuvenation strategies—partial OSKM reprogramming, CRISPR–dCas9 epigenome editing, NAD⁺/sirtuin boosters, HDAC inhibitors, microbiota transfer, and precision lifestyle interventions—detailing their efficacy in resetting epigenetic age and restoring tissue homeostasis. Integrating single‐cell multiomics and second‐generation epigenetic clocks, we propose a roadmap for translating these insights into safe, personalized antiaging medicine.

## Graphical abstract

This article provides an overview of the epigenetic mechanisms and key targets involved in aging, including DNA methylation, histone modifications, chromatin remodeling, and changes in noncoding RNAs. It also introduces various intervention strategies aimed at reversing epigenetic changes to delay aging and treat diseases.

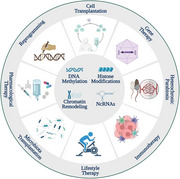

## 1 Introduction

Aging refers to the process by which the functions of various levels of the organism (cells, tissues, organs, etc.) gradually decline and structural changes occur over time. This is typically accompanied by reduced adaptability, weakened immune function, metabolic disorders, and an increased risk of age‐related diseases (such as cardiovascular diseases, cancer, neurodegenerative diseases, etc.), ultimately leading to death [1]. As medical technology has advanced, the global average lifespan has risen markedly. With the drop in the birth rate, populations are aging, and the 65‐plus‐age group is growing faster than others. The United Nations projects that by 2050, one‐sixth of the global population will be over 65 years of age, and those over 80 years will triple. Since aging is a major factor in chronic diseases such as cancer, it has replaced infectious diseases as the main cause of death and disability, imposing a heavy social burden. Thus, research on aging is highly valuable as it can achieve healthy longevity. Studies show that antiaging research may yield more economic benefits than tackling individual diseases [2].

At present, the widely accepted mechanisms of aging mainly include 14 types, such as genomic instability, telomere attrition, epigenetic alterations, loss of proteostasis, autophagy dysfunction, deregulated nutrient‐sensing, mitochondrial dysfunction, cellular senescence, and so on [3]. Meanwhile, research shows that compared with genetic information, the loss of epigenetic information plays a core role in the regulation of aging. This conclusion has been confirmed in various model organisms, including yeast, nematodes, fruit flies, and mice [4, 5]. Epigenetics refers to the discipline of heritable changes in gene function without changes in the DNA sequence, which eventually leads to phenotypic changes. As we age, epigenetic changes accumulate, leading to gradual alterations in gene expression and accelerating the aging process. The main mechanisms of these epigenetic changes in regulating longevity pathways include alterations in DNA methylation patterns, abnormal posttranslational modifications of histones, abnormal chromatin remodeling, and dysfunction of noncoding RNAs (ncRNAs). These regulatory and often reversible changes affect gene expression and other cellular processes, leading to the occurrence and progression of various age‐related human pathologies, such as cancer, neurodegenerative diseases, metabolic syndrome, and bone diseases [6].

An increasing number of studies suggest that lifestyle interventions, chemical drug treatments, gene therapy, stem cell transplantation, gut microbiota transfer, immunotherapy, heterochronic parabiosis, and reprogramming are epigenetic therapeutic strategies that can effectively improve or even reverse the aging state of the organism [7, 8]. Therefore, epigenetic regulation can be seen as a crucial entry point for further understanding the mechanisms underlying aging, exploring new aging biomarkers, and developing antiaging drugs and clinical treatment strategies in the future.

The objective of this review is to explore the role of epigenetic mechanisms in aging and their contribution to age‐related diseases. We will examine the key epigenetic processes—DNA methylation, histone modifications, and ncRNAs—and discuss how these mechanisms accumulate over time and influence aging. This review will also highlight recent advances in epigenetic rejuvenation therapies, such as CRISPR/Cas9‐based epigenetic editing, small molecule modulators, and the use of ncRNAs to restore youthful gene expression. These therapies offer promising strategies for extending healthspan and improving the quality of life for aging populations. Additionally, the review will address challenges and risks associated with epigenetic therapies, including off‐target effects and tumorigenesis, and propose future research directions to overcome these issues. By synthesizing recent research, this review aims to provide a comprehensive understanding of the role of epigenetics in aging and disease, and the potential of epigenetic interventions for rejuvenation.

## 2 Epigenetic Mechanisms of Aging

**FIGURE 1:**
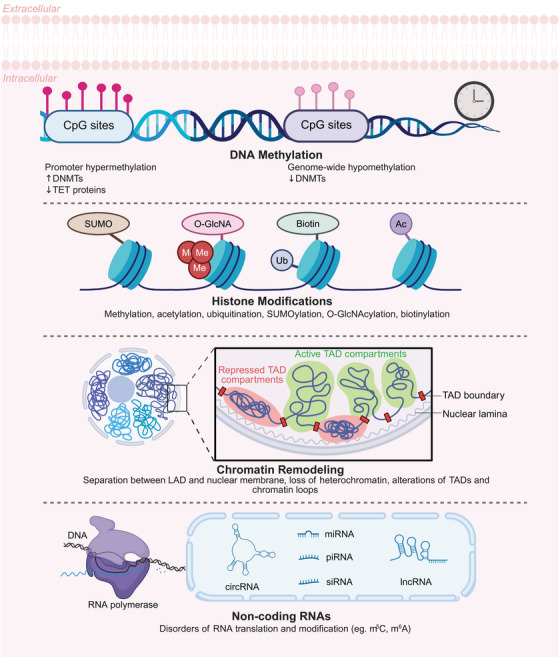
Epigenetic mechanisms underlying the aging process. This schematic diagram summarizes the major epigenetic mechanisms contributing to the aging process, including DNA methylation alterations, histone modifications, chromatin structural changes, and noncoding RNA dysregulation. The illustration shows how promoter hypermethylation silences critical genes while global hypomethylation occurs across the genome, mediated by DNMTs and TET proteins acting on CpG sites. Various histone modifications (methylation, acetylation, ubiquitination, SUMOylation, O‐GlcNAcylation, and biotinylation) dynamically regulate chromatin states. Age‐related chromatin remodeling involves disruption of TAD organization, with changes in active/repressed compartments, boundary integrity, and LAD, accompanied by heterochromatin loss. Additionally, noncoding RNAs (lncRNAs, circRNAs, siRNAs) and RNA modifications exhibit age‐associated dysregulation, affecting transcriptional and translational processes. Collectively, these interconnected epigenetic changes drive the progressive functional decline characteristic of cellular aging. This figure was created by BioRender. *Abbreviations*: DNMT, DNA methyltransferase; TET, ten‐eleven translocation; CpG, cytosine–phosphate–guanine dinucleotide; SUMO, SUMOylation; O‐GlcNA, O‐GlcNAcylation; TAD, topologically associating domain; LAD, lamina‐associated domain; m5C, 5‐methylcytosine; m6A, N⁶‐methyladenosine.

**FIGURE 2:**
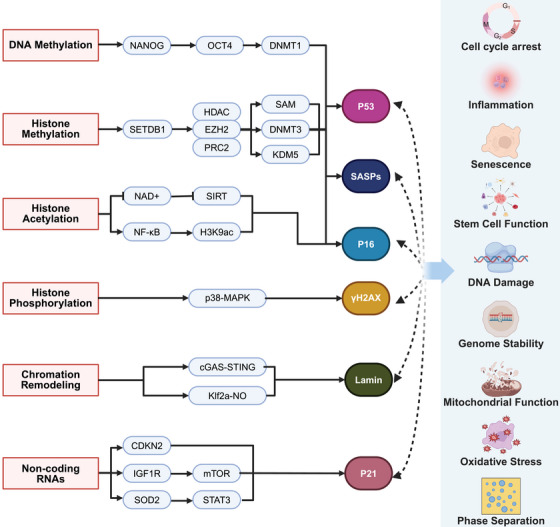
The schematic illustrates key epigenetic mechanisms and their downstream cellular effects. The left section outlines critical epigenetic mechanisms, including DNA methylation (mediated by DNMT1, DNMT3, and associated with pluripotency factors NANOG and OCT4), histone methylation (involving SETDB1, EZH2/PRC2, and KOM5 with H3K9ac and SAM as cofactors), histone acetylation/phosphorylation (featuring H3K9ac, HDAC, and p38–MAPK), and chromatin remodeling (linked to GAS–STING, mTOR, and STAT3 pathways). The right section demonstrates their downstream effects on cellular functions, such as cell cycle regulation and senescence (controlled by p53, p16, CDKN2, and p21), stress responses (including oxidative stress via SOD2, mitochondrial function through NAD+, and DNA damage repair), stem cell dynamics (influenced by NANOG/OCT4 and mTOR signaling), and phase separation in nuclear organization. Together, this integrated network reveals how epigenetic modifications converge with signaling pathways to govern genome stability, inflammation, senescence, and stem cell function. This figure was created by BioRender.

The epigenetic modification mechanisms associated with aging mainly include changes in DNA methylation patterns, abnormal posttranslational modifications of histones, abnormal chromatin remodeling, and dysfunction of ncRNA (Figure 1). This section will systematically elucidate the interplay between epigenetic dysregulation and aging, focusing on the latest discoveries and their implications for aging and disease, while also discussing potential targets and mechanisms that may delay or even reverse the aging process (Figure 2).

### 2.1 DNA Methylation Dynamics

DNA methylation is one of the most well‐known epigenetic modifications. DNA methylation is catalyzed by DNA methyltransferases (DNMTs) using S‐adenosyl‐methionine (SAM) as the methyl donor, which transfers a methyl group (–CH_3_) to specific bases in defined genomic regions. In mammals, the three principal methyltransferases are DNMT3A, DNMT3B, and DNMT1. According to the methylation pattern they establish, these enzymes are classified as either de novo or maintenance methyltransferases. DNMT3A and DNMT3B are de novo methyltransferases responsible for establishing methylation on previously unmethylated DNA. This de novo methylation—that is, the creation of new methylation marks on unmethylated cytosines—occurs predominantly during embryonic stem‐cell development and sets up novel methylation patterns. DNMT1 is a maintenance methyltransferase that preserves existing methylation patterns. After DNA replication, DNMT1, as an integral component of the replication complex, recognizes hemimethylated CpG sites on the nascent strand. Through a nucleophilic attack, it catalyzes methylation of these hemimethylated positions, thereby copying the methylation pattern from the parental strand to the daughter strand and ensuring faithful propagation of the methylation landscape originally established by the de novo methyltransferases [9, 10]. This modification is crucial for regulating gene expression, maintaining genomic stability, and facilitating cellular differentiation.

#### 2.1.1 Age‐Related Methylation Patterns

Age‐related changes in DNA methylation patterns are not only widespread but also have a significant impact on gene expression, influencing various life processes such as metabolism, inflammation, cancer, cardiovascular diseases, neurological disorders, and aging [11, 12, 13, 14, 15, 16]. Early studies found that in certain aging human and mouse cells, tissues, and organs (e.g., T cells, small intestine mucosa, liver, and brain), there is often a decrease in overall DNA methylation levels [9], while focal hypermethylation can emerge [17, 18] and correlate with a higher incidence of cardiovascular disease in older individuals [15]. A meta‐analysis from 128 mammal species revealed that hypermethylation of CpG islands is present in aging blood, brain, cortex, liver, muscle, and skin [19]. These patterns arise primarily from cell‐type‐specific, age‐stage‐specific, and stress‐signal‐specific transcriptional regulation of the three canonical DNMTs (DNMT1, DNMT3A, and DNMT3B). In replicative‐exhaustion cellular models, all three DNMTs are downregulated. By contrast, during in vivo aging driven by oxidative stress or chronic inflammation, DNMT1 is suppressed via the telomere–p53 axis, whereas DNMT3A/3B are upregulated through NF‐κB/STAT3 activation. The combined effect is the emergence of an “aging epigenetic signature” characterized by global hypomethylation superimposed on region‐specific hypermethylation [20, 21, 22]. Current research is increasingly focused on age‐related, region‐or site‐specific methylation changes—such as differentially methylated regions and differentially methylated positions—to dissect the molecular underpinnings of this epigenetic drift [23].

DNA methylation regulates aging by silencing or inducing aging‐related genes. During aging, the hypermethylation of specific gene promoters can silence crucial genes, particularly those involved in tumor suppression and immune response. For example, studies have shown that hypermethylation of the TP53 gene is associated with reduced expression in older populations [24, 25], which may contribute to the increased incidence of malignancies in the aging population. Furthermore, hypermethylation of inflammatory genes such as IL1β, IL6, and TNFα is linked to atherosclerosis [26], which poses significant health risks to older adults. Reduced DNA 5mC levels can upregulate PSG, endogenous retroviruses (ERVs), p15, p16, p21, and LINE‐1, while downregulating ELOVL2. Increased DNA 6mA can upregulate heat stress response genes. These changes can induce senescence‐associated secretory phenotype (SASP), inhibit the cell cycle, and accelerate cellular senescence [27, 28, 29, 30, 31, 32].

Another mechanism of DNA methylation in aging suggests that the widespread decay of the methylome reflects an “epigenetic maintenance system” that supports development, cell differentiation, and cell identity maintenance. Changes in methylation at specific genomic sites are crucial for preserving stem cell identity and function [9]. DNA methylation changes often reduce stem cell numbers and functions, such as impairing self‐renewal and causing differentiation bias, mirroring what is observed in aging [33]. Supporting this, single‐cell analysis in mice shows that liver cells’ epigenetic aging is precisely tracked, while muscle stem cells show minimal epigenetic age changes [34]. This indicates that the epigenetic clock responds when stem cells are stimulated to divide [35]. In summary, the mechanisms of how methylation changes accelerate aging are currently thought to be primarily through the silencing or induction of aging‐related genes and the impact on stem cell numbers and functions.

#### 2.1.2 Epigenetic Clocks

Epigenetic clocks are DNA methylation‐based biomarkers used to estimate the difference between an individual's biological age and chronological age. The core concept of epigenetic clocks is that over time, the DNA methylation patterns at certain specific locations in the genome undergo predictable changes, which are closely related to aging, health status, and lifespan prediction. Different epigenetic clock models rely on different algorithms and DNA methylation sites, and various types of age clocks have been developed with different machine algorithms, research species, and tissue sources [12, 36, 37, 38, 39].

The first‐generation DNA methylation clocks (DNAm) estimate biological age using specific CpG sites, with the most famous being the Horvath clock and the Hannum clock [40, 41]. Second‐generation clocks, such as PhenoAge, GrimAge, and DunedinPACE [42, 43, 44], incorporate clinical biomarkers alongside DNA methylation data, improving accuracy and applicability. As a result, these clocks are not only associated with physiological age but also predict the onset of aging‐related diseases, facilitate disease diagnosis, and contribute to health assessments [12, 36, 37, 39, 45].

With the emergence of various machine learning and deep learning models, the algorithms for DNAm are continuously being updated. For example, the DNAge model, based on Horvath's pan‐tissue clock, assesses the actual age of skeletal muscle in aging mice through exercise [46]. Recent research has focused more on lifestyle and environmental factors, such as alcohol consumption and sleep quality [47], and their impact on epigenetic age acceleration (EAA) assessments. For example, the newly developed GrimAge V2, using deep learning methods, shows better predictive performance than its V1 version [48].

The clinical application value of epigenetic clocks is becoming increasingly prominent. Studies have shown that EAA assessed by epigenetic clocks is associated with an increased risk of aging‐related diseases, such as Alzheimer's disease (AD) and cardiovascular diseases [45, 49, 50]. One study utilized DNA methylation data from 378 women to develop the first second‐generation epigenetic age clock for skin, which can accurately predict skin aging phenotypes represented by wrinkle grade, visual facial age, and visual age progression [51].

Furthermore, these clocks have primarily been developed for European or Hispanic populations. A study using DNA methylation data from blood samples of Koreans demonstrated the applicability of epigenetic clocks in East Asian populations. This study not only included chronic disease factors, blood biomarker levels, and lung function but also considered health behavior factors, socioeconomic status, and psychological stress levels [52], confirming the association between EAA and environmental factors in Asian populations.

### 2.2 Histone Modification Alterations

Histones are small, basic proteins rich in amino acids such as lysine and arginine, and they are the major structural proteins of eukaryotic chromatin [53]. They bind to DNA to form nucleosomes, which are the fundamental repeating units of chromatin. There are four core histones: H2A, H2B, H3, and H4, each composed of two molecules to form an octamer, around which DNA is wound to form the nucleosome [54]. H1 is the linker histone [55], located at the linker DNA regions between nucleosomes, helping to compact higher‐order chromatin structures. The amino acid sequences of histones are highly conserved through evolution, especially H3 and H4, indicating the importance of their functions [56].

Histone modification refers to the process by which specific amino acid residues (especially at the N‐terminal tails) on histones undergo covalent chemical modifications. By adding or removing small chemical groups (such as methyl, acetyl, or phosphate groups), histone modifications dynamically regulate chromatin structure and gene expression [22, 57]. These modifications do not alter the DNA sequence but are heritable and influence cellular functions, making them one of the core mechanisms of epigenetic regulation. This process is dynamic and reversible, with “writers” (such as histone acetyltransferases [HATs] and HMTs) adding modifications and “erasers” (such as histone deacetylases [HDACs] and KDMs) removing them [58, 59]. Histone modifications include various types, with common ones being methylation, acetylation, phosphorylation, and ubiquitination. On one hand, these posttranslational modifications can activate or repress gene expression by regulating chromatin structure. On the other hand, different combinations of modifications can form a “histone code” [60, 61], which is recognized by effector proteins (such as proteins containing Bromo/Chromo domains) and recruits the transcriptional machinery to initiate distinct gene expression programs. Histone modifications play multiple biological roles in eukaryotic cells, participating in the regulation of chromatin structure, gene expression, DNA damage and repair, and the cell cycle [60, 61]. These functions are realized through complex interaction networks, and different modifications may have synergistic or antagonistic effects [62, 63]. Abnormal histone modifications are closely associated with various diseases, such as tumors [64], neurodegenerative diseases [65], and aging [66, 67, 68], and are potential therapeutic targets.

#### 2.2.1 Histone Methylation

Recent studies have shown that histone methylation marks such as H3K4me3, H3K27me3, and H3K36me3 undergo significant changes during aging, and these changes directly affect the aging and repair capacity of cells [69]. H3K4me3 is closely related to the expression of aging‐associated genes. Research has found that in the hematopoietic stem cells (HSCs) of aging mice, the level of H3K4me3 is increased [70], while in physiologically aged human HSCs, H3K4me1, H3K4me3, and H3K27ac levels are reduced [71]. Histone methylation exhibits significant spatiotemporal‐specific changes during aging, and these modifications directly impact chromatin structure and gene expression patterns through epigenetic regulatory networks, becoming a key molecular basis for the development of aging and related diseases [72, 73, 74, 75]. Recent studies have shown that aging cells display typical characteristics of methylation reprogramming: (1) Repressive marks, such as H3K27me3 (trimethylation of lysine 27 on histone H3), undergo specific loss at gene promoter regions, especially at cell cycle inhibitor genes (such as p16INK4a/CDKN2A) [76, 77, 78, 79]. Similar to the mechanism of DNA hypomethylation in promoting aging, histone hypo‐methylation, such as decreased H3K9me3, H4K20me3, H3K9, and H3K36 methylation, can upregulate PSG, p15, p16, p21, and LINE‐1, while downregulating ELOVL2 [27, 28, 29, 30, 31, 32]. These changes induce cell cycle arrest and activate SASP. (2) The reduction of H3K9me3 in heterochromatic regions leads to decreased genomic stability, abnormal activation of transposons, and nuclear structure disruption [31, 80, 81]. (3) The abnormal accumulation of the active mark H3K4me3 at the promoter regions of metabolism‐related genes [28, 57, 82], which reprograms cellular. These changes are closely associated with the dysregulation of methylation‐modifying enzymes, including the reduced activity of the Polycomb repressive complex 2 (PRC2) core enzyme EZH2 [83, 84], the upregulation of KDM demethylase enzymes (such as KDM5A/B) [63, 69, 85], and the age‐dependent reduction of the methyl donor SAM [86, 87, 88, 89, 90, 91]. These changes induce DNA damage and oxidative stress, activate mTOR to regulate metabolism, and promote aging.

In aging‐related diseases, these methylation abnormalities exhibit tissue‐specific patterns. In neurodegenerative diseases (such as AD), neurons in the prefrontal cortex show a significant increase in H3K9me2 [92] and a decrease in H3K27me3 [93], leading to the silencing of synaptic plasticity genes [94]; in muscle atrophy, aging mice muscles show an elevated level of H3K27me3 [95, 96], which inhibits the differentiation of muscle stem cells and muscle regeneration. The expression of the antiaging protein Klotho can suppress the activity of the H3K27 demethylase KDM6B/JMJD3, reduce H3K27me3 levels, and promote muscle stem cell differentiation [97]. Additionally, H3K36me3 levels significantly decrease, which may be related to a decline in DNA repair capacity [98] and impaired muscle stem cell function [99]; in cardiovascular aging, the loss of H3K27me3 in endothelial cells promotes the expression of inflammatory factors, accelerating the progression of atherosclerosis [100, 101, 102, 103]; in aging‐related cancers, the abnormal distribution of H3K36me3 leads to DNA damage repair defects and increased genomic instability [104, 105, 106]. Notably, these epigenetic changes form a positive feedback loop with classic aging markers (such as mitochondrial dysfunction and stem cell depletion): increased mitochondrial reactive oxygen species (ROS) production can suppress the activity of HMTs [107, 108], while abnormal methylation patterns further affect nuclear–mitochondrial communication [109].

Currently, therapeutic strategies targeting these methylation regulatory mechanisms (such as EZH2 inhibitors, KDM inhibitors, and methyl donor supplementation) have shown potential for improvement in various aging‐related disease models [109, 110], providing important directions for the development of new antiaging interventions.

#### 2.2.2 Histone Acetylation

Recent studies have revealed the bidirectional regulatory characteristics of histone acetylation in the aging process: on one hand, there is a progressive decline in global acetylation levels, while on the other hand, specific functional genomic regions (such as inflammation‐related gene loci) show abnormal acetylation accumulation. This “global loss‐local gain” pattern constitutes an epigenetic hallmark of aging [3, 97, 111]. At the molecular level, aging‐related histone acetylation disorders mainly involve three core aspects: imbalance in the acetylation “writing–erasing” system, disruption of the metabolic–epigenetic network, and changes in chromatin spatial organization [112, 113, 114].

The dynamic equilibrium system composed of HATs and HDACs undergoes significant changes during aging [115]. Studies have shown that the expression and catalytic activity of major HATs such as p300/CBP decrease with age, and their nuclear localization also becomes abnormal [116, 117]. This functional decline of HATs leads to a reduction in acetylation levels at several key sites, including H3K14 and H4K16 [118, 119]. Meanwhile, the expression and activity of NAD^+^ dependent class III HDACs (sirtuin family) show tissue‐specific variations, SIRTs expression is downregulated in most tissues [120, 121, 122, 123]. Notably, the functional decline of SIRT6 in the aging process is the most significant, leading to the abnormal accumulation of its target sites H3K9ac and H3K56ac [24, 124, 125], which in turn affects DNA damage repair and genomic stability.

Histone acetylation is closely linked to cellular metabolic states, and this feature is particularly prominent in the aging process. The decline in mitochondrial function results in reduced acetyl‐CoA production, which is the primary cause of the global decline in acetylation [126, 127]. On the other hand, age‐dependent reductions in NAD^+^ levels weaken the activity of SIRT1/6, forming a “metabolic–epigenetic vicious cycle”: decreased NAD^+^ → reduced SIRT activity → high histone acetylation → proinflammatory gene expression → mitochondrial damage → further depletion of NAD^+^ [120, 128, 129]. Additionally, aging‐related changes in α‐ketoglutarate levels indirectly regulate acetylation patterns by affecting TET enzyme activity [130].

The changes in histone acetylation during aging significantly impact the higher‐order structure of chromatin. Topology‐associated domains (TADs) boundary integrity of euchromatic regions (marked by H3K27ac) is disrupted in aged cells, leading to abnormal interactions of previously isolated functional genomic regions [131]. This structural change is closely related to the rearrangement of lamina‐associated domains (LADs), manifesting as the abnormal accumulation of H4K16ac in perinuclear heterochromatin regions [132, 133]. Notably, an “acetylation phase separation abnormality” phenomenon is observed in aging cells: certain transcription factors (such as NF‐κB) form biomolecular condensates by recognizing specific acetylation marks (such as H3K9ac), which exacerbates chronic inflammation [134].

Abnormal histone acetylation is closely associated with the development of various aging‐related diseases. In neurodegenerative diseases, hippocampal neurons show specific loss of H4K12ac, leading to the silencing of synaptic plasticity‐related genes (such as BDNF, Arc) [135]. Single‐cell epigenomic analysis reveals that the abnormal distribution of H3K27ac in neurons of AD patients precedes amyloid plaque formation [136, 137], suggesting that it may serve as an early diagnostic marker. In the cardiovascular system, the abnormal increase in H3K9ac in endothelial cells promotes atherosclerotic plaque formation by activating the NF‐κB signaling pathway [138, 139]. In metabolic diseases, the reprogramming of H3K27ac in liver tissue directly leads to the abnormal expression of key gluconeogenesis enzymes (PEPCK, G6Pase), exacerbating age‐related glucose metabolism disorders [140].

Intervention strategies targeting histone acetylation regulation in aging primarily focus on three directions: metabolic reprogramming (such as NAD^+^ precursor supplementation) [141, 142, 143], epigenetic editing (such as HATs/HDACs targeting regulation) [144], and anti‐inflammatory treatment (such as SASP inhibitors) [145]. Preclinical studies have shown that nicotinamide mononucleotide (NMN) can restore SIRT1 activity by elevating NAD^+^ levels [146]. Selective HDAC inhibitors (HDACis) have shown protective effects in neurodegenerative disease models [147]. Future research needs to address challenges such as tissue‐specific delivery and long‐term safety, and explore precision intervention strategies based on single‐cell epigenomics.

#### 2.2.3 Histone Phosphorylation

Recent studies have found that histone phosphorylation in aging cells exhibits significant reprogramming characteristics, manifested as abnormal accumulation of DNA damage‐related phosphorylation marks and reduced efficiency of signal‐responsive phosphorylation events. At the molecular level, aging‐associated histone phosphorylation disorders mainly involve three core aspects: (1) the functional decline of the DNA damage response (DDR) system leading to the persistent deposition of γ‐H2AX (H2AXS139ph); (2) changes in the activity of signaling pathways such as mitogen‐activated protein kinases (MAPK) and Aurora kinases, causing a remodeling of phosphorylation patterns; (3) disruption of the phosphatase network, which impedes dephosphorylation processes [65, 148, 149, 150, 151].

In young cells, DNA double‐strand breaks (DSBs) rapidly induce phosphorylation at the C‐terminal S139 site of H2AX (forming γ‐H2AX), which serves as a damage marker to recruit repair factors [148, 152]. However, aging cells exhibit two characteristic changes: one is a significant increase in baseline γ‐H2AX levels, indicating increased genomic instability [153]; the other is a decreased ability to form new γ‐H2AX foci upon damage, reflecting reduced repair efficiency [149]. This “high background‐low response” pattern is closely related to decreased ataxia‐telangiectasia mutated (ATM)/ataxia telangiectasia and Rad3‐related protein (ATR) kinase activity and overexpression of PP2A phosphatase [154]. Notably, the abnormal accumulation of γ‐H2AX in aging cells not only marks DNA damage but may also inhibit transcription factor access through spatial hindrance mechanisms, leading to the silencing of important metabolic genes (such as SIRT6) [155].The MAPK and Aurora kinase pathways are key hubs regulating aging‐related phosphorylation. p38–MAPK remains persistently activated in aging cells, which drives the production of the SASP by promoting NF‐κB recruitment [106]. In contrast, the activity of Aurora B kinase declines with age, causing chromosome segregation errors and karyotype instability [156].

### 2.3 Chromatin Remodeling and Architecture Shifts

Recent studies have shown that aging is accompanied by significant chromatin structural remodeling, including the loss of heterochromatin, disintegration of the 3D genome, and activation of transposons, which are epigenetic changes [157, 158, 159, 160]. The downregulation of nuclear lamina protein Lamin B1 in aging cells leads to nuclear membrane wrinkling and the redistribution of LADs, with LADs dissociating in aged tissues, resulting in the abnormal expression of previously silenced genes [161]. This change is closely associated with the SASP, LAD dissociation releases ERVs, driving chronic inflammation by activating the cGAS–STING pathway [162].

At the level of heterochromatin, several studies have reported aging‐related histone modification abnormalities. SUV39H1 catalyzes the trimethylation of histone H3 at lysine 9 (H3K9me3), which is specifically recognized and bound by the chromodomain of HP1 proteins. HP1 then homodimerizes and recruits additional SUV39H1, driving the formation of liquid‐like heterochromatin droplets and leading to heterochromatin relaxation [163, 164, 165, 166]. During stem cell aging, SUV39H1‐mediated H3K9me3 significantly decreased, leading to the derepression of satellite DNA repeats [167, 168, 169]. In 2023, research indicated that loss of H3K27me3 reactivates LINE‐1 transposons, promoting genomic instability [170]. 3D genome analysis revealed that in aging cells, the transition between A/B compartments increases, TAD boundary integrity weakens (especially at tumor suppressor gene loci such as p53), and enhancer–promoter interactions decrease, all of which collectively lead to disrupted gene expression networks [171, 172].

At the molecular level, chromatin remodeling involves abnormalities in multiple layers of regulation. Dysregulation of histone‐modifying enzymes is manifested by decreased HDAC activity, causing an increase in H4K16ac [173], decreased DNMT expression leading to global hypomethylation [174, 175], and NSD2 upregulation drives the aberrant accumulation of H3K36me2 [176]. This increase renders chromatin more open, facilitating the binding of transcription factors and other proteins to DNA and thereby enhancing gene transcription. Concurrently, the elevated H3K36me2 level mediated by NSD2 antagonizes EZH2‐catalyzed H3K27me3 deposition. Because H3K27me3 is a repressive mark, its reduction weakens chromatin‐mediated repression [177, 178, 179]. The function of chromatin remodeling complexes is also significantly impaired, SWI/SNF engages nucleosomes, transiently disrupts their DNA contacts, and generates short DNA loops that allow the nucleosome to slide to new positions, thereby promoting either transcriptional activation or repression. Concurrently, SWI/SNF forms a dynamic balance with Polycomb complexes (PRC1 and PRC2) during developmental gene regulation. SWI/SNF opens chromatin by dismantling PRC2‐mediated H3K27me3 domains, whereas PRC1 recondenses heterochromatin through deposition of H2AK119ub1. Disruption of this equilibrium is frequently associated with disease [180, 181, 182, 183], These changes drive Brg1 (SMARCA4) to bind the promoter of Klf2a, activating the Klf2a–NO pathway and thereby disrupting stem cell function [184]. Deficiency in the NuRD complex reduces DNA repair efficiency [185, 186, 187]. The regulatory role of ncRNAs is increasingly recognized, with the accumulation of aging‐related long ncRNAs (lncRNAs) like TERRA causing telomere heterochromatin disintegration [188, 189], and the circRNA affecting chromatin accessibility by influencing HMGB1 [190].

These chromatin changes are closely linked to various aging‐related diseases. In neurodegenerative diseases, AD patients’ neurons show an abnormal increase in H3K27ac, promoting tau overexpression [191, 192], while Parkinson's disease (PD) is associated with increased chromatin accessibility at the SNCA gene locus [193, 194]. Research in cancer shows that aging‐related disruption of TAD boundaries in leukemia activates proto‐oncogenes [195]. Metabolic diseases like diabetes are related to a decrease in enhancer–promoter interactions at the PDX1 locus in pancreatic β‐cells [196], while atherosclerosis involves inflammation triggered by the loss of H3K9me3 in endothelial cells [197].

In response to these findings, researchers have developed various intervention strategies. In epigenetic reprogramming, OSKM induction can partially restore chromatin structure in aging cells [198, 199], and HDACis can improve cognitive function in aging mice [200]. Attempts to target chromatin remodeling include inhibiting NSD2 to delay stem cell aging [201] and activating SIRT6 to enhance heterochromatin stability [202, 203]. In gene therapy, CRISPR–dead Cas9 (dCas9)‐mediated epigenetic editing has achieved precise regulation of disease‐associated loci [204, 205]. Despite the progress, the field still faces challenges such as unclear tissue‐specific regulations and the limitations of 3D genome dynamic monitoring technologies. Future work will need to develop single‐cell multiomics technologies and strengthen clinical translation research.

### 2.4 ncRNA Networks

ncRNAs are critical regulators of gene expression and play significant roles in the aging process. This diverse class of RNA molecules, including microRNAs (miRNAs), lncRNAs, and circular RNAs, contributes to various cellular functions and the pathogenesis of age‐related diseases [206].

miRNAs are small RNA molecules that posttranscriptionally regulate gene expression by targeting complementary mRNA sequences for degradation or translational repression. Recent studies have highlighted the importance of specific miRNAs in aging, for example. The most applicable age predictor miRNAs include miR‐9, miR‐21, miR‐34a, miR‐96, miR‐132, miR‐212, and miR‐145 [207]. Moreover, the dysregulation of specific miRNAs has been linked to the pathogenesis of age‐related diseases, including neurodegenerative disorders and cardiovascular diseases [208, 209]. The dysregulation of miRNAs, such as the downregulation of miR‐29, has been implicated in age‐related diseases, suggesting their potential as biomarkers and therapeutic targets [210].

The proven mechanism of miRNAs in aging regulation mainly involves targeting and binding to the mRNA sequences of aging‐related genes to induce degradation or inhibit translation. Here are some specific ways: (1) Affecting cell aging by regulating the p53 and p21 signaling pathways. For instance, the downregulation of miR‐15, 17, 19b, 20a, 302b, 106a, and b can boost p21 expression and promote aging. miR‐34a and miR‐217 can accelerate aging by targeting and suppressing SIRT1, which in turn increases p53 expression [211, 212]. (2) Influencing aging via oxidative stress regulation. For example, miR‐34a and miR‐335 are upregulated in aging cells. MiR‐34a targets mitochondrial antioxidant enzyme TXNRD2, and miR‐335 targets SOD2. These actions raise ROS levels and speed up cell aging [213]. (3) Impacting aging by regulating inflammation. miR‐146a and b can target and suppress the expression of IL6 and IL8, thereby slowing down aging [214].

Studies have shown that lncRNAs such as ANRIL, MALAT1, and H19 are abnormally expressed during aging, accelerating aging by affecting telomere maintenance, DNA damage repair, and cell cycle regulation [215, 216, 217, 218]. For example, ANRIL regulates the CDKN2A/B loci by binding to the PRC2, influencing cell senescence [219, 220]. H19 promotes the IGF1R/mTOR signaling pathway by inhibiting let‐7 miRNA activity, exacerbating aging‐related metabolic dysfunction [221, 222].

CircRNAs form through back‐splicing of precursor mRNA, resulting in a covalently closed circular structure with no 5′ cap or 3′ poly‐A tail, making them highly stable in cells [190]. Studies have found that circRNAs increase linearly during aging, suggesting their accumulation is primarily due to their high stability rather than increased synthesis rates [223, 224]. CircRNAs such as circHIPK3, circCDKN2B, and circFoxo3 regulate aging by adsorbing miRNAs or binding to RNA‐binding proteins [225, 226, 227, 228, 229]. CircHIPK3 acts as a sponge for miR‐124‐3p, upregulating the STAT3 signaling pathway and promoting cellular senescence [230]. CircCDKN2B inhibits cell cycle progression by binding to p21 protein [231]. CircFoxo3 exacerbates aging‐related muscle atrophy by stabilizing p21 and p27 mRNA [232].

Recent research has focused on elucidating the specific roles of ncRNAs in aging. For instance, manipulating levels of specific miRNAs has been shown to influence lifespan in model organisms, suggesting that miRNA‐based therapies could be developed for age‐related diseases [211, 233]. Additionally, lncRNAs that modulate cellular responses to stress and inflammation have gained attention for their potential implications in aging [234]. Findings indicate that specific circular RNAs can regulate SASP factors, further linking ncRNAs to aging and inflammation [235]. This highlights the intricate role of ncRNAs in the aging process and suggests that they may serve as potential therapeutic targets for age‐related interventions.

As research progresses, the development of therapies that target specific ncRNAs may provide innovative strategies for mitigating age‐related decline and promoting healthy longevity. For instance, the use of small RNA molecules to modulate miRNA expression levels could help restore normal cellular function in aging tissues, offering promising avenues for future therapeutic interventions.

### 2.5 Epigenetic Inheritance and Transgenerational Effects

**FIGURE 3:**
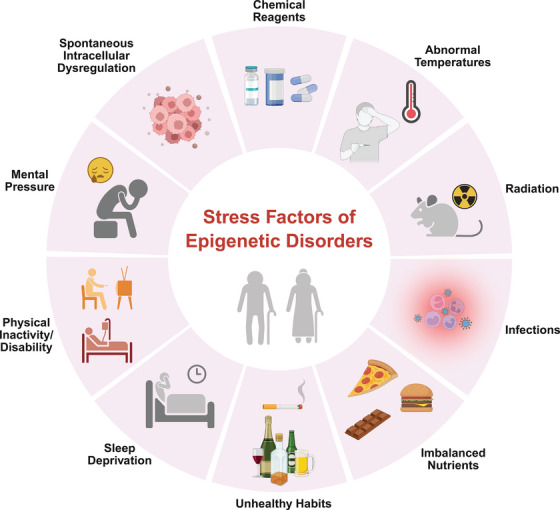
Stress factors leading to epigenetic disorders. The epigenetics of aging is influenced by various environmental and lifestyle factors, which can be categorized into several types: chemical reagents, radiation, abnormal temperatures, infections, imbalanced nutrition, unhealthy habits, sleep deprivation, physical inactivity or disability, mental pressure, spontaneous intracellular dysregulation, and so on. Each of these stress factors can impact gene expression and lead to various health issues, accelerating aging and highlighting the complex interactions between environmental stressors and gene regulation. This figure was created by BioRender.

In addition to epigenetic changes in individuals caused by various factors (Figure 3), specific epigenetic modifications can be inherited, leading to phenotypic changes in offspring, which is known as the inheritance and transgenerational effects of epigenetics. For instance, exposure to environmental stressors, such as toxins or dietary changes, can induce epigenetic alterations that persist across multiple generations [236, 237, 238, 239]. Parental exposure to a high‐fat diet can alter the DNA methylation patterns of offspring, making them more susceptible to metabolic disorders [240, 241]. Moreover, epigenetic changes can affect not only the phenotype of individuals but also their reproductive fitness, potentially leading to evolutionary implications. For example, if adverse environmental conditions trigger heritable epigenetic changes that confer a survival advantage, these modifications may become prevalent in the population over time [242, 243]. These findings emphasize the importance of understanding how parental environmental exposures affect offspring's epigenetic landscape. They may explain the intergenerational transmission of age‐related diseases and raise important questions about how lifestyle choices impact the long‐term health of future generations.

## 3 Functional Consequences of Epigenetic Dysregulation in Aging

Epigenetic dysregulation in aging manifests through progressive alterations in DNA methylation patterns, histone modifications, and chromatin remodeling, leading to global transcriptional instability and loss of cellular identity. Age‐related hypermethylation at promoter regions of tumor suppressor genes and hypomethylation at repetitive genomic elements contribute to genomic instability [244], while aberrant histone acetylation and methylation disrupt the expression of genes critical for stem cell maintenance, stress response, and metabolic homeostasis [245]. These changes drive cellular senescence by silencing proliferative genes and activating inflammatory pathways, while also impairing tissue regeneration through the epigenetic silencing of stem cell plasticity factors [246]. Notably, the accumulation of epigenetic “noise” disrupts circadian regulation, DNA repair mechanisms, and mitochondrial function, creating a self‐reinforcing cycle that accelerates functional decline across multiple organ systems [74]. The resulting phenotypes include stem cell exhaustion, chronic low‐grade inflammation (“inflammaging”), and increased susceptibility to age‐related diseases such as neurodegeneration and cancer [68], with tissue‐specific epigenetic signatures emerging as molecular fingerprints of biological aging.

### 3.1 Genomic Instability and Impaired DNA Repair

The progressive accumulation of genomic instability and decline in DNA repair capacity constitute fundamental drivers of the aging process, creating a vicious cycle that accelerates cellular and organismal decline [247, 248]. As organisms age, their genomes become increasingly susceptible to various forms of damage [249], with DSBs representing particularly deleterious lesions that accumulate in multiple tissues including brain, liver, and hematopoietic systems [54]. This damage accrual stems from both increased genotoxic stress and diminished repair capacity—endogenous sources like ROS and replication stress combine with exogenous factors such as UV radiation to overwhelm protective mechanisms [250, 251]. Crucially, the efficiency of DNA repair pathways including nonhomologous end joining and homologous recombination declines with age due to epigenetic silencing of repair genes like BRCA1 [252] and ATM [253], reduced expression of critical proteins such as Ku70/80 and RAD51 [254], and NAD+ depletion impairing SIRT1/SIRT6 function [255, 256]. The consequences of this repair‐deficit are severe: unrepaired DSBs lead to chromosomal translocations, telomere dysfunction, and permanent cell cycle arrest, while misrepaired breaks generate mutagenic outcomes that further destabilize the genome [257].

Epigenetic dysregulation plays a central role in this process, both as a cause and consequence of genomic instability. Age‐related loss of heterochromatin marks like H3K9me3 and H3K27me3 permits activation of transposable elements (TEs) and repetitive sequences [258], creating new sites of genomic vulnerability. Simultaneously, epigenetic changes disrupt the precise coordination of DDR pathways—aberrant histone modifications alter the recruitment of repair factors to damage sites, while DNA methylation changes silence critical repair genes [63, 259]. This epigenetic deterioration creates a self‐reinforcing cycle where DNA damage induces epigenetic changes that in turn impair damage repair, leading to further genomic instability [260]. The functional consequences manifest across multiple biological levels: at the cellular level, persistent DNA damage triggers senescence or apoptosis; in stem cell populations, accumulated mutations impair regenerative capacity [261]; and at the tissue level, these changes contribute to age‐related pathologies including neurodegeneration, metabolic dysfunction, and increased cancer risk [262, 263].

Telomere shortening is a key biomarker for aging and age‐related tissue degeneration, as well as a driving factor for aging [264]. It triggers DDRs at the ends of chromosomes, promoting cellular aging and tissue dysfunction [265]. This process is tightly linked to epigenetic regulation—telomere attrition correlates with DNA hypomethylation [266, 267], while RNA methylation (e.g., m5C on TERC and m6A on TERRA) modulates telomerase activity and heterochromatin stability [268, 269, 270]. Key histone modifications, including H3K9me3 and H4K20me3, maintain telomeric integrity, with their age‐related loss exacerbating genomic instability. Additionally, alternative nucleic acid structures like G‐quadruplexes and R‐loops, which interact with epigenetic regulators, further contribute to replication stress and DNA damage accumulation in aging cells [271]. Together, telomere erosion and its associated epigenetic dysregulation create a self‐reinforcing cycle that accelerates functional decline.

Premature aging syndrome demonstrates the systematic nature of genomic instability, such as premature aging, Werner syndrome, Bloom syndrome, Cochrane syndrome, Seckel syndrome, and hair sulfur malnutrition [272]. Among them, genetic defects in the DNA repair pathway reproduced the phenotype of accelerated aging [273]. While these syndromes demonstrate the catastrophic consequences of repair deficiency, they also reveal that simple enhancement of DNA repair may not suffice to extend lifespan, highlighting the complexity of aging as a system‐wide phenomenon.

### 3.2 Transcriptional Dysregulation and Loss of Cellular Identity

The aging process is fundamentally linked to progressive transcriptional dysregulation mediated through multiple epigenetic mechanisms [274, 275]. A central feature is the activation of TEs [276], which constitute 30–80% of eukaryotic genomes [277]. Normally silenced by heterochromatin marks like H3K9me3 [278], TEs become derepressed during aging due to heterochromatin loss and decreased SIRT6‐mediated repression [279]. This TE activation creates genomic instability through insertional mutagenesis and disrupts normal gene expression patterns [280]. The consequences are particularly evident in neurodegenerative diseases and cellular senescence, where increased chromatin accessibility at retrotransposon sites drives further transcriptional dysregulation [281, 282].

Transcriptional precision deteriorates significantly with age through both chromatin‐based and epitranscriptomic mechanisms. Key histone modifications like H3K36me3, which normally suppress cryptic intragenic transcription, decline with age, leading to spurious transcript production. This loss of transcriptional fidelity is conserved from yeast to mammals and correlates with reduced lifespan [283]. Concurrently, age‐related changes in RNA modifications (the “epitranscriptome”) further disrupt gene expression regulation [284]. rRNA methyltransferases like NSUN5 influence lifespan through translational control, while mRNA m6A modifications affect neuronal function and stress resistance [285, 286]. These multilayered regulatory failures create increasing transcriptional noise, particularly in postmitotic cells like cardiomyocytes, though the effects appear cell‐type specific with HSCs showing different patterns of age‐related transcriptional change [287].

The cumulative effect of age‐related transcriptional dysregulation is profound loss of cellular identity and function. Upregulation of repeat elements and ribosomal protein genes, coupled with suppression of DNA repair pathways, creates a maladaptive cellular state [288]. In stem cells like HSCs, this leads to impaired regenerative capacity and skewed differentiation potential [289]. The inflammatory tone from SASP and other age‐related transcriptional changes contributes to chronic low‐grade inflammation (“inflammaging”), while stochastic expression noise reduces cellular fitness [290]. Importantly, these transcriptional alterations appear mechanistically linked through chromatin state changes, suggesting that interventions targeting epigenetic regulators could potentially restore more youthful transcriptional patterns and ameliorate age‐related functional decline [289].

### 3.3 Stem/Progenitor Cell Exhaustion and Regenerative Decline

The functional decline of stem and progenitor cells during aging manifests through two key mechanisms: depletion of dedicated stem cell pools and loss of injury‐induced cellular plasticity. Tissue‐specific stem cells, such as muscle satellite cells and HSCs, exhibit reduced self‐renewal capacity and biased differentiation with age. For example, aged HSCs show myeloid skewing that compromises adaptive immunity, while muscle stem cells lose regenerative potential, contributing to sarcopenia [291]. Epigenetic alterations, including aberrant DNA methylation (e.g., DNMT3a/3b dysregulation) and histone modifications, underlie this dysfunction by silencing stemness genes (OCT4, NANOG) while activating senescence pathways (p16INK4a) [292, 293]. The stem cell niche further exacerbates this decline through chronic inflammation (“inflammaging”) and extracellular matrix remodeling, creating a hostile microenvironment that impairs stem cell maintenance and activation. These changes collectively reduce tissue homeostasis and predispose to age‐related pathologies like anemia, immune senescence, and muscle wasting [291].

The loss of cellular plasticity represents an equally critical aspect of regenerative decline. While most organs maintain some basal renewal capacity, injury repair often depends on facultative reprogramming of differentiated cells—a process that becomes severely impaired with age. In young organisms, tissues like liver, lung, and intestine can activate latent regenerative programs through dedifferentiation (e.g., hepatocytes re‐entering cell cycle) or transdifferentiation (e.g., alveolar type II cells repairing lung epithelium) [3]. This plasticity requires precise epigenetic regulation, including DNA demethylation (mediated by TET enzymes) and chromatin remodeling at embryonic gene loci [294]. However, aging introduces epigenetic barriers—such as hypermethylation of plasticity genes and accumulation of repressive histone marks—that lock cells in differentiated states [22]. The resulting failure to mount adequate repair responses leads to prolonged recovery after injury and increased fibrosis, particularly in organs with low baseline turnover like heart and brain. Notably, this plasticity loss often precedes overt stem cell depletion, suggesting it may be the primary driver of age‐related regenerative failure [295].

### 3.4 Mitochondrial and Metabolic Dysfunction

Age‐related epigenetic alterations also drive the onset of mitochondrial dysfunction. Existing studies have shown that intragenic hypomethylation of the SNCA gene (encoding α‐synuclein [SNCA]) promotes abnormal SNCA aggregation, which—in synergy with oxidative stress and mitochondrial dysfunction—accelerates PD pathology. Likewise, hypomethylation of the PARK2 gene disrupts parkin expression, leading to decreased mitochondrial membrane potential, reduced ATP levels, and mitochondrial fragmentation [296]. HDACis induce hyper‐acetylation of histones H2, H3, H4, and the p300 promoter region associated with SNCA, thereby diminishing sirtuin 1/2 activity, impairing mitochondrial biogenesis, and fostering SNCA aggregation [297]. Multiple miRNAs (e.g., miR‐485, miR‐366a) downregulate PGC‐1α, curtailing the expression of mitochondrial biogenesis‐related genes (e.g., Nrf1/2, SIRT‐3) and exacerbating mitochondrial dysfunction [298]. At the same time, oxidative stress triggers epigenetic modifications (such as DNA methylation and ncRNA regulation) that alter the expression of relevant genes, further impairing mitochondrial function and elevating oxidative stress levels.

Mitochondrial dysfunction emerges as a central driver of aging, manifesting through progressive bioenergetic failure and disruptive signaling cascades. As the cell's powerhouses, mitochondria in aged tissues accumulate debilitating mutations in mtDNA while exhibiting impaired proteostasis and diminished quality control through reduced mitophagy [299]. These defects lead to a vicious cycle of metabolic insufficiency—respiratory chain complexes destabilize, membrane potentials decline, and ROS production escalates [300]. The consequences are particularly severe in high‐energy tissues: neuronal mitochondria show reduced axonal transport capacity, cardiac mitochondria demonstrate impaired calcium handling, and muscle mitochondria lose oxidative phosphorylation efficiency [301, 302]. Beyond energy deficits, damaged mitochondria become pathological signaling hubs, releasing proinflammatory mitochondrial DNA that activates cytosolic sensors like cGAS–STING and leaking intermembrane proteins that trigger apoptotic and pyroptotic cell death pathways [303, 304]. This mitochondrial distress propagates cellular senescence while creating an inflammatory microenvironment that further disrupts tissue homeostasis. The metabolic inflexibility of aged cells is compounded by declining NAD^+^ levels, which impair sirtuin activity and disrupt critical processes including DNA repair and stress resistance [305, 306].

The systemic repercussions of mitochondrial decline manifest through interconnected metabolic disturbances. Age‐related shifts in nutrient sensing pathways (e.g., mTOR, AMPK) alter substrate utilization, promoting insulin resistance and lipid accumulation even in nonadipose tissues. Hepatic mitochondria lose fatty acid oxidation capacity, contributing to age‐related steatosis, while pancreatic β‐cell mitochondria fail to adequately couple glucose metabolism to insulin secretion [307, 308, 309]. Neurons become particularly vulnerable as mitochondrial trafficking defects impair synaptic maintenance and antioxidant defenses weaken. These tissue‐specific manifestations share common roots in deteriorating mitochondrial membrane integrity, redox imbalance, and failing quality control mechanisms [310]. Notably, the accumulation of oxidized macromolecules and lipofuscin in lysosomes further compromises cellular clearance capacity, creating a self‐reinforcing cycle of metabolic dysfunction [311]. The resulting bioenergetic crisis not only limits tissue repair but also alters systemic metabolism, predisposing to characteristic aging phenotypes including sarcopenia, cognitive decline, and cardiovascular disease [312]. These pathological changes are exacerbated by the age‐related decline in mitochondrial‐encoded microproteins like humanin and MOTS‐c, which normally serve as systemic regulators of metabolic homeostasis and stress resistance [313].

### 3.5 Contribution to Major Age‐Related Pathologies

Epigenetic alterations play a pivotal role in cancer development by disrupting normal gene expression patterns that regulate cell proliferation, apoptosis, and DNA repair [314]. Age‐related DNA hypomethylation can lead to genomic instability and activation of oncogenes, while hypermethylation of tumor suppressor gene promoters (e.g., p16INK4a, BRCA1) silences their expression, facilitating uncontrolled cell growth [315, 316]. Histone modifications, such as loss of H4K16 acetylation and H3K27 trimethylation, contribute to chromatin compaction and transcriptional repression of critical anticancer genes [317, 318, 319]. Additionally, dysregulation of ncRNAs (e.g., miR‐21, miR‐155) promotes tumorigenesis by modulating oncogenic signaling pathways (e.g., PI3K/AKT, Wnt/β‐catenin) [320, 321]. The SASP in aged cells further exacerbates chronic inflammation, creating a tumor‐permissive microenvironment [322]. Epigenetic therapies, such as DNMT inhibitors (DNMTis) (e.g., 5‐azacytidine) and HDACis (e.g., vorinostat), are being explored to reverse these aberrations and restore normal gene function in cancer cells [323].

In AD, epigenetic dysregulation contributes to amyloid‐β (Aβ) accumulation and tau hyperphosphorylation. Age‐related DNA methylation changes in genes like BACE1 and APP enhance Aβ production [324], while histone deacetylation (e.g., reduced SIRT1 activity) impairs synaptic plasticity and memory formation [325]. Hypomethylation of the MAPT gene increases tau expression, promoting neurofibrillary tangle formation [326]. Additionally, oxidative stress‐induced DNA damage in neurons accelerates epigenetic drift, exacerbating neurodegeneration [327].

In PD, SNCA gene overexpression due to hypomethylation leads to Lewy body formation [328]. Epigenetic silencing of PARK2 (Parkin) and PINK1 disrupts mitophagy, causing mitochondrial dysfunction and dopaminergic neuron death [296]. HDACis show promise in reducing SNCA aggregation, while restoring DNA methylation patterns may slow PD progression [329].

Epigenetic mechanisms drive endothelial dysfunction, vascular inflammation, and atherosclerosis. Hypomethylation of proinflammatory genes (IL‐6, MCP‐1) and hypermethylation of endothelial nitric oxide synthase impair vasodilation and promote plaque formation [330]. HDACs contribute to VSMC senescence and arterial stiffening, while SIRT1/SIRT6 deficiency accelerates vascular aging [331]. ncRNAs (miR‐217, miR‐34a) further exacerbate oxidative stress and endothelial senescence [332, 333]. Epigenetic interventions targeting DNA methylation (e.g., folate supplementation) or HDAC inhibition may mitigate CVD progression [334].

Epigenetic changes in pancreatic β‐cells and adipose tissue contribute to insulin resistance and T2DM. Hypermethylation of PDX‐1 and GLUT4 impairs insulin secretion and glucose uptake [335], while hypomethylation of inflammatory genes (TNF‐α, IL‐1β) promotes chronic low‐grade inflammation [336]. Age‐related loss of SIRT1 and SIRT3 disrupts mitochondrial function, exacerbating oxidative stress in metabolic tissues [337]. Additionally, miRNA dysregulation (miR‐375, miR‐29) alters insulin signaling pathways. Epigenetic reprogramming via caloric restriction (CR) or NAD+ boosters (e.g., NMN) shows potential in restoring metabolic homeostasis [338].

Epigenetic modifications (DNA methylation, histone acetylation) drive hepatic lipid accumulation and fibrosis. Hypomethylation of PPARγ2 promotes steatosis [339], while hypermethylation of CPT1A reduces fatty acid oxidation [340]. SIRT1/SIRT3 downregulation impairs mitochondrial function, increasing ROS production and hepatocyte senescence [341]. Dysregulated miRNAs (miR‐34a, miR‐122) further exacerbate inflammation and fibrogenesis [342]. Epigenetic therapies targeting DNMTs or HDACs may prevent NAFLD progression to cirrhosis [341].

Epigenetic dysregulation plays a critical role in osteoporosis, a disease characterized by decreased bone mineral density and increased fracture risk in aging individuals. Key mechanisms include DNA methylation changes in osteogenic genes (e.g., RUNX2, SP7/Osteri), which impair osteoblast differentiation and bone formation [343]. Additionally, histone deacetylation (mediated by HDACs) suppresses Wnt/β‐catenin signaling, a crucial pathway for bone formation, while miRNA dysregulation (e.g., miR‐214 upregulation) inhibits osteoblast activity [344, 345]. Senescent osteocytes and mesenchymal stem cells (MSCs) contribute to bone loss via the SASP, secreting factors like RANKL that enhance osteoclastogenesis [346]. SIRT1 and SIRT6, key epigenetic regulators of bone homeostasis, decline with age, leading to impaired DNA repair and increased oxidative stress in bone tissue [347]. Therapeutic strategies targeting epigenetic modifications—such as HDACis, SIRT1 activators, and DNA methylation modulators—are being explored to restore bone remodeling balance and prevent osteoporosis progression [348, 349].

## 4 Epigenetic Rejuvenation Strategies

**FIGURE 4:**
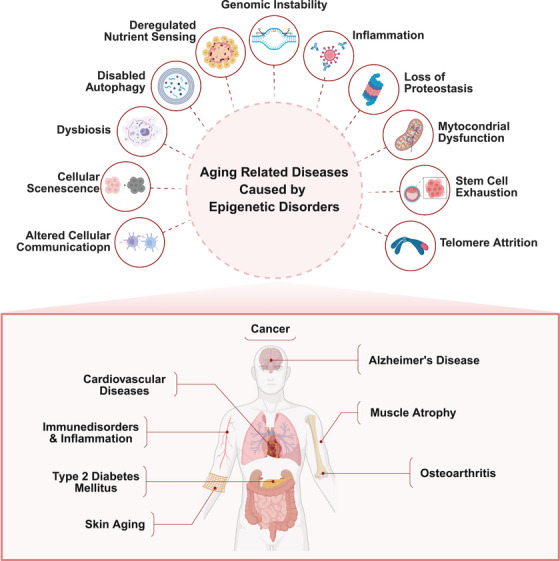
Aging‐related diseases caused by epigenetic disorders. This diagram highlights various biological processes and mechanisms linked to aging‐related diseases that are influenced by epigenetic disorders. Key processes include altered cellular communication, cellular senescence, dysbiosis, disabled autophagy, deregulated nutrient sensing, genomic instability, inflammation, loss of proteostasis, mitochondrial dysfunction, stem cell exhaustion, and telomere attrition. These disruptions contribute to the development of aging‐related diseases such as cancer, Alzheimer's disease, cardiovascular diseases, immune disorders, inflammation, muscle atrophy, type 2 diabetes mellitus, osteoarthritis, skin aging, and other health conditions. This figure was created by BioRender.

**FIGURE 5:**
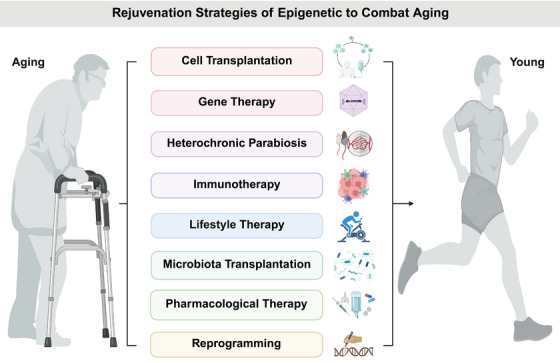
Rejuvenation strategies of epigenetic to combat aging. This diagram presents various therapeutic strategies aimed at combating aging and age‐related diseases. These approaches include cell transplantation, gene therapy, heterochronic parabiosis, immunotherapy, lifestyle therapy, microbiota transplantation, pharmacological therapy, and reprogramming. Each strategy targets different aspects of aging and seeks to rejuvenate or repair damaged systems, ultimately improving health outcomes and promoting longevity. The diagram contrasts the effects of aging in individuals with these potential interventions that aim to reverse or mitigate age‐related decline. This figure was created by BioRender.

Understanding aging has undergone a transformation in recent years, largely due to the increasing recognition of the role that epigenetic modifications play in the aging process. These changes, including DNA methylation, histone modifications, and ncRNA regulation, accumulate over time and contribute to aging‐related diseases (Figure 4). Importantly, epigenetic modifications are reversible, making them an ideal target for rejuvenation therapies aimed at reversing or delaying the decline associated with aging. This section will continue to explore the most promising rejuvenation strategies, including cellular reprogramming, CRISPR‐based epigenetic editing, pharmacological interventions, and lifestyle changes (Figure 5).

### 4.1 Reprogramming‐Based Approaches (Induced Pluripotent Stem Cell)

Reprogramming senescent cells and induced pluripotent stem cell (iPSC) technology have emerged as significant breakthroughs in the fields of antiaging and regenerative medicine in recent years. The core principle of these approaches is to reverse the cellular aging state and restore tissue function through epigenetic reprogramming. This strategy has shown potential in a variety of aging‐related diseases, including neurodegenerative diseases, cardiovascular diseases, osteoarthritis, and fibrotic diseases.

Studies have demonstrated that the transient expression of OSKM (Oct4, Sox2, Klf4, and c‐Myc) can initiate extensive chromatin remodeling of target regions, enabling the reprogramming of terminally differentiated somatic cells into pluripotent cells and the rejuvenation of human senescent fibroblasts [350]. Ectopic expression of OSK (without c‐Myc) can restore DNA methylation in retinal ganglion cells of mice, improving vision problems in aged mice with glaucoma. In addition, cyclic reprogramming technology, through transient expression of the NANOG gene, can significantly enhance muscle regeneration in mouse models while preserving tissue‐specific gene expression [351].

In terms of disease applications, the safety and potential efficacy of human embryonic stem cell‐derived dopaminergic neurons transplanted into patients with PD have been reported [352]. Another clinical trial involved the transplantation of allogeneic iPSC‐derived dopaminergic progenitors into the bilateral putamen of seven patients with PD. The results showed that among six patients, four had significant improvements in motor symptoms (MDS‐UPDRS scores) and a 44.7% increase in dopamine synthesis capacity. These studies provide important evidence for the use of iPSCs in the treatment of PD and may become an alternative to traditional therapies in the future [353].

However, the field still needs to address the balance between reprogramming efficiency and tumorigenicity. Future directions include the development of small molecules to replace reprogramming factors and the optimization of personalized treatment plans using organ‐on‐a‐chip models. Overall, the technology of reprogramming senescent cells is moving from proof‐of‐concept to clinical translation, but further research is needed to support its long‐term safety and in‐depth mechanistic understanding.

### 4.2 Pharmacological and Metabolic Interventions

Epigenetic regulation, as a key driving force of aging, has in recent years become an important target for the development of antiaging drugs. Strategies based on epigenetic reprogramming, histone modification regulation, and DNA methylation intervention have shown significant therapeutic potential in a variety of aging‐related diseases. Studies have shown that suberoylanilide hydroxamic acid, a HDACi, can prevent premature skin aging associated with Cockayne syndrome [354]. The HDACi ITF2357 can inhibit diastolic dysfunction induced by aging in mice [355]. Sodium butyrate, an HDACi, can treat muscle atrophy caused by aging in mice [356]. The latest research has found that vorinostat, an HDACi and a novel hormone‐like substance, can extend the lifespan of nematodes and enhance stress resistance by activating the SKN‐1 pathway [357]. Similarly, dihydromyricetin (DHM) is an inhibitor of DNMT1. Studies have shown that DHM can act as an epigenetic inhibitor with antiaging effects and has regenerative effects on aging human skin [358].

Sirtuins are a class of proteins that regulate cellular processes such as metabolism, DNA repair, and stress response, and are key participants in the aging process. Sirtuin activators such as resveratrol and NAD^+^ precursors (e.g., nicotinamide riboside and NMN) have attracted attention for their potential to reverse aging cells. Resveratrol has been shown to activate SIRT1, an important sirtuin in regulating DNA repair and inflammation. Studies have shown that resveratrol not only extends lifespan but also restores cell function by modulating epigenetic marks, including histone acetylation and DNA methylation [1]. With increasing age, NAD^+^ levels decline, impairing mitochondrial function and DNA repair. NAD^+^ precursors, such as NMN and NR, have been shown to restore NAD^+^ levels, activate sirtuins, and improve cell function [142]. In mammals, supplementation with NR can enhance mitochondrial function and extend the lifespan of mice. Moreover, filling NAD^+^ with NMN or NR can improve meibomian gland dysfunction associated with aging in elderly mice and improve cognitive function in an AD mouse model. Clinical trials have also validated the efficacy of NAD^+^ supplements, showing that 8 weeks of NMN safely and effectively promoted NAD^+^ biosynthesis in healthy middle‐aged men and alleviated postprandial hyperinsulinemia [359]. Another clinical study showed that overweight, obese, and middle‐aged or older adults taking MIB‐626 (precursor β‐NMN) can safely increase circulating NAD^+^ levels and significantly reduce total LDL and non‐HDL cholesterol, weight, and diastolic blood pressure [360].

Metformin and rapamycin, drugs known for treating other diseases, have become potential antiaging drugs. Metformin, widely used for treating type 2 diabetes, has been shown to activate AMPK, thereby improving cell function and extending lifespan, and reducing the epigenetic age of model organisms [361]. Rapamycin, an mTOR inhibitor, has been shown to extend the lifespan of various organisms and improve cellular aging through epigenetic regulation [362, 363].

Despite the promising prospects of epigenetic drugs, their clinical application still faces challenges. Tissue‐specific delivery and dose control are key to avoiding off‐target effects, while epigenetic drugs exhibit cellular heterogeneity in response. Future development directions include: (1) developing small‐molecule modulators targeting specific epigenetic marks; (2) optimizing spatiotemporal specificity control strategies, such as optogenetic epigenetic editing systems; (3) exploring the synergistic effects of epigenetic drugs with other antiaging therapies. With the development of precise assessment tools such as the epigenetic clock, personalized antiaging treatments are gradually becoming a reality.

### 4.3 Lifestyle and Systemic Modulation

#### 4.3.1 Lifestyle Therapy

Recent studies have shown that lifestyle interventions are powerful nonpharmacological means that target fundamental biological processes to combat aging and promote tissue regeneration. These interventions include dietary adjustments, exercise programs, sleep optimization, and stress reduction. They work through epigenetic remodeling, mitochondrial enhancement, and the clearance of senescent cells, providing protection against age‐related diseases such as cardiovascular diseases, neurodegenerative diseases, and metabolic syndromes.

CR and fasting‐mimicking diets (FMDs) have become the most potent dietary interventions, showing consistent life‐extending effects across various species. Under CR conditions, the epigenetic age of animals is significantly reduced. Studies have shown that interventions with CR and FMDs can change various epigenetic features in mice, such as DNA methylation and histone modifications [364, 365]. Moreover, the reason why CR and FMDs work in various aging‐related diseases is also partly due to the epigenetic changes they cause [366]. In addition, the CALERIE‐2 trial showed that reducing caloric intake by 25% in humans over 2 years can reduce epigenetic age and improve inflammatory markers [367].

Nutritional interventions play a key role in the aging process. Studies have shown that diets rich in antioxidants and anti‐inflammatory nutrients (such as the Mediterranean diet) can slow down epigenetic aging [368, 369]. In addition, short‐term vegetarian diets are associated with a reduction in DNA methylation age [370]. Specific nutrients, such as omega‐3, vitamin D, and antioxidants, also show potential to delay aging by changing epigenetics [371]. Therefore, personalized nutritional interventions are considered an important strategy for antiaging.

Time‐restricted eating (TRE) has gained attention for its metabolic benefits. Studies have shown that early TRE (an 8‐h eating window before 3 p.m.) improves beta‐cell function in prediabetic patients through epigenetic regulation (BMAL1/CLOCK) compared with the control group [372, 373]. In addition, fasting can extend the lifespan of various model organisms and improve brain and immune system functions by changing epigenetics [66, 374].

Exercise interventions show specific tissue rejuvenation effects. Studies have shown that regular exercise can slow down epigenetic aging by delaying immune aging and reducing cardiovascular risks. It can also enhance memory in mice through epigenetic changes [375, 376]. In addition, sleep optimization has been proven to delay aging through epigenetic changes [47, 377].

These lifestyle interventions target multiple hallmarks of aging. Their clinical applications range from metabolic diseases and neurodegenerative diseases to cardiovascular diseases. Their combined effects may provide synergistic benefits. Combined interventions (CR + exercise + stress reduction) reduced biological age by 3.2 years [378]. Although there are still key challenges in implementation fidelity and personalized optimization, the existing evidence strongly supports the use of lifestyle interventions as the preferred strategy for healthy aging. Future research should focus on precision lifestyle medicine methods targeting individual genetic and epigenetic characteristics.

#### 4.3.2 Microbiota Transplantation

In recent years, fecal microbiota transplantation (FMT) based on epigenetic regulation has shown potential as an antiaging intervention strategy in the treatment of various aging‐related diseases. Research has found that specific types of gut bacteria are potentially causally associated with EAA. For example, Holdemania_unclassified is positively correlated with GrimAge acceleration [379]. Fecal or microbial transplantation has been shown in multiple studies to delay aging, including skin aging, ovarian aging, and brain aging, and has also demonstrated its role in treating aging‐related diseases. The gut microbiota mainly exerts its effects through its metabolites [380, 381, 382]. Moreover, metabolites of the gut microbiota can improve neurological diseases by altering DNA methylation, histone modifications, and ncRNA [382]. These studies have also shown the great potential of FMT in regulating epigenetics. However, there is currently a lack of direct evidence linking the gut microbiota to epigenetic modifications. To fully understand the interplay between the gut microbiota, epigenetics, and aging, more in‐depth basic and clinical research is needed. Future research should also focus on the standardization of FMT procedures, the prevention of immune rejection reactions, and the assessment of long‐term safety.

### 4.4 Novel and Emerging Techniques

#### 4.4.1 Gene Therapy

In recent years, gene‐editing technologies (such as CRISPR–Cas9, base editing, and epigenetic editing) have become core tools for aging intervention and regenerative medicine. By precisely modifying aging‐related genes, epigenetic markers, or mitochondrial DNA, these techniques can reverse or delay cellular aging and promote tissue repair. This strategy has shown potential in various aging‐related diseases. Currently, CRISPR technology has also been applied to epigenetics‐related genes. For example, HAT KAT7, the inactivation of KAT7 reduces the acetylation of histone H3 lysine 14 and alleviates the aging of hMPCs. Moreover, intravenous injection of lentiviral vectors encoding Cas9/sg‐Kat7 can extend the lifespan and improve the healthspan of mice [30]. In addition, studies have shown that gene therapy using lentiviruses carrying SOX5 or HMGB2 can improve cellular aging through epigenetic regulation, rejuvenate cartilage in aged mice, and alleviate osteoarthritis [383]. However, the long‐term safety and off‐target effects of gene editing still need to be optimized. Single‐cell sequencing has revealed that the genomic instability of senescent cells may affect editing efficiency. Future directions include developing CRISPR systems driven by aging cell‐specific promoters and transient editing strategies to avoid the risks associated with persistent genetic modification. Overall, gene‐editing technologies are moving from basic research toward clinical translation, but their precision, delivery efficiency, and long‐term safety in aging intervention still need further exploration.

#### 4.4.2 Heterochronic Parabiosis

Heterochronic parabiosis is a technique that forms a physiological connection between young and old individuals (or animal models) through the sharing of blood circulation. In recent years, studies have shown that its antiaging effects are closely related to epigenetic reprogramming. This strategy systemically regulates DNA methylation, histone modifications, and ncRNA networks to reverse the expression of aging‐related genes and promote tissue regeneration. Research has shown that young plasma can activate the epigenetic rejuvenation program in aged tissues, significantly reducing the epigenetic age of the liver and extending the lifespan of mice [384, 385]. Similarly, exosomes derived from young mouse adipose‐derived MSCs (ADSCs) help to improve aging signs in old mice and reduce the epigenetic age of aged mice [386]. However, the clinical application of heterochronic parabiosis still faces challenges, such as the risk of immune rejection and long‐term safety issues. Future directions include the development of drugs that target epigenetic factors to mimic its anti‐aging effects.

## 5 Assessing Rejuvenation: Epigenetic Biomarkers and Models

**FIGURE 6:**
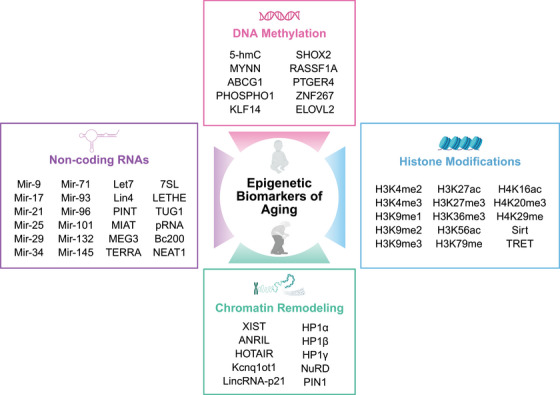
Epigenetic biomarkers of aging. This scheme enumerates the key epigenetic biomarkers of aging described in the current study, categorized into four major groups. This figure was created by BioRender. *Abbreviations*: 5‐hmC, 5‐hydroxymethylcytosine; MYNN, myoneurin; mCH, non‐CG methylation; ABCG1, ATP‐binding cassette sub‐family G member 1; PHOSPHO1, phosphoethanolamine/phosphocholine phosphatase 1; KLF14, Kruppel‐like factor 14; SHOX2, short stature homeobox 2; RASSF1A, ras association domain family member 1 isoform A; PTGER4, prostaglandin E receptor 4; ZNF267, zinc finger protein 267; ELOVL2, elongation of very long chain fatty acids protein 2; H, histone; K, lysine; ac, histone acetylation; me, methylation; TERT, telomerase reverse transcriptase; Sirt1, sirtuin; XIST, x inactive specific transcript; ANRIL, antisense noncoding rNA in the ink4 locus; HOTAIR, hox transcript antisense intergenic RNA; Kcnq1ot1, KCNQ1 opposite strand antisense transcript 1; LincRNA, long intergenic noncoding RNA; HP1, heterochromatin protein 1; NuRD, nucleosome remodeling and histone deacetylase; PIN1, peptidylprolyl Isomerase 1; Let7, lethal‐7; Lin4, lethal in 4; PINT, PIN2 interacting noncoding transcript; MIAT, myocardial infarction associated transcript; MEG3, maternally expressed gene 3; TERRA, telomeric repeat‐containing RNA; 7SL, seven sl RNA; LETHE, lethal(2) giant larvae homolog 1, endoplasmic reticulum; TUG1, taurine upregulated gene 1; pRNA, paclitaxel‐resistant RNA; Bc200, brain cytoplasmic RNA 200; NEAT1, nuclear enriched abundant transcript 1.

Epigenetic biomarkers offer a powerful tool to measure biological aging, predict health outcomes, and assess the efficacy of rejuvenation therapies (Figure 6). Advances in epigenetic clocks and single‐cell epigenomics have opened new avenues for understanding aging at both the organismal and cellular levels. These tools are transforming aging research and hold promise for precision medicine and therapeutic development.

### 5.1 Epigenetic Clocks and Multiomics Age Prediction

Epigenetic clocks are predictive algorithms based on DNA methylation patterns that estimate biological age, often reflecting physiological health better than chronological age. Early models, such as the Horvath and Hannum clocks, laid the groundwork for this field, but recent innovations have significantly enhanced their precision and utility [40, 41].

Second‐generation clocks like PhenoAge and GrimAge incorporate additional data, such as inflammatory markers and clinical measures, to predict biological aging more accurately [42, 43]. These clocks are highly correlated with healthspan, chronic disease incidence, and mortality risk. For example, GrimAge has been validated across diverse populations and has demonstrated its ability to predict outcomes such as time‐to‐death and cancer incidence with remarkable accuracy [43].

Technological advancements, such as the integration of deep learning algorithms, have further improved the resolution of epigenetic clocks. Tools like DeepAge analyze multiomics data, enabling predictions of biological age that account for complex interactions between epigenetic, transcriptomic, and proteomic changes [387]. Tissue‐specific clocks, such as those developed for brain and liver cells, provide insights into organ‐specific aging processes, which are critical for targeted therapies [388, 389, 390].

Epigenetic clocks are increasingly used to evaluate the success of rejuvenation interventions by measuring reductions in biological age. For example, partial cellular reprogramming with Yamanaka factors has been shown to reduce epigenetic age markers in fibroblasts, highlighting its potential to reverse cellular aging [391, 392, 393]. Similarly, CR and intermittent fasting have demonstrated measurable reductions in biological age as indicated by PhenoAge and GrimAge [378, 394]. Clinical trials are now incorporating epigenetic clocks to monitor the impact of therapies like senolytics, NAD^+^ precursors, and CRISPR‐based gene editing. For instance, a study on NMN supplementation showed significant improvements in biological age, as reflected by reduced DNA methylation age in muscle and liver tissues [395]. The use of these clocks not only validates the efficacy of interventions but also provides feedback for refining personalized rejuvenation protocols.

### 5.2 Single‐Cell and Spatial Epigenomic Profiling

Aging is a heterogeneous process, affecting different tissues and cell types in unique ways. Single‐cell epigenomics has emerged as a transformative technology for studying these cell‐specific changes. Techniques like single‐cell DNA methylation sequencing and single‐cell ATAC‐seq have enabled researchers to map the epigenetic landscape at an unprecedented resolution [396, 397]. For example, studies on HSCs have revealed distinct methylation signatures associated with age‐related declines in regenerative capacity [398, 399]. Similarly, single‐cell analyses of brain tissues have identified neuronal and glial populations with divergent aging trajectories, highlighting the vulnerability of certain cell types to age‐related diseases [400, 401]. These insights are critical for understanding the interplay between cellular heterogeneity and aging. By identifying epigenetic markers specific to vulnerable cell populations, researchers can develop targeted interventions to mitigate age‐associated decline.

The precise positioning and spatial relationships of cells also greatly impact aging. Spatial transcriptomics is a new method that can capture gene expression and epigenetic data linked to spatial information, offering the exact location of these elements within tissues. For example, in muscle aging research, this technology has revealed key mechanisms like fiber‐specific degeneration and satellite cell dysfunction. In senescent cells, heterochromatin decreases while euchromatin increases. The loss of heterochromatin near the nuclear membrane causes genomic instability and triggers aging‐related diseases. Spatial epigenomics allows for a more precise analysis of chromatin state changes in different nuclear regions. It shows how these changes affect gene expression and cell function, thus uncovering the fundamental molecular mechanisms of aging.

Antiaging therapies often have cell type‐specific effects, which can be clarified through single‐cell and spatial epigenomics. For example, partial reprogramming has been shown to rejuvenate fibroblasts more effectively than neurons, highlighting differences in epigenetic plasticity across cell types [402, 403]. Similarly, senolytics selectively clear senescent cells, preserving healthy populations and improving overall tissue function [404, 405].

Recent studies using single‐cell technologies have demonstrated that CR alters chromatin accessibility in energy‐regulating pathways in muscle cells while restoring DNA methylation patterns in neurons associated with memory and learning [406, 407]. These findings underscore the potential of tailoring rejuvenation strategies to the specific needs of different tissues. The integration of single‐cell and spatial epigenomics in clinical research is paving the way for precision medicine. By capturing the nuanced effects of therapies at the cellular level, this approach ensures that interventions are both effective and safe, minimizing off‐target effects while maximizing therapeutic benefits.

### 5.3 Model Systems for Efficacy Evaluation

In the field of antiaging research, the development of effective and safe intervention measures is one of the core goals. To achieve this goal, it is essential to establish scientifically reliable efficacy evaluation models to accurately assess the short‐term and long‐term effects of antiaging interventions. A good efficacy evaluation model should not only consider changes in biomarkers but also integrate multiple aspects, including physiological function, healthspan, and lifespan.

#### 5.3.1 DNA Methylation Clock Model System

Among the existing efficacy evaluation systems, the first DNA methylation clock was the Elastic Model established in 2011, which used approximately 100 saliva samples to demonstrate highly accurate prediction of chronological age [408]. In 2013, researchers constructed the Hannum clock (71‐CpG clock) using 656 whole blood samples [41]. Subsequently, the pan‐tissue Horvath clock (353‐CpG clock) was developed using 8000 samples from 51 different tissues and cell types. Additionally, multiple epigenetic clocks have been established, including the 3‐CpG clock, 99‐CpG clock, and 8‐CpG clock [361]. These DNA methylation clocks can predict the age of different tissue types, demonstrating their ability to measure aging signals shared among various cell types.

However, the first‐generation methylation clocks did not link many important characteristics of biological aging. Researchers then developed the DNAm PhenoAge clock, which uses a standard method to predict a composite biological age score based on chronological age and a linear combination of nine clinical parameters associated with mortality risk [409]. The DNAm GrimAge clock was constructed through a two‐stage process to predict biological age: first, models were developed to predict smoking pack‐years and the concentrations of seven plasma proteins known to be associated with mortality risk, and then these model outputs were combined into a clock to predict time to death [43]. By design, all these clocks have strong correlations with mortality risk, and some are also associated with cardiovascular disease risk, physical function (balance, grip strength, walking speed), and several healthy blood chemistry indicators [410]. Recently, the DunedinPoAm DNAm clock was developed using a birth cohort from the same year (*n* = 810), tracking changes in clinical chemistry and physiological biomarkers of 18 organ functions collected at ages 26, 32, and 38 to quantify the comprehensive ratio of biological age. Compared with the DNAm PhenoAge clock, the DunedinPoAm DNAm clock has stronger and more significant associations with age‐related phenotypes, including physical function, cognition, self‐rated health, and mortality [411]. The use of epigenetic blood aging as a proxy for physiological aging in other organ systems highlights their potential as clinical biomarkers. However, current studies indicate that epigenetic clocks trained in specific tissues have stronger associations with the functional state of those tissues and weaker correlations with other tissues and overall weak indicators [412]. Therefore, the second‐generation clocks have limited ability to measure and explain the mechanisms of aging in specific tissues relative to other tissues.

#### 5.3.2 Proteomics Clock Model System

Research using various proteomics techniques has shown that proteins in plasma and cerebrospinal fluid change with age, which has spurred the development of proteomic aging clocks. In 2018, the first aging clock based on plasma proteins was developed, which studied the association between the proteomic age gap and biological aging and detected associations with many physiological and clinical aging phenotypes. This model also showed correlations with mortality, multimorbidity, healthspan, and lifespan [413]. Similarly, it is still unclear how much the changes in the proteome are correlated with overall aging. Additionally, the low level of development and updating of proteomics technology means that the application of proteomic clocks is less than that of methylation clocks. In addition, there are also metabolomics and glycomics clock models, but the application of other omics clocks is even less, and their validation is limited.

Aging clocks constructed from DNA methylation data, proteomics, and metabolomics have all demonstrated the ability to identify the state of biological aging. The second‐generation DNA methylation clocks are currently the most widely used and practical. However, the correlation between different molecular feature clocks is particularly low. In the future, integrating multiple omics layers into a composite model, such as a multiomics aging clock, will help to understand which molecular aging features are shared across omics layers or carry different phenotypic information [414]. With the development of various technologies (such as spatial epigenomics), more aging omics clocks will emerge, achieving more precise and comprehensive predictive effects. We believe that the development of aging clocks should move toward customization and combination. Aging clocks will also play an important role in advancing the science of aging and personalized medicine.

## 6 Challenges, Ethical Considerations, and Future Horizons

The role of epigenetics in aging and age‐related diseases has opened a new frontier in biomedical research. Epigenetic therapies, which target modifications such as DNA methylation, histone modifications, and ncRNAs, hold immense promise for extending healthspan and treating age‐related diseases. However, several challenges remain in the application of these therapies. In this section, we explore the limitations of current epigenetic therapies and propose future research directions to address these issues, with an emphasis on precision, safety, and efficacy.

### 6.1 Scientific and Technical Limitations

#### 6.1.1 Target Specificity and Off‐Target Effects

Achieving precise targeting of specific epigenetic marks is one of the most significant challenges in the field of epigenetic therapies. Epigenetic modifications, such as DNA methylation and histone modifications, occur at numerous sites across the genome, and modulating these marks in a precise manner without affecting other regions is difficult [415, 416]. For example, DNMTis and HDACis can lead to the silencing of specific genes, but they also have widespread effects on the genome. This can result in off‐target effects, such as the unintended activation or silencing of nontarget genes, which may have deleterious consequences for cellular function and overall health [417].

Recent advances in gene‐editing technologies, particularly CRISPR/Cas9‐based epigenetic editing tools, have provided a promising approach for achieving greater specificity. By fusing catalytically dCas9 with epigenetic modifiers like DNMTs or HATs, researchers have been able to target specific loci and modify epigenetic marks with improved precision [418, 419]. However, even with these improvements, challenges remain in delivering these tools to specific tissues, ensuring minimal off‐target effects, and achieving stable and long‐lasting changes in the epigenome without causing collateral damage [420, 421].

Moreover, ensuring tissue‐specificity for epigenetic therapies is a critical hurdle. In complex organisms like humans, tissues and cell types exhibit diverse epigenetic landscapes, and therapeutic interventions must be tailored to the specific requirements of each tissue. Efforts are underway to refine delivery systems, such as nanoparticles, viral vectors, and lipid‐based carriers, to improve the tissue‐specific delivery of epigenetic editing tools [422, 423, 424].

#### 6.1.2 Difficulty in Fully Reversing Age‐Related Epigenetic Changes

Age‐related epigenetic changes are complex and cumulative, involving DNA methylation drift, histone modification alterations, and chromatin remodeling. These modifications accumulate over time due to a combination of genetic, environmental, and lifestyle factors, leading to gene expression changes that contribute to the aging process and the onset of age‐related diseases [6, 9, 425]. While epigenetic therapies have demonstrated the potential to reverse some of these alterations, fully reversing the complex and multilayered epigenetic changes associated with aging remains a significant challenge [426, 427].

Certain epigenetic marks may become “locked in” over time, particularly those associated with cellular senescence and DDRs. For example, the DNA hypermethylation of tumor suppressor genes and the loss of histone acetylation in key genes responsible for stress responses are hallmarks of aging. These changes often lead to cellular dysfunction and the loss of regenerative capacity in tissues [428, 429]. While epigenetic therapies can target these alterations, they may not be sufficient to fully reverse the functional decline of aging cells.

Furthermore, epigenetic interventions aimed at reversing these changes may not fully restore cellular function, as aging is a multifactorial process that involves genetic, environmental, and cellular factors beyond the epigenome. For instance, cellular senescence, mitochondrial dysfunction, and telomere attrition are key drivers of aging, and epigenetic therapies alone may not be enough to address these factors comprehensively. Therefore, a more holistic approach that combines epigenetic therapies with other interventions, such as senolytics or mitochondrial rejuvenation, may be necessary to achieve effective rejuvenation [371, 430, 431].

#### 6.1.3 Tumorigenesis and Unintended Consequences

While epigenetic therapies are often seen as safer alternatives to genetic therapies, they are not without risks. One of the major concerns is the potential for tumorigenesis, as epigenetic reprogramming can lead to the activation of oncogenes or the silencing of tumor suppressor genes. For example, the reactivation of silenced tumor suppressor genes by DNA demethylation or histone acetylation could inadvertently increase the risk of cancer by disrupting the normal regulation of cell growth and division [432].

Furthermore, epigenetic reprogramming has the potential to alter the balance between cellular growth, apoptosis, and immune surveillance. Even minor disruptions in these pathways could lead to uncontrolled cell proliferation and the emergence of malignancies. Long‐term exposure to epigenetic therapies could also lead to genomic instability, which may further increase the risk of tumorigenesis.

Thus, while epigenetic therapies hold great promise for rejuvenation, it is critical to carefully monitor for potential adverse effects, including the risk of cancer. To minimize these risks, it will be essential to design therapies that selectively target aging‐related genes without affecting the overall stability of the genome. Additionally, combination therapies that incorporate tumor‐suppressive agents or immunotherapies may help mitigate the risk of tumorigenesis [19, 20].

### 6.2 Translational and Clinical Hurdles

#### 6.2.1 Case Studies of Successful Rejuvenation in Model Organisms

Preclinical studies have demonstrated the potential of epigenetic reprogramming in various model organisms, offering promising results for rejuvenation therapies. In one of the pioneering studies, Yamanaka factors (Oct4, Sox2, Klf4, and c‐Myc) were introduced into aged mice, effectively reversing certain age‐related changes in tissue regeneration and stem cell function [433, 434]. Furthermore, non‐human primates have also exhibited signs of rejuvenation when exposed to similar reprogramming treatments, with improvements in cellular function and tissue repair capacity [435, 436].

Despite these advancements, epigenetic reprogramming in humans faces challenges related to safety and efficiency. Reprogramming technologies must be refined to minimize the risks associated with tumorigenesis and uncontrolled cell proliferation [28, 29, 437]. Ongoing efforts are focused on optimizing the delivery mechanisms, ensuring the specificity of reprogramming factors, and reducing the risk of adverse outcomes.

#### 6.2.2 Clinical Trials Investigating Epigenetic Therapies for Age‐Related Diseases

**FIGURE 7:**
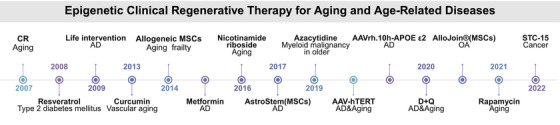
Epigenetic clinical regenerative therapy for aging and age‐related diseases. This timeline illustrates the development and application of various epigenetic‐based clinical regenerative therapies for aging and age‐related diseases over the years. The bolded black text represents the therapeutic methods, with the corresponding diseases listed below, and the numbers indicate the clinical application dates. They are arranged in chronological order of application. This figure was created by BioRender.

Over the past decade, a steady stream of antiaging drugs has emerged, many of which have already entered clinical trials (Figure 7 and Table 1). The clinical potential of epigenetic interventions has likewise been probed in multiple trials. For instance, HDACis are being investigated for their ability to reverse age‐related inflammation and the decline in tissue‐regenerative capacity; they do so by modulating histone modifications to boost the expression of genes involved in tissue repair while attenuating chronic inflammation [1]. These findings will expand the pool of candidate therapies for older adults and lay the groundwork for future antiaging strategies. Meanwhile, combining antiaging therapies with established interventions for age‐related diseases may yield superior outcomes compared with either approach alone. The opportunities and challenges ahead will center on identifying the optimal combination of synergistic treatments for each individual patient.

**TABLE 1:** Clinical research on aging and age‐related diseases.

Mechanism of action	Target/gene	Treatment	Clinical implication	Clinicaltrials.gov ID
Thyroid hormone receptor‐β agonist reducing DBI mRNA	Diazepam binding inhibitor (DBI)	Resmetirom	NCT05500222	Nonalcoholic liver disease
Senolytic therapy	Senescent cells	Dasatinib + quercetin	NCT05422885, NCT04685590	Alzheimer's disease
Reverse transcriptase inhibitor	LINE‐1	Emtricitabine	NCT04500847	Alzheimer's disease
		Lamivudine	NCT04552795, NCT06519357	
NAD+ precursors	NAD	Nicotinic acid	NCT05590468	Mitochondrial myopathy
		Nicotinamide riboside	NCT04528004	Systolic heart failure
		Nicotinamide riboside	NCT04430517	Alzheimer's disease
		Nicotinamide riboside	NCT04907110	Overweight, obesity, aging, and type 2 diabetes mellitus
		Nicotinamide riboside	NCT05593939	Aging
Probiotics	/	Bifidobacterium triple	NCT04017403	Postoperative cognitive dysfunction in >65 years old after orthopedic surgery
Anti‐inflammatories	COX‐1/2	Aspirin	NCT03480776, NCT02804815, NCT02965703	Cancer
	IL‐1β	Canakinumab	NCT04795466	Mild Alzheimer's disease
	IL‐1β	Canakinumab	NCT01327846	Osteoarthritis
	IL‐1α/β	Lutikizumab	NCT02087904	Osteoarthritis
	/	Mesenchymal stem cell therapy	NCT02645305, NCT01559051	Chronic obstructive pulmonary disease
Lifestyle intervention	DNA methylation	Fasting mimicking diet and calorie mimetic supplement	NCT04962464	Aging
		Diet, sleep, exercise, relaxation guidance, etc.	NCT03472820	Epigenetic age
	/	Nutritional guidance, exercise, cognitive training, etc.	NCT01041989	Cognitive impairment, dementia, and Alzheimer's disease
		Exercise	NCT05232968	Aging
		Distinct lifestyle Interventions	NCT01530724	Nonalcoholic fatty liver disease
DNA methyltransferases	DNA methyltransferase inhibitors	Metformin	NCT04375657	Immunosenescence
		Hydralazine	NCT00000620	Type 2 diabetes mellitus
		Azacytidine	NCT03873311	Myeloid malignancy in older patients
Potential RNA methylation	METTL3 inhibitor	STC‐15	NCT05584111	Cancer
Histone modifications	histone deacetylase inhibitors	Sodium phenylbutyrate	NCT00533559	Diabetes
		Ricolinostat	NCT03176472	Painful diabetic peripheral neuropathy
	HAT inhibitors	Curcumin	NCT01968564	Vascular aging
	Sirtuin‐activating compounds	Resveratrol	NCT05981053	Aging
		Resveratrol	NCT01677611	Type 2 diabetes mellitus
		Berberine	NCT02861261	Type 2 diabetes mellitus
		Berberine	NCT00633282	Nonalcoholic fatty liver disease
Metabolic intervention	AMPK	Metformin	NCT04098666	Mild cognitive impairment and Alzheimer's disease
		Metformin	NCT02432287	Aging
		Metformin	NCT01965756	Alzheimer's disease
	PPARα/γ	Saroglitazar	NCT03061721	Nonalcoholic fatty liver disease
		Elafibranor	NCT01694849	Nonalcoholic fatty liver disease
	SGLT2 inhibitor	Dapagliflozin	NCT03030235	Heart failure with preserved ejection fraction
	GLP‐1	Liraglutide (GLP‐1 analogues)	NCT01237119	Nonalcoholic fatty liver disease
Stem cell and gene therapy	/	hESC‐derived RPE transplantation	NCT01345006, NCT01344993	Age‐related macular degeneration
		AAV–hTERT	NCT04133649	Aging
		AAVrh.10h–APOE ε2 (LX1001)	NCT03634007	Alzheimer's disease
		Adipose tissue‐derived MSCs (AstroStem)	NCT03117738	Alzheimer's disease
		Adipose tissue‐derived MSCs (AlloJoin)	NCT04208646	Osteoarthritis

*Data sources*: ClinicalTrials.gov

### 6.3 Ethical and Societal Implications

With the advancement of epigenetic aging research, ethical considerations and social impacts related to epigenetic reprogramming and genome modification have become increasingly prominent. First, epigenetic research involves a large amount of highly sensitive personal health and lifestyle information. If leaked, this information could cause serious harm to individuals. Moreover, gene editing, compound treatments, individual lifestyle factors, and environmental factors can all cause epigenetic changes. For example, the same stress response pathway (such as oxidative stress) can be activated by different substances like pesticides and nicotine, and there is a lack of specific biomarkers. This complexity makes it difficult to clearly define disease liability and also makes it challenging for research participants to fully understand the implications and potential impacts. Second, the ability to modify the epigenome raises questions about the long‐term effects of these modifications on individuals and their offspring. For instance, if epigenetic changes can be transmitted to future generations, they may have transgenerational effects on aging and disease susceptibility. This could also lead to the creation of “designer babies,” raising a host of complex ethical and moral issues [438]. Finally, research and interventions in epigenetics will prompt policymakers to pay more attention to the relationship between the environment and health and to strengthen measures such as environmental pollution control and public health improvement to reduce disease risks. It will also promote public awareness of the impact of lifestyle and environmental factors on health and foster interdisciplinary collaboration to address complex health problems. However, at the same time, it may also lead to a further widening of health disparities and exacerbate social inequality. Therefore, the rapid development of epigenetics and its therapies brings new hope for human health but also brings complex ethical and social issues. Issues such as privacy protection, misuse of gene editing, and social inequality need to be given sufficient attention alongside technological development. It is essential to develop reasonable policies and regulations to promote the positive role of epigenetics in human health while minimizing its potential negative impacts.

### 6.4 Emerging Frontiers and Future Research Trajectories

One of the most important advances in epigenetic aging research has been the development of high‐resolution mapping techniques [439]. These methods have allowed researchers to identify subtle age‐related changes in the epigenome with unprecedented accuracy. The integration of single‐cell multiomics has provided further insights into how aging affects gene expression at the cellular level, allowing for a better understanding of the molecular underpinnings of aging and related diseases [440].

#### 6.4.1 High‐Resolution Epigenome Mapping Techniques

Whole‐genome bisulfite sequencing (WGBS) is one of the most commonly used techniques for analyzing DNA methylation patterns across the entire genome. WGBS has revealed crucial insights into how DNA methylation changes with age, particularly in genes related to tumor suppression and immune function [9, 11, 441]. ATAC‐seq (Assay for Transposase‐Accessible Chromatin using sequencing) is another powerful method for studying chromatin accessibility. This technique has provided new information on how chromatin remodeling during aging may contribute to age‐related diseases like neurodegeneration and cancer [442, 443].

Moreover, the rise of single‐cell RNA sequencing and single‐cell ATAC‐seq has allowed for the exploration of epigenetic heterogeneity in different cell populations. These techniques are particularly useful for understanding how aging impacts specific cell types, such as stem cells or immune cells, in different tissues [444, 445].

#### 6.4.2 Multiomics Approaches to Aging

Integrating data from various omics fields—epigenomics, transcriptomics, and proteomics—have provided a more holistic view of the aging process. For example, epigenetic clocks, which estimate biological age based on DNA methylation patterns, have been shown to correlate with age‐related diseases and mortality risk. Recent innovations have combined these clocks with other omics data to refine their predictive power, enabling more accurate assessments of an individual's biological age [446, 447, 448, 449]. These approaches are also paving the way for the identification of biomarkers that could be used for early diagnosis of aging‐related diseases and tailored therapies to delay aging or mitigate disease progression [448, 450].

#### 6.4.3 Development of More Precise Epigenetic Editing Tools

To overcome the challenges of target specificity and off‐target effects, future research must focus on improving the precision of epigenetic editing tools. The CRISPR/Cas9‐based epigenetic editing systems have shown significant promise, but there is still a need to refine these technologies for increased specificity and reduced off‐target effects. Researchers are exploring various strategies to improve the precision of these tools, such as using more specific guide RNAs, optimizing dCas9 fusion proteins, and incorporating additional layers of regulation, such as inducible systems that allow for temporal control over epigenetic modifications [451, 452].

In addition to CRISPR‐based tools, small molecule inhibitors and RNA‐based therapies are being investigated to enhance the specificity and efficiency of epigenetic modifications. Nanotechnology and targeted drug delivery systems will also play a crucial role in achieving tissue‐specific delivery and minimizing off‐target effects [453]. Efforts to improve the resolution of epigenetic editing at the single‐cell level will also be essential for understanding the dynamic nature of the epigenome and tailoring therapies to specific cell types and tissues [454].

#### 6.4.4 Exploring Combinatorial Therapies that Target Multiple Aging Pathways

Aging is a multifactorial process that involves the interplay of genetic, epigenetic, environmental, and lifestyle factors. To effectively combat aging and age‐related diseases, future therapies will likely need to target multiple pathways simultaneously. Researchers are increasingly exploring combinatorial approaches that combine epigenetic modifications with other therapeutic modalities, such as senolytics, antioxidants, and immune modulators [455]. These combinatorial therapies could enhance the overall effectiveness of treatment by addressing the multiple drivers of aging, including cellular senescence, oxidative stress, and chronic inflammation.

For example, combining epigenetic reprogramming with senolytic agents that selectively eliminate senescent cells could help rejuvenate tissues by both restoring youthful gene expression and eliminating dysfunctional cells. Similarly, epigenetic therapies that target inflammation‐associated pathways could work synergistically with anti‐inflammatory agents to reduce the chronic low‐grade inflammation that is a hallmark of aging and contributes to many age‐related diseases [456].

#### 6.4.5 Personalized Epigenetic Interventions Based on Aging Profiles

As aging is influenced by a combination of genetic, environmental, and lifestyle factors, personalized epigenetic interventions represent a promising future direction. By using epigenetic clocks—biomarkers of biological age based on DNA methylation patterns—researchers can design individualized therapies tailored to an individual's specific aging profile. Personalized therapies could include lifestyle modifications (e.g., diet, exercise) combined with epigenetic drugs that target the molecular underpinnings of the individual's aging process [457].

Moreover, personalized interventions could focus on specific tissues or organs that are experiencing accelerated aging. For example, epigenetic therapies could be tailored to target age‐related changes in neural tissue, such as those associated with AD, or in cardiovascular tissues, which undergo epigenetic changes that contribute to atherosclerosis and heart disease [458, 459].

## 7 Conclusion

In this review, we underscore the central role of epigenetics in aging. We systematically synthesize evidence accrued over the past decade to establish that epigenetic dysregulation is not only a core driver of aging but also a reversible lever for intervention. Age‐associated alterations spanning DNA methylation, histone modifications, three‐dimensional chromatin architecture, and ncRNA networks have emerged as a unifying mechanism linking genomic instability, transcriptional noise, stem‐cell exhaustion, mitochondrial decline, and major chronic diseases of old age. Because the aging phenotype is shaped by genetics, epigenetics, and environment, we further examine the interplay between epigenetic marks and nongenetic factors in hereditary or metabolic disorders. Unlike genetic mutations, most epigenetic changes are reversible; consequently, epigenetics holds exceptional clinical potential. Several epigenetic biomarkers have been identified, and a growing arsenal of epigenetic drugs has entered development—whose clinical trials we summarize.

Recent studies reveal that aging is not linear but accelerates exponentially with accumulating epigenetic “noise.” DNA‐methylation clocks, histone‐modification clocks, and multiomic integrative clocks predict biological age with a mean absolute error ≤3.5 years and correlate strongly with all‐cause mortality, cardiovascular events, and cognitive decline. CRISPR–dCas9‐mediated writing of H3K9me3 or DNA methylation can reverse cellular senescence markers, while in animal models partial OSKM reprogramming, NAD⁺ precursor supplementation, HDACis, or young‐plasma exosomes markedly reduce epigenetic age and restore function in liver, muscle, and brain. Collectively, these findings position epigenetic reprogramming as a promising route to reverse aging.

Compared with traditional single‐disease treatments, epigenome‐targeted strategies can simultaneously modulate multiple aging pathways, offering the potential for a holistic rejuvenation. Multiomic clocks coupled with single‐cell atlases will enable clinicians to monitor organ‐specific aging trajectories in real time, facilitating precision decisions on when, where, and for how long to intervene. Rational combinations of epigenetic drugs with mitochondrial enhancers, senolytics, and immune modulators could, in theory, reset functional networks at the cellular, tissue, and systemic levels in concert.

Yet significant challenges cloud translation. Off‐target epigenetic editing or systemic HDAC inhibition may reactivate oncogenes or silence tumor suppressors; tissue‐specific delivery and temporal control remain nascent. Partial reprogramming extends lifespan but carries risks of teratoma formation, immune rejection, or malignant transformation. Moreover, issues of social equity, data privacy, and transgenerational epigenetic manipulation demand proactive governance.

## Author Contributions

Yongpan An and Qian Wang: Writing—original draft, writing—review and editing, conceptualization, investigation, and methodology. Ke Gao, Chi Zhang, Yanan Ouyang, Ruixiao Li, Zhou Ma, Tong Wu, and Lifan Zhou: Data curation, software, and Visualization. Zhengwei Xie: Conceptualization, investigation, and methodology. Rui Zhang: Conceptualization, investigation, and methodology. Guojun Wu: Conceptualization, investigation, and methodology. All authors have read and approved the final manuscript.

## Ethics Statement

The authors have nothing to report.

## Conflicts of Interest

The authors declare no conflicts of interest.

## Data Availability

The authors have nothing to report.

## References

[1] Y. Cai , W. Song , J. Li , et al., “The Landscape of Aging,” Sci China Life Sci 65 (2022): 2354–2454. 36066811 10.1007/s11427-022-2161-3 PMC9446657

[2] A. Scott , M. Ellison , and D. Sinclair , “The Economic Value of Targeting Aging,” Nat Aging 1 (2021): 616–623. 37117804 10.1038/s43587-021-00080-0 PMC10154220

[3] C. Lopez‐Otin , M. Blasco , L. Partridge , M. Serrano , and G. Kroemer , “Hallmarks of Aging: An Expanding Universe,” Cell 186 (2023): 243–278. 36599349 10.1016/j.cell.2022.11.001

[4] J. Yang , M. Hayano , P. Griffin , et al., “Loss of Epigenetic Information as a Cause of Mammalian Aging,” Cell 186 (2023): 305–326. 36638792 10.1016/j.cell.2022.12.027 PMC10166133

[5] T. Eisenberg , H. Knauer , A. Schauer , et al., “Induction of Autophagy by Spermidine Promotes Longevity,” Nature Cell Biology 11 (2009): 1305–1314. 19801973 10.1038/ncb1975

[6] W. Zhang , J. Qu , G. Liu , and J. Belmonte , “The Ageing Epigenome and Its Rejuvenation,” Nature Reviews Molecular Cell Biology 21 (2020): 137–150. 32020082 10.1038/s41580-019-0204-5

[7] A. Parkhitko , E. Filine , and M. Tatar , “Combinatorial Interventions in Aging,” Nat Aging 3 (2023): 1187–1200. 37783817 10.1038/s43587-023-00489-9 PMC11194689

[8] S. Fadul , A. Arshad , and R. Mehmood , “CRISPR‐based Epigenome Editing: Mechanisms and Applications,” Epigenomics 15 (2023): 1137–1155. 37990877 10.2217/epi-2023-0281

[9] K. Seale , S. Horvath , A. Teschendorff , N. Eynon , and S. Voisin , “Making Sense of the Ageing Methylome,” Nature Reviews Genetics 23 (2022): 585–605. 10.1038/s41576-022-00477-6 35501397

[10] S. Smith , B. Kaplan , L. Sowers , and E. Newman , “Mechanism of human Methyl‐directed DNA Methyltransferase and the Fidelity of Cytosine Methylation,” PNAS 89 (1992): 4744–4748. 1584813 10.1073/pnas.89.10.4744 PMC49160

[11] X. Ming , Z. Zhang , Z. Zou , et al., “Kinetics and Mechanisms of Mitotic Inheritance of DNA Methylation and Their Roles in Aging‐associated Methylome Deterioration,” Cell Research 30 (2020): 980–996. 32581343 10.1038/s41422-020-0359-9 PMC7785024

[12] S. Johnstone , V. Gladyshev , M. Aryee , and B. Bernstein , “Epigenetic Clocks, Aging, and Cancer,” Science 378 (2022): 1276–1277. 36548410 10.1126/science.abn4009

[13] L. Chouliaras , D. van den Hove , G. Kenis , et al., “Prevention of Age‐related Changes in Hippocampal Levels of 5‐methylcytidine by Caloric Restriction,” Neurobiology of Aging 33 (2012): 1672–1681. 21764481 10.1016/j.neurobiolaging.2011.06.003 PMC3355211

[14] A. Unnikrishnan , W. Freeman , J. Jackson , J. Wren , H. Porter , and A. Richardson , “The Role of DNA Methylation in Epigenetics of Aging,” Pharmacology & Therapeutics 195 (2019): 172–185. 30419258 10.1016/j.pharmthera.2018.11.001 PMC6397707

[15] A. Bick , J. Pirruccello , G. Griffin , et al., “Genetic Interleukin 6 Signaling Deficiency Attenuates Cardiovascular Risk in Clonal Hematopoiesis,” Circulation 141 (2020): 124–131. 31707836 10.1161/CIRCULATIONAHA.119.044362 PMC7008855

[16] C. Chen and H. Huang , “Methyltransferase 3‐mediated m6A Modification of Switch/Sucrose Non‐fermenting‐related Matrix‐associated Actin‐dependent Regulator of Chromatin Subfamily a Member 5 Promotes Mycobacterium Tuberculosis‐infected Macrophage M1 Polarization and Inflammation,” Cytojournal 22 (2025): 38. 40469707 10.25259/Cytojournal_144_2024 PMC12134857

[17] Z. Zhang , R. Lu , P. Wang , et al., “Structural Basis for DNMT3A‐mediated De Novo DNA Methylation,” Nature 554 (2018): 387–391. 29414941 10.1038/nature25477 PMC5814352

[18] M. Yagi , M. Kabata , A. Tanaka , et al., “Identification of Distinct Loci for De Novo DNA Methylation by DNMT3A and DNMT3B During Mammalian Development,” Nature Communications 11 (2020): 3199. 10.1038/s41467-020-16989-w PMC7314859 32581223

[19] A. Lu , Z. Fei , A. Haghani , et al., “Universal DNA Methylation Age Across Mammalian Tissues,” Nat Aging 3 (2023): 1144–1166. 37563227 10.1038/s43587-023-00462-6 PMC10501909

[20] B. Bhargavan , B. Chhunchha , E. Kubo , and D. Singh , “DNA Methylation as an Epigenetic Mechanism in the Regulation of LEDGF Expression and Biological Response in Aging and Oxidative Stress,” Cell Death Discov 10 (2024): 296. 38909054 10.1038/s41420-024-02076-2 PMC11193803

[21] A. So , J. Jung , S. Lee , H. Kim , and K. Kang , “DNA Methyltransferase Controls Stem Cell Aging by Regulating BMI1 and EZH2 Through microRNAs,” PLoS ONE 6 (2011): e19503. 21572997 10.1371/journal.pone.0019503 PMC3091856

[22] K. Wang , H. Liu , Q. Hu , et al., “Epigenetic Regulation of Aging: Implications for Interventions of Aging and Diseases,” Signal Transduct Target Ther 7 (2022): 374. 36336680 10.1038/s41392-022-01211-8 PMC9637765

[23] I. Shchukina , J. Bagaitkar , O. Shpynov , et al., “Enhanced Epigenetic Profiling of Classical human Monocytes Reveals a Specific Signature of Healthy Aging in the DNA Methylome,” Nat Aging 1 (2021): 124–141. 34796338 10.1038/s43587-020-00002-6 PMC8597198

[24] S. Glaser , R. Wagener , H. Kretzmer , et al., “Subtyping Burkitt Lymphoma by DNA Methylation,” Genes, chromosomes & cancer 64 (2025): e70042. 40192513 10.1002/gcc.70042 PMC11974478

[25] N. Song , E. Ji , J. Yu , et al., “Spermidine Enhances Mitochondrial Function and Mitigates Aortic Valve Calcification: Implications for DNA Methyltransferase‐1 Activity,” JACC Basic Transl Sci 10 (2025): 345–366. 40139876 10.1016/j.jacbts.2024.11.011 PMC12013848

[26] J. Xiong , F. Ma , N. Ding , et al., “p Alleviates Homocysteine‐mediated Atherosclerosis by Targeting IL‐31 Through Its Epigenetics Modifications,” Aging Cell 20 (2021): e13485. 34592792 10.1111/acel.13485 PMC8520716

[27] L. Wang , L. Lankhorst , and R. Bernards , “Exploiting Senescence for the Treatment of Cancer,” Nature Reviews Cancer 22 (2022): 340–355. 35241831 10.1038/s41568-022-00450-9

[28] K. Yan , Q. Ji , D. Zhao , et al., “SGF29 nuclear Condensates Reinforce Cellular Aging,” Cell Discovery 9 (2023): 110. 37935676 10.1038/s41421-023-00602-7 PMC10630320

[29] B. Zhang , Q. Long , S. Wu , et al., “KDM4 Orchestrates Epigenomic Remodeling of Senescent Cells and Potentiates the Senescence‐Associated Secretory Phenotype,” Nat Aging 1 (2021): 454–472. 34263179 10.1038/s43587-021-00063-1 PMC8277122

[30] W. Wang , Y. Zheng , S. Sun , et al., “A Genome‐wide CRISPR‐based Screen Identifies KAT7 as a Driver of Cellular Senescence,” Science Translational Medicine 13 (2021): eabd2655. 33408182 10.1126/scitranslmed.abd2655

[31] F. Della Valle , P. Reddy , M. Yamamoto , et al., “LINE‐1 RNA Causes Heterochromatin Erosion and Is a Target for Amelioration of Senescent Phenotypes in Progeroid Syndromes,” Science Translational Medicine 14 (2022): eabl6057. 35947677 10.1126/scitranslmed.abl6057

[32] Z. Liu , Q. Ji , J. Ren , et al., “Large‐scale Chromatin Reorganization Reactivates Placenta‐specific Genes That Drive Cellular Aging,” Developmental Cell 57 (2022): 1347–1368. e1312. 35613614 10.1016/j.devcel.2022.05.004

[33] V. Gorgoulis , P. Adams , A. Alimonti , et al., “Cellular Senescence: Defining a Path Forward,” Cell 179 (2019): 813–827. 31675495 10.1016/j.cell.2019.10.005

[34] A. Trapp , C. Kerepesi , and V. Gladyshev , “Profiling Epigenetic Age in Single Cells,” Nat Aging 1 (2021): 1189–1201. 36211119 10.1038/s43587-021-00134-3 PMC9536112

[35] K. Rudolph , “DNA‐methylation Aging at Single‐cell Level,” Nat Aging 1 (2021): 1086–1087. 37117522 10.1038/s43587-021-00154-z

[36] K. Margiotti , F. Monaco , M. Fabiani , A. Mesoraca , and C. Giorlandino , “Epigenetic Clocks: In Aging‐Related and Complex Diseases,” Cytogenetic and Genome Research 163 (2023): 247–256. 37899027 10.1159/000534561

[37] C. Vatier and S. Christin‐Maitre , “Epigenetic/Circadian Clocks and PCOS,” Human Reproduction 39 (2024): 1167–1175. 38600622 10.1093/humrep/deae066

[38] Z. Wu , J. Qu , W. Zhang , and G. Liu , “Stress, Epigenetics, and Aging: Unraveling the Intricate Crosstalk,” Molecular Cell 84 (2024): 34–54. 37963471 10.1016/j.molcel.2023.10.006

[39] R. Duan , Q. Fu , Y. Sun , and Q. Li , “Epigenetic Clock: A Promising Biomarker and Practical Tool in Aging,” Ageing Research Reviews 81 (2022): 101743. 36206857 10.1016/j.arr.2022.101743

[40] S. Horvath , “DNA Methylation Age of human Tissues and Cell Types,” Genome biology 14 (2013): R115. 24138928 10.1186/gb-2013-14-10-r115 PMC4015143

[41] G. Hannum , J. Guinney , L. Zhao , et al., “Genome‐wide Methylation Profiles Reveal Quantitative Views of human Aging Rates,” Molecular Cell 49 (2013): 359–367. 23177740 10.1016/j.molcel.2012.10.016 PMC3780611

[42] M. Levine , A. Lu , A. Quach , et al., “An Epigenetic Biomarker of Aging for Lifespan and Healthspan,” Aging (Albany NY) 10 (2018): 573–591. 29676998 10.18632/aging.101414 PMC5940111

[43] A. Lu , A. Quach , J. Wilson , et al., “DNA Methylation GrimAge Strongly Predicts Lifespan and Healthspan,” Aging (Albany NY) 11 (2019): 303–327. 30669119 10.18632/aging.101684 PMC6366976

[44] D. Belsky , A. Caspi , D. Corcoran , et al., “DunedinPACE, a DNA Methylation Biomarker of the Pace of Aging,” Elife 11 (2022): e73420. 35029144 10.7554/eLife.73420 PMC8853656

[45] T. Yang , Y. Xiao , Y. Cheng , et al., “Epigenetic Clocks in Neurodegenerative Diseases: A Systematic Review,” Journal of Neurology, Neurosurgery, and Psychiatry 94 (2023): 1064–1070. 36963821 10.1136/jnnp-2022-330931

[46] K. Murach , A. Dimet‐Wiley , Y. Wen , et al., “Late‐life Exercise Mitigates Skeletal Muscle Epigenetic Aging,” Aging Cell 21 (2022): e13527. 34932867 10.1111/acel.13527 PMC8761012

[47] W. Zhao , S. Yu , Y. Xu , et al., “Sleep Traits Causally Affect Epigenetic Age Acceleration: A Mendelian Randomization Study,” Scientific Reports 15 (2025): 7439. 40032851 10.1038/s41598-024-84957-1 PMC11876307

[48] T. Perlstein , J. Jung , A. Wagner , et al., “Alcohol and Aging: Next‐generation Epigenetic Clocks Predict Biological Age Acceleration in Individuals With Alcohol Use Disorder,” Alcohol Clin Exp Res (Hoboken) 49 (2025): 829–842. 40151157 10.1111/acer.70020 PMC12012873

[49] H. Sung and W. Lin , “Causal Effects of Cardiovascular Health on Five Epigenetic Clocks,” Clin Epigenetics 16 (2024): 134. 39334501 10.1186/s13148-024-01752-5 PMC11438310

[50] Y. Lo and W. Lin , “Cardiovascular Health and Four Epigenetic Clocks,” Clin Epigenetics 14 (2022): 73. 35681159 10.1186/s13148-022-01295-7 PMC9185918

[51] A. Bienkowska , G. Raddatz , J. Sohle , et al., “Development of an Epigenetic Clock to Predict Visual Age Progression of human Skin,” Front Aging 4 (2023): 1258183. 38274286 10.3389/fragi.2023.1258183 PMC10809641

[52] D. Kim , J. Kang , J. Kim , et al., “Assessing the Utility of Epigenetic Clocks for Health Prediction in South Korean,” Front Aging 5 (2024): 1493406. 39687863 10.3389/fragi.2024.1493406 PMC11646986

[53] J. Peng , C. Yuan , X. Hua , and Z. Zhang , “Molecular Mechanism of Histone Variant H2A.B on Stability and Assembly of Nucleosome and Chromatin Structures,” Epigenetics & chromatin 13 (2020): 28. 32664941 10.1186/s13072-020-00351-x PMC7362417

[54] B. Schumacher , J. Pothof , J. Vijg , and J. Hoeijmakers , “The central Role of DNA Damage in the Ageing Process,” Nature 592 (2021): 695–703. 33911272 10.1038/s41586-021-03307-7 PMC9844150

[55] P. Mustafi , M. Hu , S. Kumari , C. Das , G. Li , and T. Kundu , “Phosphorylation‐dependent Association of human Chromatin Protein PC4 to Linker Histone H1 Regulates Genome Organization and Transcription,” Nucleic Acids Res. 50 (2022): 6116–6136. 35670677 10.1093/nar/gkac450 PMC9226532

[56] Y. Lu , X. Tian , and D. Sinclair , “The Information Theory of Aging,” Nat Aging 3 (2023): 1486–1499. 38102202 10.1038/s43587-023-00527-6

[57] G. Millan‐Zambrano , A. Burton , A. Bannister , and R. Schneider , “Histone Post‐translational Modifications—cause and Consequence of Genome Function,” Nature Reviews Genetics 23 (2022): 563–580. 10.1038/s41576-022-00468-7 35338361

[58] A. Patel , Y. He , and I. Radhakrishnan , “Histone Acetylation and Deacetylation—Mechanistic Insights From Structural Biology,” Gene 890 (2024): 147798. 37726026 10.1016/j.gene.2023.147798 PMC11253779

[59] R. Liu , J. Wu , H. Guo , et al., “Post‐translational Modifications of Histones: Mechanisms, Biological Functions, and Therapeutic Targets,” MedComm 2023 (2020): e292. 10.1002/mco2.292 PMC10200003 37220590

[60] K. Bonitto , K. Sarathy , K. Atai , M. Mitra , and H. Coller , “Is There a Histone Code for Cellular Quiescence?” Frontiers in Cell and Developmental Biology 9 (2021): 739780. 34778253 10.3389/fcell.2021.739780 PMC8586460

[61] N. Fuggle , F. Laskou , N. Harvey , and E. Dennison , “A Review of Epigenetics and Its Association With Ageing of Muscle and Bone,” Maturitas 165 (2022): 12–17. 35841774 10.1016/j.maturitas.2022.06.014

[62] S. Mishra , M. Raval , A. Kachhawaha , B. Tiwari , and A. Tiwari , “Aging: Epigenetic Modifications,” Progress in Molecular Biology and Translational Science 197 (2023): 171–209. 37019592 10.1016/bs.pmbts.2023.02.002

[63] C. Soto‐Palma , L. Niedernhofer , C. Faulk , and X. Dong , “Epigenetics, DNA Damage, and Aging,” Journal of Clinical Investigation 132 (2022): e158446. 35968782 10.1172/JCI158446 PMC9374376

[64] Y. Zhang , Z. Sun , J. Jia , et al., “Overview of Histone Modification,” Advances in Experimental Medicine and Biology 1283 (2021): 1–16. 33155134 10.1007/978-981-15-8104-5_1

[65] A. Zhao , W. Xu , R. Han , et al., “Role of Histone Modifications in Neurogenesis and Neurodegenerative Disease Development,” Ageing Research Reviews 98 (2024): 102324. 38762100 10.1016/j.arr.2024.102324

[66] K. Weaver , R. Holt , E. Henry , Y. Lyu , and S. Pletcher , “Effects of Hunger on Neuronal Histone Modifications Slow Aging in Drosophila,” Science 380 (2023): 625–632. 37167393 10.1126/science.ade1662 PMC11837410

[67] Y. Sun , H. Zhang , T. Qiu , L. Liao , and X. Su , “Epigenetic Regulation of Mesenchymal Stem Cell Aging Through Histone Modifications,” Genes Dis 10 (2023): 2443–2456. 37554203 10.1016/j.gendis.2022.10.030 PMC10404871

[68] A. la Torre , F. Lo Vecchio , and A. Greco , “Epigenetic Mechanisms of Aging and Aging‐Associated Diseases,” Cells 12 (2023): 1163. 37190071 10.3390/cells12081163 PMC10136616

[69] M. Al Ojaimi , B. Banimortada , A. Othman , K. Riedhammer , M. Almannai , and A. El‐Hattab , “Disorders of Histone Methylation: Molecular Basis and Clinical Syndromes,” Clinical Genetics 102 (2022): 169–181. 35713103 10.1111/cge.14181

[70] D. Sun , M. Luo , M. Jeong , et al., “Epigenomic Profiling of Young and Aged HSCs Reveals Concerted Changes During Aging That Reinforce Self‐renewal,” Cell Stem Cell 14 (2014): 673–688. 24792119 10.1016/j.stem.2014.03.002 PMC4070311

[71] N. Itokawa , M. Oshima , S. Koide , et al., “Epigenetic Traits Inscribed in Chromatin Accessibility in Aged Hematopoietic Stem Cells,” Nature Communications 13 (2022): 2691. 10.1038/s41467-022-30440-2 PMC9110722 35577813

[72] B. Benayoun , E. Pollina , P. Singh , et al., “Remodeling of Epigenome and Transcriptome Landscapes With Aging in Mice Reveals Widespread Induction of Inflammatory Responses,” Genome Research 29 (2019): 697–709. 30858345 10.1101/gr.240093.118 PMC6442391

[73] L. Liu , T. Cheung , G. Charville , et al., “Chromatin Modifications as Determinants of Muscle Stem Cell Quiescence and Chronological Aging,” Cell reports 4 (2013): 189–204. 23810552 10.1016/j.celrep.2013.05.043 PMC4103025

[74] P. Sen , P. Shah , R. Nativio , and S. Berger , “Epigenetic Mechanisms of Longevity and Aging,” Cell 166 (2016): 822–839. 27518561 10.1016/j.cell.2016.07.050 PMC5821249

[75] P. Cheung , F. Vallania , H. Warsinske , et al., “Single‐Cell Chromatin Modification Profiling Reveals Increased Epigenetic Variations With Aging,” Cell 173 (2018): 1385–1397. e1314. 29706550 10.1016/j.cell.2018.03.079 PMC5984186

[76] J. Li , J. Li , and E. Guo , “Quercetin Suppresses Glioma Stem Cells via Activating p16‐INK4 Gene Expression Through Epigenetic Regulation,” Anti‐cancer agents in medicinal chemistry 25, no. 14 (2025): 1041–1048, 10.2174/0118715206332048241126095207. 39901683

[77] Y. Li , G. Fang , W. Cao , et al., “Ezh2 Inhibits Replicative Senescence of Atrial Fibroblasts through Promotion of H3K27me3 in the Promoter Regions of CDKN2a and Timp4 Genes,” J Inflamm Res 15 (2022): 4693–4708. 35996686 10.2147/JIR.S374951 PMC9392478

[78] T. Yasuda , M. Koiwa , A. Yonemura , et al., “Inflammation‐driven Senescence‐associated Secretory Phenotype in Cancer‐associated Fibroblasts Enhances Peritoneal Dissemination,” Cell reports 34 (2021): 108779. 33626356 10.1016/j.celrep.2021.108779

[79] S. Sugita , T. Aoyama , M. Emori , et al., “Assessment of H3K27me3 Immunohistochemistry and Combination of NF1 and p16 Deletions by Fluorescence in Situ Hybridization in the Differential Diagnosis of Malignant Peripheral Nerve Sheath Tumor and Its Histological Mimics,” Diagn Pathol 16 (2021): 79. 34461930 10.1186/s13000-021-01140-0 PMC8404283

[80] H. Zhang , J. Li , Y. Yu , et al., “Nuclear Lamina Erosion‐induced Resurrection of Endogenous Retroviruses Underlies Neuronal Aging,” Cell reports 42 (2023): 113396. 37910499 10.1016/j.celrep.2023.113396

[81] F. Xiao , H. Wang , X. Chen , et al., “Hypermethylation in H3K9me3 Regions Characterizes the Centenarian Methylomes in Healthy Aging,” National Science Review 10 (2023): nwad067. 37181094 10.1093/nsr/nwad067 PMC10171629

[82] Q. Wan , X. Meng , C. Wang , et al., “Histone H3K4me3 Modification Is a Transgenerational Epigenetic Signal for Lipid Metabolism in Caenorhabditis elegans,” Nature Communications 13 (2022): 768. 10.1038/s41467-022-28469-4 PMC8828817 35140229

[83] Y. Guo , S. Zhao , and G. Wang , “Polycomb Gene Silencing Mechanisms: PRC2 Chromatin Targeting, H3K27me3 'Readout', and Phase Separation‐Based Compaction,” Trends in Genetics 37 (2021): 547–565. 33494958 10.1016/j.tig.2020.12.006 PMC8119337

[84] S. Ro , “Improving Gastric Motility in Aging through EZH2 Inhibition and Preservation of Interstitial Cells of Cajal,” Cell Mol Gastroenterol Hepatol 18 (2024): 101382. 39127454 10.1016/j.jcmgh.2024.101382 PMC11519705

[85] R. Kumbhar , A. Sanchez , J. Perren , et al., “Poly(ADP‐ribose) Binding and macroH2A Mediate Recruitment and Functions of KDM5A at DNA Lesions,” Journal of Cell Biology 220 (2021): e202006149. 34003252 10.1083/jcb.202006149 PMC8135068

[86] A. Godbole , S. Gopalan , T. Nguyen , et al., “S‐adenosylmethionine Synthases Specify Distinct H3K4me3 Populations and Gene Expression Patterns During Heat Stress,” Elife 12 (2023): e79511. 36756948 10.7554/eLife.79511 PMC9984191

[87] Y. Xiao , F. Liu , Q. Kong , et al., “Metformin Induces S‐adenosylmethionine Restriction to Extend the Caenorhabditis elegans Healthspan Through H3K4me3 Modifiers,” Aging Cell 21 (2022): e13567. 35146893 10.1111/acel.13567 PMC8920454

[88] Y. Zhang , R. Ma , Q. Deng , et al., “S‐adenosylmethionine Improves Cognitive Impairment in D‐galactose‐induced Brain Aging by Inhibiting Oxidative Stress and Neuroinflammation,” Journal of Chemical Neuroanatomy 128 (2023): 102232. 36632907 10.1016/j.jchemneu.2023.102232

[89] Z. Gu , Y. Wang , Z. Fang , et al., “Plasma Metabolomics Identifies S‐adenosylmethionine as a Biomarker and Potential Therapeutic Target for Vascular Aging in Older Adult Males,” Journal of Pharmaceutical and Biomedical Analysis 243 (2024): 116097. 38489960 10.1016/j.jpba.2024.116097

[90] S. Okstad , I. Mair , J. Sundsfjord , T. Eide , and I. Nordrum , “[Ectopic thyroid tissue in the head and neck],” Tidsskrift for Den Norske Laegeforening 107 (1987): 249–250. 3824313

[91] T. Chen , F. Wang , P. Lee , A. Hsu , and T. Ching , “Mitochondrial S‐adenosylmethionine Deficiency Induces Mitochondrial Unfolded Protein Response and Extends Lifespan in Caenorhabditis elegans,” Aging Cell 23 (2024): e14103. 38361361 10.1111/acel.14103 PMC11019128

[92] A. Bellver‐Sanchis , Q. Geng , G. Navarro , et al., “G9a Inhibition Promotes Neuroprotection Through GMFB Regulation in Alzheimer's Disease,” Aging Dis 15 (2024): 311–337. 37307824 10.14336/AD.2023.0424-2 PMC10796087

[93] B. Signal , A. Phipps , K. Giles , et al., “Ageing‐Related Changes to H3K4me3, H3K27ac, and H3K27me3 in Purified Mouse Neurons,” Cells 13 (2024): 1393. 39195281 10.3390/cells13161393 PMC11353134

[94] Z. Yang , D. Yu , F. Gao , et al., “The Histone Lysine Demethylase KDM7A Contributes to Reward Memory via Fscn1‐Induced Synaptic Plasticity in the Medial Prefrontal Cortex,” Adv Sci (Weinh) 12 (2025): e2405352. 39836528 10.1002/advs.202405352 PMC11905110

[95] L. Xiao , J. Qiao , Y. Huang , et al., “RASGRP1 targeted by H3K27me3 Regulates Myoblast Proliferation and Differentiation in Mice and Pigs,” Acta Biochim Biophys Sin (Shanghai) 56 (2024): 452–461. 38419500 10.3724/abbs.2024011 PMC10984873

[96] J. Shimizu and F. Kawano , “DNA Hypermethylation Preceded by H3K27 Trimethylation Is Linked to Downregulation of Gene Expression in Disuse Muscle Atrophy in Male Mice,” Physiological Reports 13 (2025): e70317. 40223401 10.14814/phy2.70317 PMC11994892

[97] M. Izadi , N. Sadri , A. Abdi , S. Serajian , D. Jalalei , and S. Tahmasebi , “Epigenetic Biomarkers in Aging and Longevity: Current and Future Application,” Life Sciences 351 (2024): 122842. 38879158 10.1016/j.lfs.2024.122842

[98] Z. Sun , Y. Zhang , J. Jia , et al., “H3K36me3, message From Chromatin to DNA Damage Repair,” Cell Biosci 10 (2020): 9. 32021684 10.1186/s13578-020-0374-z PMC6995143

[99] L. Su , H. Li , C. Huang , et al., “Muscle‐Specific Histone H3K36 Dimethyltransferase SET‐18 Shortens Lifespan of Caenorhabditis elegans by Repressing Daf‐16a Expression,” Cell reports 22 (2018): 2716–2729. 29514099 10.1016/j.celrep.2018.02.029

[100] L. Ni , B. Lin , Y. Zhang , et al., “Histone Modification Landscape and the Key Significance of H3K27me3 in Myocardial Ischaemia/Reperfusion Injury,” Sci China Life Sci 66 (2023): 1264–1279. 36808292 10.1007/s11427-022-2257-9

[101] M. Bouwman , D. de Bakker , H. Honkoop , et al., “Cross‐species Comparison Reveals That Hmga1 Reduces H3K27me3 Levels to Promote Cardiomyocyte Proliferation and Cardiac Regeneration,” Nat Cardiovasc Res 4 (2025): 64–82. 39747457 10.1038/s44161-024-00588-9 PMC11738996

[102] J. Cordero , A. Elsherbiny , Y. Wang , et al., “Leveraging Chromatin state Transitions for the Identification of Regulatory Networks Orchestrating Heart Regeneration,” Nucleic Acids Res. 52 (2024): 4215–4233. 38364861 10.1093/nar/gkae085 PMC11077086

[103] C. Bonfiglio , M. Lacy , V. Triantafyllidou , et al., “Ezh2 Shapes T Cell Plasticity to Drive Atherosclerosis,” Circulation 151, no. 19 (2025): 1391–1408, 10.1161/CIRCULATIONAHA.124.072384. 39917842 PMC12063685

[104] P. Lu , J. Xu , X. Shen , et al., “Spatiotemporal Role of SETD2‐H3K36me3 in Murine Pancreatic Organogenesis,” Cell reports 43 (2024): 113703. 38265933 10.1016/j.celrep.2024.113703

[105] S. Guo , J. Fang , W. Xu , et al., “Interplay Between H3K36me3, Methyltransferase SETD2, and Mismatch Recognition Protein MutSalpha Facilitates Processing of Oxidative DNA Damage in human Cells,” Journal of Biological Chemistry 298 (2022): 102102. 35667440 10.1016/j.jbc.2022.102102 PMC9241034

[106] A. Guerrero , A. Innes , P. Roux , et al., “3‐deazaadenosine (3DA) Alleviates Senescence to Promote Cellular Fitness and Cell Therapy Efficiency in Mice,” Nat Aging 2 (2022): 851–866. 36438588 10.1038/s43587-022-00279-9 PMC7613850

[107] X. Yi , Q. Zhu , X. Wu , T. Tan , and X. Jiang , “Histone Methylation and Oxidative Stress in Cardiovascular Diseases,” Oxid Med Cell Longev 2022 (2022): 6023710. 35340204 10.1155/2022/6023710 PMC8942669

[108] M. Huang , Q. Wu , and Z. Jiang , “Epigenetic Alterations Under Oxidative Stress in Stem Cells,” Oxid Med Cell Longev 2022 (2022): 6439097. 36071870 10.1155/2022/6439097 PMC9444469

[109] X. Zheng and A. Sawalha , “The Role of Oxidative Stress in Epigenetic Changes Underlying Autoimmunity,” Antioxid Redox Signaling 36 (2022): 423–440. 10.1089/ars.2021.0066 PMC8982122 34544258

[110] C. Wang , B. Hu , Y. Yang , et al., “Structural Simulation and Selective Inhibitor Discovery Study for Histone Demethylases KDM4E/6B From a Computational Perspective,” Computational Biology and Chemistry 110 (2024): 108072. 38636391 10.1016/j.compbiolchem.2024.108072

[111] Z. Wu , W. Zhang , J. Qu , and G. Liu , “Emerging Epigenetic Insights Into Aging Mechanisms and Interventions,” Trends in Pharmacological Sciences 45 (2024): 157–172. 38216430 10.1016/j.tips.2023.12.002

[112] J. Xu , C. Li , and X. Kang , “The Epigenetic Regulatory Effect of Histone Acetylation and Deacetylation on Skeletal Muscle Metabolism‐a Review,” Front Physiol 14 (2023): 1267456. 38148899 10.3389/fphys.2023.1267456 PMC10749939

[113] J. Ouyang , D. Wu , Y. Gan , Y. Tang , H. Wang , and J. Huang , “Unraveling the Metabolic‒Epigenetic Nexus: A New Frontier in Cardiovascular Disease Treatment,” Cell death & disease 16 (2025): 183. 40102393 10.1038/s41419-025-07525-z PMC11920384

[114] J. Zhang , F. Chen , Y. Tian , et al., “PARylated PDHE1alpha Generates Acetyl‐CoA for Local Chromatin Acetylation and DNA Damage Repair,” Nature structural & molecular biology 30 (2023): 1719–1734. 10.1038/s41594-023-01107-3 37735618

[115] P. Bradshaw , “Acetyl‐CoA Metabolism and Histone Acetylation in the Regulation of Aging and Lifespan,” Antioxidants (Basel) 10 (2021): 572. 33917812 10.3390/antiox10040572 PMC8068152

[116] S. Serio , C. Pagiatakis , E. Musolino , et al., “Cardiac Aging Is Promoted by Pseudohypoxia Increasing p300‐Induced Glycolysis,” Circulation Research 133 (2023): 687–703. 37681309 10.1161/CIRCRESAHA.123.322676

[117] L. Qiu , X. Liu , H. Xia , and C. Xu , “Downregulation of P300/CBP‐Associated Factor Protects From Vascular Aging via Nrf2 Signal Pathway Activation,” International Journal of Molecular Sciences 23 (2022): 12574. 36293441 10.3390/ijms232012574 PMC9603891

[118] A. Price , M. Manjegowda , J. Kain , S. Anandh , and I. Bochkis , “Hdac3, Setdb1, and Kap1 Mark H3K9me3/H3K14ac Bivalent Regions in Young and Aged Liver,” Aging Cell 19 (2020): e13092. 31858687 10.1111/acel.13092 PMC6996956

[119] T. Pandita , C. Hunt , V. Singh , et al., “Role of the Histone Acetyl Transferase MOF and the Histone Deacetylase Sirtuins in Regulation of H4K16ac during DNA Damage Repair and Metabolic Programming: Implications in Cancer and Aging,” Sub‐Cellular Biochemistry 100 (2022): 115–141. 36301493 10.1007/978-3-031-07634-3_4

[120] Y. You and W. Liang , “SIRT1 and SIRT6: The Role in Aging‐related Diseases,” Biochim Biophys Acta Mol Basis Dis 1869 (2023): 166815. 37499928 10.1016/j.bbadis.2023.166815

[121] J. Chen , A. Wang , and Q. Chen , “SirT3 and p53 Deacetylation in Aging and Cancer,” Journal of Cellular Physiology 232 (2017): 2308–2311. 27791271 10.1002/jcp.25669

[122] Z. Ji , G. Liu , and J. Qu , “Mitochondrial Sirtuins, Metabolism, and Aging,” J Genet Genomics 49 (2022): 287–298. 34856390 10.1016/j.jgg.2021.11.005

[123] Y. Ye , K. Yang , H. Liu , et al., “SIRT2 counteracts Primate Cardiac Aging via Deacetylation of STAT3 That Silences CDKN2B,” Nat Aging 3 (2023): 1269–1287. 37783815 10.1038/s43587-023-00486-y

[124] Y. Ding , T. Wang , S. Lv , et al., “SIRT6 is an Epigenetic Repressor of Thoracic Aortic Aneurysms via Inhibiting Inflammation and Senescence,” Signal Transduct Target Ther 8 (2023): 255. 37394473 10.1038/s41392-023-01456-x PMC10315397

[125] Z. Guo , P. Li , J. Ge , and H. Li , “SIRT6 in Aging, Metabolism, Inflammation and Cardiovascular Diseases,” Aging Dis 13 (2022): 1787–1822. 36465178 10.14336/AD.2022.0413 PMC9662279

[126] O. Lozoya , T. Wang , D. Grenet , et al., “Mitochondrial Acetyl‐CoA Reversibly Regulates Locus‐specific Histone Acetylation and Gene Expression,” Life Sci Alliance 2 (2019): e201800228. 30737248 10.26508/lsa.201800228 PMC6369536

[127] W. Li , Q. Long , H. Wu , et al., “Nuclear Localization of Mitochondrial TCA Cycle Enzymes Modulates Pluripotency via Histone Acetylation,” Nature Communications 13 (2022): 7414. 10.1038/s41467-022-35199-0 PMC9718843 36460681

[128] C. Chen , M. Zhou , Y. Ge , and X. Wang , “SIRT1 and Aging Related Signaling Pathways,” Mechanisms of Ageing and Development 187 (2020): 111215. 32084459 10.1016/j.mad.2020.111215

[129] A. Covarrubias , R. Perrone , A. Grozio , and E. Verdin , “NAD(+) Metabolism and Its Roles in Cellular Processes During Ageing,” Nature Reviews Molecular Cell Biology 22 (2021): 119–141. 33353981 10.1038/s41580-020-00313-x PMC7963035

[130] K. Joshi , S. Liu , S. Breslin , and J. Zhang , “Mechanisms That Regulate the Activities of TET Proteins,” Cellular and Molecular Life Sciences 79 (2022): 363. 35705880 10.1007/s00018-022-04396-x PMC9756640

[131] Y. Feng , L. Cai , W. Hong , et al., “Rewiring of 3D Chromatin Topology Orchestrates Transcriptional Reprogramming and the Development of Human Dilated Cardiomyopathy,” Circulation 145 (2022): 1663–1683. 35400201 10.1161/CIRCULATIONAHA.121.055781 PMC9251830

[132] R. Li and X. Lin , “Connected Chromatin Amplifies Acetylation‐Modulated Nucleosome Interactions,” Biochemistry 64 (2025): 1222–1232. 40029962 10.1021/acs.biochem.4c00647 PMC11925056

[133] C. Dube , F. Jahan , and C. Lim , “Key Changes in Chromatin Mark Mammalian Epidermal Differentiation and Ageing,” Epigenetics 17 (2022): 444–459. 33890553 10.1080/15592294.2021.1917812 PMC8993096

[134] M. Shvedunova and A. Akhtar , “Modulation of Cellular Processes by Histone and Non‐histone Protein Acetylation,” Nature Reviews Molecular Cell Biology 23 (2022): 329–349. 35042977 10.1038/s41580-021-00441-y

[135] Y. Lin , A. Lin , L. Cai , et al., “ACSS2‐dependent Histone Acetylation Improves Cognition in Mouse Model of Alzheimer's disease,” Mol Neurodegener 18 (2023): 47. 37438762 10.1186/s13024-023-00625-4 PMC10339567

[136] D. Dai , J. Xie , Y. Zheng , F. Chen , B. Zhao , and L. Miao , “H3K27 acetylation‐induced FSTL1 Upregulation by P300/RUNX1 co‐activation Exacerbated Autophagy‐mediated Neuronal Damage and NF‐kappaB‐stimulated Inflammation in Alzheimer's disease,” Cytotechnology 75 (2023): 449–460. 37655275 10.1007/s10616-023-00589-9 PMC10465437

[137] S. Marzi , S. Leung , T. Ribarska , et al., “A Histone Acetylome‐wide Association Study of Alzheimer's Disease Identifies Disease‐associated H3K27ac Differences in the Entorhinal Cortex,” Nature Neuroscience 21 (2018): 1618–1627. 30349106 10.1038/s41593-018-0253-7

[138] A. Greissel , M. Culmes , R. Burgkart , et al., “Histone Acetylation and Methylation Significantly Change With Severity of Atherosclerosis in human Carotid Plaques,” Cardiovascular Pathology 25 (2016): 79–86. 26764138 10.1016/j.carpath.2015.11.001

[139] H. Kai , Q. Wu , R. Yin , et al., “LncRNA NORAD Promotes Vascular Endothelial Cell Injury and Atherosclerosis through Suppressing VEGF Gene Transcription via Enhancing H3K9 Deacetylation by Recruiting HDAC6,” Frontiers in Cell and Developmental Biology 9 (2021): 701628. 34307380 10.3389/fcell.2021.701628 PMC8301222

[140] Y. Zhu , L. Shang , Y. Tang , et al., “Genome‐Wide Profiling of H3K27ac Identifies TDO2 as a Pivotal Therapeutic Target in Metabolic Associated Steatohepatitis Liver Disease,” Adv Sci (Weinh) 11 (2024): e2404224. 39364706 10.1002/advs.202404224 PMC11615751

[141] Q. Song , X. Zhou , K. Xu , S. Liu , X. Zhu , and J. Yang , “The Safety and Antiaging Effects of Nicotinamide Mononucleotide in Human Clinical Trials: An Update,” Advances in nutrition 14 (2023): 1416–1435. 37619764 10.1016/j.advnut.2023.08.008 PMC10721522

[142] L. Guarente , D. Sinclair , and G. Kroemer , “Human Trials Exploring Anti‐aging Medicines,” Cell metabolism 36 (2024): 354–376. 38181790 10.1016/j.cmet.2023.12.007

[143] K. Freeberg , C. Udovich , C. Martens , D. Seals , and D. Craighead , “Dietary Supplementation with NAD+‐Boosting Compounds in Humans: Current Knowledge and Future Directions,” Journals of Gerontology. Series A, Biological Sciences and Medical Sciences 78 (2023): 2435–2448. 37068054 10.1093/gerona/glad106 PMC10692436

[144] Y. Qiu , Q. Xu , P. Xie , et al., “Epigenetic Modifications and Emerging Therapeutic Targets in Cardiovascular Aging and Diseases,” Pharmacological Research 211 (2025): 107546. 39674563 10.1016/j.phrs.2024.107546

[145] N. Gasek , G. Kuchel , J. Kirkland , and M. Xu , “Strategies for Targeting Senescent Cells in Human Disease,” Nat Aging 1 (2021): 870–879. 34841261 10.1038/s43587-021-00121-8 PMC8612694

[146] A. Sharma , S. Chabloz , R. Lapides , E. Roider , and C. Ewald , “Potential Synergistic Supplementation of NAD+ Promoting Compounds as a Strategy for Increasing Healthspan,” Nutrients 15 (2023): 445. 36678315 10.3390/nu15020445 PMC9861325

[147] D. Wilson 3rd , M. Cookson , L. Van Den Bosch , H. Zetterberg , D. Holtzman , and , I. Dewachter , “Hallmarks of Neurodegenerative Diseases,” Cell 186 (2023): 693–714. 36803602 10.1016/j.cell.2022.12.032

[148] A. Sharma , K. Singh , and A. Almasan , “Histone H2AX Phosphorylation: A Marker for DNA Damage,” Methods in Molecular Biology 920 (2012): 613–626. 22941631 10.1007/978-1-61779-998-3_40

[149] T. Oizumi , T. Suzuki , J. Kobayashi , and A. Nakamura , “Senescence‐Associated Heterochromatin Foci Suppress Gamma‐H2AX Focus Formation Induced by Radiation Exposure,” International Journal of Molecular Sciences 25 (2024): 3355. 38542327 10.3390/ijms25063355 PMC10969922

[150] T. Liu , J. Lin , C. Chen , et al., “MicroRNA‐146b‐5p Overexpression Attenuates Premature Ovarian Failure in Mice by Inhibiting the Dab2ip/Ask1/p38‐Mapk Pathway and gammaH2A.X Phosphorylation,” Cell Proliferation 54 (2021): e12954. 33166004 10.1111/cpr.12954 PMC7791167

[151] S. Maity , K. Farrell , S. Navabpour , S. Narayanan , and T. Jarome , “Epigenetic Mechanisms in Memory and Cognitive Decline Associated With Aging and Alzheimer's Disease,” International Journal of Molecular Sciences 22 (2021): 12280. 34830163 10.3390/ijms222212280 PMC8618067

[152] A. Kinner , W. Wu , C. Staudt , and G. Iliakis , “Gamma‐H2AX in Recognition and Signaling of DNA Double‐strand Breaks in the Context of Chromatin,” Nucleic Acids Res. 36 (2008): 5678–5694. 18772227 10.1093/nar/gkn550 PMC2553572

[153] A. Georgoulis , C. Vorgias , G. Chrousos , and E. Rogakou , “Genome Instability and gammaH2AX,” International Journal of Molecular Sciences 18 (2017): 1979. 28914798 10.3390/ijms18091979 PMC5618628

[154] C. Lucca , E. Ferrari , G. Shubassi , et al., “Sch9(S6K) controls DNA Repair and DNA Damage Response Efficiency in Aging Cells,” Cell reports 43 (2024): 114281. 38805395 10.1016/j.celrep.2024.114281

[155] S. Wang , X. Zhang , Y. Hou , et al., “SIRT6 activates PPARalpha to Improve Doxorubicin‐induced Myocardial Cell Aging and Damage,” Chemico‐Biological Interactions 392 (2024): 110920. 38395252 10.1016/j.cbi.2024.110920

[156] H. Kim , J. Cho , H. Quan , and J. Kim , “Down‐regulation of Aurora B Kinase Induces Cellular Senescence in human Fibroblasts and Endothelial Cells Through a p53‐dependent Pathway,” Febs Letters 585 (2011): 3569–3576. 22024481 10.1016/j.febslet.2011.10.022

[157] B. Pierce , “The Aging Epigenome,” Elife 11 (2022): e78693. 35481978 10.7554/eLife.78693 PMC9049969

[158] B. Bartholomew , “Regulating the Chromatin Landscape: Structural and Mechanistic Perspectives,” Annual Review of Biochemistry 83 (2014): 671–696. 10.1146/annurev-biochem-051810-093157 PMC4332854 24606138

[159] G. Cavalli and T. Misteli , “Functional Implications of Genome Topology,” Nature structural & molecular biology 20 (2013): 290–299. 10.1038/nsmb.2474 PMC6320674 23463314

[160] B. Morris , B. Willcox , and T. Donlon , “Genetic and Epigenetic Regulation of human Aging and Longevity,” Biochim Biophys Acta Mol Basis Dis 1865 (2019): 1718–1744. 31109447 10.1016/j.bbadis.2018.08.039 PMC7295568

[161] X. Wei , M. Murphy , N. Reddy , et al., “Redistribution of Lamina‐associated Domains Reshapes Binding of Pioneer Factor FOXA2 in Development of Nonalcoholic Fatty Liver Disease,” Genome Research 32 (2022): 1981–1992. 36522168 10.1101/gr.277149.122 PMC9808618

[162] E. Di Giorgio and L. Xodo , “Endogenous Retroviruses (ERVs): Does RLR (RIG‐I‐Like Receptors)‐MAVS Pathway Directly Control Senescence and Aging as a Consequence of ERV De‐Repression?” Frontiers in immunology 13 (2022): 917998. 35757716 10.3389/fimmu.2022.917998 PMC9218063

[163] M. Lachner , D. O'Carroll , S. Rea , K. Mechtler , and T. Jenuwein , “Methylation of Histone H3 Lysine 9 Creates a Binding Site for HP1 Proteins,” Nature 410 (2001): 116–120. 11242053 10.1038/35065132

[164] A. Bannister , P. Zegerman , J. Partridge , et al., “Selective Recognition of Methylated Lysine 9 on Histone H3 by the HP1 Chromo Domain,” Nature 410 (2001): 120–124. 11242054 10.1038/35065138

[165] C. Maison and G. Almouzni , “HP1 and the Dynamics of Heterochromatin Maintenance,” Nature Reviews Molecular Cell Biology 5 (2004): 296–304. 15071554 10.1038/nrm1355

[166] W. Zeng , A. Ball Jr. , and K. Yokomori , “HP1: Heterochromatin Binding Proteins Working the Genome,” Epigenetics 5 (2010): 287–292. 20421743 10.4161/epi.5.4.11683 PMC3103764

[167] J. Padeken , S. Methot , and S. Gasser , “Establishment of H3K9‐methylated Heterochromatin and Its Functions in Tissue Differentiation and Maintenance,” Nature Reviews Molecular Cell Biology 23 (2022): 623–640. 35562425 10.1038/s41580-022-00483-w PMC9099300

[168] R. Li , Y. Teng , Y. Guo , et al., “Aging‐related Decrease of Histone Methyltransferase SUV39H1 in Adipose‐derived Stem Cells Enhanced SASP,” Mechanisms of Ageing and Development 215 (2023): 111868. 37666472 10.1016/j.mad.2023.111868

[169] W. Zhang , J. Li , K. Suzuki , et al., “Aging Stem Cells. A Werner syndrome Stem Cell Model Unveils Heterochromatin Alterations as a Driver of human Aging,” Science 348 (2015): 1160–1163. 25931448 10.1126/science.aaa1356 PMC4494668

[170] Z. Li , S. Duan , X. Hua , et al., “Asymmetric Distribution of Parental H3K9me3 in S Phase Silences L1 Elements,” Nature 623 (2023): 643–651. 37938774 10.1038/s41586-023-06711-3 PMC11034792

[171] G. Andrey and S. Mundlos , “The Three‐dimensional Genome: Regulating Gene Expression During Pluripotency and Development,” Development (Cambridge, England) 144 (2017): 3646–3658. 29042476 10.1242/dev.148304

[172] F. Ma , Y. Cao , H. Du , et al., “Three‐dimensional Chromatin Reorganization Regulates B Cell Development During Ageing,” Nature Cell Biology 26 (2024): 991–1002. 38866970 10.1038/s41556-024-01424-9 PMC11178499

[173] L. Bosch‐Presegue and A. Vaquero , “Sirtuin‐dependent Epigenetic Regulation in the Maintenance of Genome Integrity,” Febs Journal 282 (2015): 1745–1767. 25223884 10.1111/febs.13053

[174] Q. Gao , F. Chen , L. Zhang , et al., “Inhibition of DNA Methyltransferase Aberrations Reinstates Antioxidant Aging Suppressors and Ameliorates Renal Aging,” Aging Cell 21 (2022): e13526. 34874096 10.1111/acel.13526 PMC8761007

[175] E. Pasyukova , A. Symonenko , O. Rybina , and A. Vaiserman , “Epigenetic Enzymes: A Role in Aging and Prospects for Pharmacological Targeting,” Ageing Research Reviews 67 (2021): 101312. 33657446 10.1016/j.arr.2021.101312

[176] W. Li , W. Tian , G. Yuan , et al., “Molecular Basis of Nucleosomal H3K36 Methylation by NSD Methyltransferases,” Nature 590 (2021): 498–503. 33361816 10.1038/s41586-020-03069-8 PMC7889650

[177] Z. Li , X. Zhang , S. Xie , et al., “H3K36me2 methyltransferase NSD2 Orchestrates Epigenetic Reprogramming During Spermatogenesis,” Nucleic Acids Res. 50 (2022): 6786–6800. 35736136 10.1093/nar/gkac533 PMC9262605

[178] S. Xiong , J. Zhou , and W. Chng , “Deciphering the Dynamics of Histone Acetylation and Chromatin Remodeling in Multiple Myeloma: A Tale Beyond the Tails,” Blood (2025): blood/202502840, 10.1182/blood.2025028403. 40608868

[179] A. Pashos , A. Meyer , C. Bussey‐Sutton , et al., “H3K36 methylation Regulates Cell Plasticity and Regeneration in the Intestinal Epithelium,” Nature Cell Biology 27 (2025): 202–217. 39779942 10.1038/s41556-024-01580-y PMC12342706

[180] Y. Huang , X. Yang , Y. Wang , et al., “ARID1A recruits GATA2 to Regulate the Senescence of Trophoblast Cells Under High‐glucose Condition,” Placenta 158 (2024): 156–164. 39490111 10.1016/j.placenta.2024.10.012

[181] Z. Li , S. Zhu , X. Chen , et al., “ARID1A suppresses Malignant Transformation of human Pancreatic Cells via Mediating Senescence‐associated miR‐503/CDKN2A Regulatory Axis,” Biochemical and Biophysical Research Communications 493 (2017): 1018–1025. 28942143 10.1016/j.bbrc.2017.09.099

[182] B. Cabot and R. Cabot , “Chromatin Remodeling in Mammalian Embryos,” Reproduction (Cambridge, England) 155 (2018): R147–R158. 29339454 10.1530/REP-17-0488

[183] P. Mittal and C. Roberts , “The SWI/SNF Complex in Cancer—biology, Biomarkers and Therapy,” Nature reviews Clinical oncology 17 (2020): 435–448. 10.1038/s41571-020-0357-3 PMC8723792 32303701

[184] X. Li , G. Zhu , and B. Zhao , “Chromatin Remodeling in Tissue Stem Cell Fate Determination,” Cell Regen 13 (2024): 18. 39348027 10.1186/s13619-024-00203-z PMC11442411

[185] D. Zhu , X. Wu , J. Zhou , et al., “NuRD Mediates Mitochondrial Stress‐induced Longevity via Chromatin Remodeling in Response to Acetyl‐CoA Level,” Science Advances 6 (2020): eabb2529. 32789178 10.1126/sciadv.abb2529 PMC7400466

[186] J. Basta and M. Rauchman , “The Nucleosome Remodeling and Deacetylase Complex in Development and Disease,” Transl Res 165 (2015): 36–47. 24880148 10.1016/j.trsl.2014.05.003 PMC4793962

[187] N. Golden , M. Foley , K. Kim Guisbert , and E. Guisbert , “Divergent Regulatory Roles of NuRD Chromatin Remodeling Complex Subunits GATAD2 and CHD4 in Caenorhabditis elegans,” Genetics 221 (2022): iyac046. 35323946 10.1093/genetics/iyac046 PMC9071545

[188] P. Canale , J. Campolo , A. Borghini , and M. Andreassi , “Long Telomeric Repeat‐Containing RNA (TERRA): Biological Functions and Challenges in Vascular Aging and Disease,” Biomedicines 11 (2023): 3211. 38137431 10.3390/biomedicines11123211 PMC10740775

[189] G. Libertini , G. Corbi , and F. Nicola , “Importance and Meaning of TERRA Sequences for Aging Mechanisms,” Biochemistry (Mosc) 85 (2020): 1505–1517. 33705290 10.1134/S0006297920120044

[190] E. Mahmoudi and M. Cairns , “CircRNA and Ageing,” Sub‐Cellular Biochemistry 102 (2023): 249–270. 36600136 10.1007/978-3-031-21410-3_10

[191] Q. Hao , L. Wang , M. Zhang , Z. Wang , M. Li , and X. Gao , “Taurine Stimulates Protein Synthesis and Proliferation of C2C12 Myoblast Cells Through the PI3K‐ARID4B‐mTOR Pathway,” British Journal of Nutrition 128 (2022): 1875–1886. 34881695 10.1017/S0007114521004918

[192] D. Xu , H. Sas‐Nowosielska , G. Donahue , et al., “Histone Acetylation in an Alzheimer's Disease Cell Model Promotes Homeostatic Amyloid‐reducing Pathways,” Acta Neuropathol Commun 12 (2024): 3. 38167174 10.1186/s40478-023-01696-6 PMC10759377

[193] L. Shu , Y. Zhang , Q. Sun , H. Pan , J. Guo , and B. Tang , “SNCA REP1 and Parkinson's Disease,” Neuroscience Letters 682 (2018): 79–84. 29859327 10.1016/j.neulet.2018.05.043

[194] A. Cherian , P. K , and A. Vijayaraghavan , “Parkinson's Disease—genetic Cause,” Current Opinion in Neurology 36 (2023): 292–301. 37366140 10.1097/WCO.0000000000001167

[195] H. Luo , G. Zhu , M. Eshelman , et al., “HOTTIP‐dependent R‐loop Formation Regulates CTCF Boundary Activity and TAD Integrity in Leukemia,” Molecular Cell 82 (2022): 833–851. 35180428 10.1016/j.molcel.2022.01.014 PMC8985430

[196] N. Ebrahim , K. Shakirova , and E. Dashinimaev , “PDX1 is the Cornerstone of Pancreatic Beta‐cell Functions and Identity,” Frontiers in Molecular Biosciences 9 (2022): 1091757. 36589234 10.3389/fmolb.2022.1091757 PMC9798421

[197] M. Chen , Z. Zhang , J. Ke , et al., “Chaetocin Attenuates Atherosclerosis Progression and Inhibits Vascular Smooth Muscle Cell Phenotype Switching,” J Cardiovasc Transl Res 15 (2022): 1270–1282. 35428928 10.1007/s12265-022-10258-5

[198] C. Chronis , P. Fiziev , B. Papp , et al., “Cooperative Binding of Transcription Factors Orchestrates Reprogramming,” Cell 168 (2017): 442–459. e420. 28111071 10.1016/j.cell.2016.12.016 PMC5302508

[199] S. Horvath , E. Lacunza , M. Mallat , et al., “Cognitive Rejuvenation in Old Rats by Hippocampal OSKM Gene Therapy,” Geroscience 47 (2025): 809–823. 39037528 10.1007/s11357-024-01269-y PMC11872836

[200] A. Farsetti , B. Illi , and C. Gaetano , “How Epigenetics Impacts on human Diseases,” Eur J Intern Med 114 (2023): 15–22. 37277249 10.1016/j.ejim.2023.05.036

[201] A. Barral , G. Pozo , L. Ducrot , et al., “SETDB1/NSD‐dependent H3K9me3/H3K36me3 Dual Heterochromatin Maintains Gene Expression Profiles by Bookmarking Poised Enhancers,” Molecular Cell 82 (2022): 816–832. e812. 35081363 10.1016/j.molcel.2021.12.037 PMC8860380

[202] A. Korotkov , A. Seluanov , and V. Gorbunova , “Sirtuin 6: Linking Longevity With Genome and Epigenome Stability,” Trends in Cell Biology 31 (2021): 994–1006. 34281779 10.1016/j.tcb.2021.06.009 PMC8903056

[203] T. Hou , Z. Cao , J. Zhang , et al., “SIRT6 coordinates With CHD4 to Promote Chromatin Relaxation and DNA Repair,” Nucleic Acids Res. 48 (2020): 2982–3000. 31970415 10.1093/nar/gkaa006 PMC7102973

[204] R. Cai , R. Lv , X. Shi , G. Yang , and J. Jin , “CRISPR/dCas9 Tools: Epigenetic Mechanism and Application in Gene Transcriptional Regulation,” International Journal of Molecular Sciences 24 (2023): 14865. 37834313 10.3390/ijms241914865 PMC10573330

[205] M. Rahman and T. Tollefsbol , “Targeting Cancer Epigenetics With CRISPR‐dCAS9: Principles and Prospects,” Methods (San Diego, Calif.) 187 (2021): 77–91. 32315755 10.1016/j.ymeth.2020.04.006 PMC7572534

[206] T. Thum , “Non‐coding RNAs in Ageing,” Ageing Research Reviews 17 (2014): 1–2. 25152449 10.1016/j.arr.2014.08.001

[207] M. Moqri , C. Herzog , J. Poganik , et al., “Biomarkers of Aging for the Identification and Evaluation of Longevity Interventions,” Cell 186 (2023): 3758–3775. 37657418 10.1016/j.cell.2023.08.003 PMC11088934

[208] W. Lai , M. Lin , and W. Wong , “Tackling Aging by Using miRNA as a Target and a Tool,” Trends in Molecular Medicine 25 (2019): 673–684. 31126873 10.1016/j.molmed.2019.04.007

[209] J. Wagner , L. Tombor , P. Malacarne , et al., “Aging Impairs the Neurovascular Interface in the Heart,” Science 381 (2023): 897–906. 37616346 10.1126/science.ade4961

[210] V. Swahari , A. Nakamura , E. Hollville , et al., “miR‐29 Is an Important Driver of Aging‐related Phenotypes,” Communications Biology 7 (2024): 1055. 39191864 10.1038/s42003-024-06735-z PMC11349983

[211] Y. Bi , X. Qiao , Z. Cai , et al., “Exosomal miR‐302b Rejuvenates Aging Mice by Reversing the Proliferative Arrest of Senescent Cells,” Cell metabolism 37 (2025): 527–541. e526. 39818209 10.1016/j.cmet.2024.11.013

[212] T. Smith‐Vikos and F. Slack , “MicroRNAs and Their Roles in Aging,” Journal of Cell Science 125 (2012): 7–17. 22294612 10.1242/jcs.099200 PMC3269020

[213] X. Bai , Y. Ma , R. Ding , B. Fu , S. Shi , and X. Chen , “miR‐335 and miR‐34a Promote Renal Senescence by Suppressing Mitochondrial Antioxidative Enzymes,” Journal of the American Society of Nephrology 22 (2011): 1252–1261. 21719785 10.1681/ASN.2010040367 PMC3137573

[214] D. Bhaumik , G. Scott , S. Schokrpur , et al., “MicroRNAs miR‐146a/b Negatively Modulate the Senescence‐associated Inflammatory Mediators IL‐6 and IL‐8,” Aging (Albany NY) 1 (2009): 402–411. 20148189 10.18632/aging.100042 PMC2818025

[215] S. Ghafouri‐Fard , A. Abak , S. Talebi , et al., “Role of miRNA and lncRNAs in Organ Fibrosis and Aging,” Biomedicine & Pharmacotherapy 143 (2021): 112132. 34481379 10.1016/j.biopha.2021.112132

[216] Z. Yang , S. Jiang , J. Shang , et al., “LncRNA: Shedding Light on Mechanisms and Opportunities in Fibrosis and Aging,” Ageing Research Reviews 52 (2019): 17–31. 30954650 10.1016/j.arr.2019.04.001

[217] H. Gao , E. Nepovimova , Z. Heger , et al., “Role of Hypoxia in Cellular Senescence,” Pharmacological Research 194 (2023): 106841. 37385572 10.1016/j.phrs.2023.106841

[218] B. Wang , C. Suen , H. Ma , et al., “The Roles of H19 in Regulating Inflammation and Aging,” Frontiers in immunology 11 (2020): 579687. 33193379 10.3389/fimmu.2020.579687 PMC7653221

[219] L. Nasiri , M. Vaez‐Mahdavi , H. Hassanpour , et al., “Transcription of Biological Aging Markers (ANRIL, P16(INK4a), TBX2, and TERRA) and Their Correlations With Severity of Sulfur Mustard Exposure in Veterans,” Drug and Chemical Toxicology (2024): 1–9, 10.1080/01480545.2024.2395571. 39227349

[220] Y. Kotake , T. Nakagawa , K. Kitagawa , et al., “Long Non‐coding RNA ANRIL Is Required for the PRC2 Recruitment to and Silencing of p15(INK4B) Tumor Suppressor Gene,” Oncogene 30 (2011): 1956–1962. 21151178 10.1038/onc.2010.568 PMC3230933

[221] S. Hu , J. Zheng , Z. Du , and G. Wu , “Knock Down of lncRNA H19 Promotes Axon Sprouting and Functional Recovery After Cerebral Ischemic Stroke,” Brain Research 1732 (2020): 146681. 31991123 10.1016/j.brainres.2020.146681

[222] C. Ma , K. Nong , H. Zhu , et al., “H19 promotes Pancreatic Cancer Metastasis by Derepressing let‐7's Suppression on Its Target HMGA2‐mediated EMT,” Tumour Biology: The Journal of the International Society for Oncodevelopmental Biology and Medicine 35 (2014): 9163–9169. 24920070 10.1007/s13277-014-2185-5

[223] H. Gruner , M. Cortes‐Lopez , D. Cooper , M. Bauer , and P. Miura , “CircRNA Accumulation in the Aging Mouse Brain,” Scientific Reports 6 (2016): 38907. 27958329 10.1038/srep38907 PMC5153657

[224] D. Knupp and P. Miura , “CircRNA Accumulation: A New Hallmark of Aging?” Mechanisms of Ageing and Development 173 (2018): 71–79. 29753875 10.1016/j.mad.2018.05.001 PMC6191176

[225] L. Zhang , Y. Zhang , F. Yu , X. Li , H. Gao , and P. Li , “The circRNA‐miRNA/RBP Regulatory Network in Myocardial Infarction,” Frontiers in pharmacology 13 (2022): 941123. 35924059 10.3389/fphar.2022.941123 PMC9340152

[226] N. Liu , Z. Zhang , Y. Wu , Y. Wang , and Y. Liang , “CRBSP:Prediction of CircRNA‐RBP Binding Sites Based on Multimodal Intermediate Fusion,” IEEE/ACM Trans Comput Biol Bioinform 20 (2023): 2898–2906. 37130249 10.1109/TCBB.2023.3272400

[227] F. Ding , L. Lu , C. Wu , et al., “circHIPK3 prevents Cardiac Senescence by Acting as a Scaffold to Recruit Ubiquitin Ligase to Degrade HuR,” Theranostics 12 (2022): 7550–7566. 36438474 10.7150/thno.77630 PMC9691369

[228] Y. Xu , Z. Zai , Z. Lu , et al., “Circular RNA CircCDKN2B‐AS_006 Promotes the Tumor‐Like Growth and Metastasis of Rheumatoid Arthritis Synovial Fibroblasts by Targeting the miR‐1258/RUNX1 Axis,” International Journal of Molecular Sciences 24 (2023): 5880. 36982956 10.3390/ijms24065880 PMC10051600

[229] C. Zhao , X. Li , G. Sun , et al., “CircFOXO3 protects Against Osteoarthritis by Targeting Its Parental Gene FOXO3 and Activating PI3K/AKT‐mediated Autophagy,” Cell death & disease 13 (2022): 932. 36344492 10.1038/s41419-022-05390-8 PMC9640610

[230] Y. Zhang , W. Zhu , F. Qi , and F. Che , “CircHIPK3 promotes Neuroinflammation Through Regulation of the miR‐124‐3p/STAT3/NLRP3 Signaling Pathway in Parkinson's Disease,” Advances in clinical and experimental medicine: official organ Wroclaw Medical University 32 (2023): 315–329. 36306116 10.17219/acem/154658

[231] E. Lecona , L. Rojas , R. Bonasio , A. Johnston , O. Fernandez‐Capetillo , and D. Reinberg , “Polycomb Protein SCML2 Regulates the Cell Cycle by Binding and Modulating CDK/CYCLIN/p21 Complexes,” Plos Biology 11 (2013): e1001737. 24358021 10.1371/journal.pbio.1001737 PMC3866099

[232] W. Du , W. Yang , E. Liu , Z. Yang , P. Dhaliwal , and B. Yang , “Foxo3 circular RNA Retards Cell Cycle Progression via Forming Ternary Complexes With p21 and CDK2,” Nucleic Acids Res. 44 (2016): 2846–2858. 26861625 10.1093/nar/gkw027 PMC4824104

[233] A. Seyhan , “Trials and Tribulations of MicroRNA Therapeutics,” International Journal of Molecular Sciences 25 (2024): 1469. 38338746 10.3390/ijms25031469 PMC10855871

[234] S. Anver , A. Sumit , X. Sun , et al., “Ageing‐associated Long Non‐coding RNA Extends Lifespan and Reduces Translation in Non‐dividing Cells,” Embo Reports 25 (2024): 4921–4949. 39358553 10.1038/s44319-024-00265-9 PMC11549352

[235] W. Schain , “Psychosocial Issues in Breast Cancer Clinical Trials,” Recent Results in Cancer Research 127 (1993): 235–241. 8502821 10.1007/978-3-642-84745-5_31

[236] M. Fitz‐James and G. Cavalli , “Molecular Mechanisms of Transgenerational Epigenetic Inheritance,” Nature Reviews Genetics 23 (2022): 325–341. 10.1038/s41576-021-00438-5 PMC7619059 34983971

[237] M. Xavier , S. Roman , R. Aitken , and B. Nixon , “Transgenerational Inheritance: How Impacts to the Epigenetic and Genetic Information of Parents Affect Offspring Health,” Human Reproduction Update 25 (2019): 518–540. 31374565 10.1093/humupd/dmz017

[238] K. Moelling , “Epigenetics and Transgenerational Inheritance,” The Journal of Physiology 602 (2024): 2537–2545. 37772441 10.1113/JP284424

[239] E. Lister‐Shimauchi , B. McCarthy , M. Lippincott , and S. Ahmed , “Genetic and Epigenetic Inheritance at Telomeres,” Epigenomes 6 (2022): 9. 35323213 10.3390/epigenomes6010009 PMC8947350

[240] M. Haberman , T. Menashe , N. Cohen , et al., “Paternal High‐fat Diet Affects Weight and DNA Methylation of Their Offspring,” Scientific Reports 14 (2024): 19874. 39191806 10.1038/s41598-024-70438-y PMC11349951

[241] Q. Zhang , X. Xiao , J. Zheng , et al., “Maternal High‐Fat Diet Disturbs the DNA Methylation Profile in the Brown Adipose Tissue of Offspring Mice,” Front Endocrinol (Lausanne) 12 (2021): 705827. 34690924 10.3389/fendo.2021.705827 PMC8531551

[242] L. Oblak , J. van der Zaag , A. Higgins‐Chen , M. Levine , and M. Boks , “A Systematic Review of Biological, Social and Environmental Factors Associated With Epigenetic Clock Acceleration,” Ageing Research Reviews 69 (2021): 101348. 33930583 10.1016/j.arr.2021.101348

[243] P. Anjaria , V. Asediya , J. Nayak , and P. Koringa , “The Epigenetic Landscape: How Environmental Cues Shape Gene Expression,” Epigenomics 15 (2023): 267–270. 37194578 10.2217/epi-2023-0112

[244] G. Lidzbarsky , D. Gutman , H. Shekhidem , L. Sharvit , and G. Atzmon , “Genomic Instabilities, Cellular Senescence, and Aging: In Vitro, in Vivo and Aging‐Like Human Syndromes,” Front Med (Lausanne) 5 (2018): 104. 29719834 10.3389/fmed.2018.00104 PMC5913290

[245] A. Brooks‐Wilson , “Genetics of Healthy Aging and Longevity,” Human Genetics 132 (2013): 1323–1338. 23925498 10.1007/s00439-013-1342-z PMC3898394

[246] M. Petr , T. Tulika , L. Carmona‐Marin , and M. Scheibye‐Knudsen , “Protecting the Aging Genome,” Trends in Cell Biology 30 (2020): 117–132. 31917080 10.1016/j.tcb.2019.12.001

[247] A. Aguilera and B. Gomez‐Gonzalez , “Genome Instability: A Mechanistic View of Its Causes and Consequences,” Nature Reviews Genetics 9 (2008): 204–217. 10.1038/nrg2268 18227811

[248] A. Aguilera and T. Garcia‐Muse , “Causes of Genome Instability,” Annual Review of Genetics 47 (2013): 1–32. 10.1146/annurev-genet-111212-133232 23909437

[249] V. Gorbunova , A. Seluanov , Z. Mao , and C. Hine , “Changes in DNA Repair During Aging,” Nucleic Acids Res. 35 (2007): 7466–7474. 17913742 10.1093/nar/gkm756 PMC2190694

[250] A. Tubbs and A. Nussenzweig , “Endogenous DNA Damage as a Source of Genomic Instability in Cancer,” Cell 168 (2017): 644–656. 28187286 10.1016/j.cell.2017.01.002 PMC6591730

[251] J. Ruszkiewicz , A. Burkle , and A. Mangerich , “Fueling Genome Maintenance: On the Versatile Roles of NAD(+) in Preserving DNA Integrity,” Journal of Biological Chemistry 298 (2022): 102037. 35595095 10.1016/j.jbc.2022.102037 PMC9194868

[252] R. Prakash , Y. Zhang , W. Feng , and M. Jasin , “Homologous Recombination and human Health: The Roles of BRCA1, BRCA2, and Associated Proteins,” Cold Spring Harbor perspectives in biology 7 (2015): a016600. 25833843 10.1101/cshperspect.a016600 PMC4382744

[253] K. Karakostis , L. Malbert‐Colas , A. Thermou , B. Vojtesek , and R. Fahraeus , “The DNA Damage Sensor ATM Kinase Interacts With the p53 mRNA and Guides the DNA Damage Response Pathway,” Molecular cancer 23 (2024): 21. 38263180 10.1186/s12943-024-01933-z PMC10804554

[254] Z. Mirman , K. Sharma , T. Carroll , and T. de Lange , “Expression of BRCA1, BRCA2, RAD51, and Other DSB Repair Factors Is Regulated by CRL4(WDR70),” Dna Repair 113 (2022): 103320. 35316728 10.1016/j.dnarep.2022.103320 PMC9474743

[255] E. Fang , H. Kassahun , D. Croteau , et al., “NAD(+) Replenishment Improves Lifespan and Healthspan in Ataxia Telangiectasia Models via Mitophagy and DNA Repair,” Cell metabolism 24 (2016): 566–581. 27732836 10.1016/j.cmet.2016.09.004 PMC5777858

[256] A. Wilk , F. Hayat , R. Cunningham , et al., “Extracellular NAD(+) Enhances PARP‐dependent DNA Repair Capacity Independently of CD73 Activity,” Scientific Reports 10 (2020): 651. 31959836 10.1038/s41598-020-57506-9 PMC6971268

[257] J. Alhmoud , J. Woolley , A. Al Moustafa , and M. Malki , “DNA Damage/Repair Management in Cancers,” Cancers (Basel) 12 (2020): 1050. 32340362 10.3390/cancers12041050 PMC7226105

[258] J. He , X. Fu , M. Zhang , et al., “Transposable Elements Are Regulated by Context‐specific Patterns of Chromatin Marks in Mouse Embryonic Stem Cells,” Nature Communications 10 (2019): 34. 10.1038/s41467-018-08006-y PMC6318327 30604769

[259] A. Fernandez , C. O'Leary , K. O'Byrne , J. Burgess , D. Richard , and A. Suraweera , “Epigenetic Mechanisms in DNA Double Strand Break Repair: A Clinical Review,” Frontiers in Molecular Biosciences 8 (2021): 685440. 34307454 10.3389/fmolb.2021.685440 PMC8292790

[260] R. Kumari and P. Jat , “Mechanisms of Cellular Senescence: Cell Cycle Arrest and Senescence Associated Secretory Phenotype,” Frontiers in Cell and Developmental Biology 9 (2021): 645593. 33855023 10.3389/fcell.2021.645593 PMC8039141

[261] Y. Qin , H. Liu , and H. Wu , “Cellular Senescence in Health, Disease, and Lens Aging,” Pharmaceuticals (Basel) 18 (2025): 244. 40006057 10.3390/ph18020244 PMC11859104

[262] R. Tenchov , J. Sasso , X. Wang , and Q. Zhou , “Aging Hallmarks and Progression and Age‐Related Diseases: A Landscape View of Research Advancement,” Acs Chemical Neuroscience 15 (2024): 1–30. 38095562 10.1021/acschemneuro.3c00531 PMC10767750

[263] S. He and N. Sharpless , “Senescence in Health and Disease,” Cell 169 (2017): 1000–1011. 28575665 10.1016/j.cell.2017.05.015 PMC5643029

[264] X. Huang , L. Huang , J. Lu , et al., “The Relationship Between Telomere Length and Aging‐related Diseases,” Clinical and Experimental Medicine 25 (2025): 72. 40044947 10.1007/s10238-025-01608-z PMC11882723

[265] J. de Magalhaes and J. Passos , “Stress, Cell Senescence and Organismal Ageing,” Mechanisms of Ageing and Development 170 (2018): 2–9. 28688962 10.1016/j.mad.2017.07.001

[266] R. Benetti , M. Garcia‐Cao , and M. Blasco , “Telomere Length Regulates the Epigenetic Status of Mammalian Telomeres and Subtelomeres,” Nature Genetics 39 (2007): 243–250. 17237781 10.1038/ng1952

[267] S. Gadalla , H. Katki , F. Shebl , N. Giri , B. Alter , and S. Savage , “The Relationship Between DNA Methylation and Telomere Length in Dyskeratosis Congenita,” Aging Cell 11 (2012): 24–28. 21981348 10.1111/j.1474-9726.2011.00755.x PMC3257380

[268] L. Chen , C. Zhang , W. Ma , J. Huang , Y. Zhao , and H. Liu , “METTL3‐mediated m6A Modification Stabilizes TERRA and Maintains Telomere Stability,” Nucleic Acids Res. 50 (2022): 11619–11634. 36399511 10.1093/nar/gkac1027 PMC9723618

[269] H. Tang , H. Wang , X. Cheng , et al., “HuR Regulates Telomerase Activity Through TERC Methylation,” Nature Communications 9 (2018): 2213. 10.1038/s41467-018-04617-7 PMC5992219 29880812

[270] X. Cheng , X. Gu , T. Xia , et al., “HuB and HuD Repress Telomerase Activity by Dissociating HuR From TERC,” Nucleic Acids Res. 49 (2021): 2848–2858. 33589924 10.1093/nar/gkab062 PMC7969021

[271] J. Rivosecchi , K. Jurikova , and E. Cusanelli , “Telomere‐specific Regulation of TERRA and Its Impact on Telomere Stability,” Seminars in cell & developmental biology 157 (2024): 3–23. 38088000 10.1016/j.semcdb.2023.11.001

[272] F. Milosic , M. Hengstschlager , and S. Osmanagic‐Myers , “Premature Aging in Genetic Diseases: What Conclusions Can be Drawn for Physiological Aging,” Front Aging 4 (2023): 1327833. 38481648 10.3389/fragi.2023.1327833 PMC10933081

[273] S. Maynard , E. Fang , M. Scheibye‐Knudsen , D. Croteau , and V. Bohr , “DNA Damage, DNA Repair, Aging, and Neurodegeneration,” Cold Spring Harbor perspectives in medicine 5 (2015): a025130. 26385091 10.1101/cshperspect.a025130 PMC4588127

[274] M. Li , H. Guo , M. Carey , and C. Huang , “Transcriptional and Epigenetic Dysregulation Impairs Generation of Proliferative Neural Stem and Progenitor Cells During Brain Aging,” Nat Aging 4 (2024): 62–79. 38177329 10.1038/s43587-023-00549-0 PMC10947366

[275] G. Lupo , P. Nisi , P. Esteve , et al., “Molecular Profiling of Aged Neural Progenitors Identifies Dbx2 as a Candidate Regulator of Age‐associated Neurogenic Decline,” Aging Cell 17 (2018): e12745. 29504228 10.1111/acel.12745 PMC5946077

[276] J. Wood , B. Jones , N. Jiang , et al., “Chromatin‐modifying Genetic Interventions Suppress Age‐associated Transposable Element Activation and Extend Life Span in Drosophila,” PNAS 113 (2016): 11277–11282. 27621458 10.1073/pnas.1604621113 PMC5056045

[277] A. Du , J. Chobirko , X. Zhuo , C. Feschotte , and T. Wang , “Regulatory Transposable Elements in the Encyclopedia of DNA Elements,” Nature Communications 15 (2024): 7594. 10.1038/s41467-024-51921-6 PMC11366022 39217141

[278] C. Mrabti , N. Yang , G. Desdin‐Mico , et al., “Loss of H3K9 Trimethylation Leads to Premature Aging,” BioRxiv (2024), 10.1101/2024.07.24.604929.

[279] B. Benayoun , E. Pollina , and A. Brunet , “Epigenetic Regulation of Ageing: Linking Environmental Inputs to Genomic Stability,” Nature Reviews Molecular Cell Biology 16 (2015): 593–610. 26373265 10.1038/nrm4048 PMC4736728

[280] A. Gebrie , “Transposable Elements as Essential Elements in the Control of Gene Expression,” Mob DNA 14 (2023): 9. 37596675 10.1186/s13100-023-00297-3 PMC10439571

[281] C. Ravel‐Godreuil , R. Znaidi , T. Bonnifet , R. Joshi , and J. Fuchs , “Transposable Elements as New Players in Neurodegenerative Diseases,” Febs Letters 595 (2021): 2733–2755. 34626428 10.1002/1873-3468.14205

[282] F. Della Valle , P. Reddy , A. Aguirre Vazquez , and J. Izpisua Belmonte , “Reactivation of Retrotransposable Elements Is Associated With Environmental Stress and Ageing,” Nature Reviews Genetics 26 (2025): 547–558. 10.1038/s41576-025-00829-y 40175591

[283] B. McCauley , L. Sun , R. Yu , et al., “Altered Chromatin States Drive Cryptic Transcription in Aging Mammalian Stem Cells,” Nat Aging 1 (2021): 684–697. 34746802 10.1038/s43587-021-00091-x PMC8570536

[284] C. Li , K. Chen , X. Li , and X. Xiong , “Epitranscriptome‐epigenome Interactions in Development and Disease Mechanisms,” Trends in Genetics 41, no. 8 (2025): 691–705, 10.1016/j.tig.2025.04.009. 40374434

[285] M. Schosserer , N. Minois , T. Angerer , et al., “Corrigendum: Methylation of Ribosomal RNA by NSUN5 Is a Conserved Mechanism Modulating Organismal Lifespan,” Nature Communications 7 (2016): 11530. 10.1038/ncomms11530 PMC4865849 27167997

[286] K. Lei , S. Lin , and Q. Yuan , “N6‐methyladenosine (m6A) Modification of Ribosomal RNAs (rRNAs): Critical Roles in mRNA Translation and Diseases,” Genes Dis 10 (2023): 126–134. 37013049 10.1016/j.gendis.2021.10.005 PMC10066336

[287] X. He , S. Memczak , J. Qu , J. Belmonte , and G. Liu , “Single‐cell Omics in Ageing: A Young and Growing Field,” Nat Metab 2 (2020): 293–302. 32694606 10.1038/s42255-020-0196-7

[288] A. Papantonis , A. Antebi , L. Partridge , and A. Beyer , “Age‐associated Changes in Transcriptional Elongation and Their Effects on Homeostasis,” Trends in Cell Biology 35, no. 8 (2024): 645–650, 10.1016/j.tcb.2024.11.005. 39706758

[289] J. Neves , P. Sousa‐Victor , and H. Jasper , “Rejuvenating Strategies for Stem Cell‐Based Therapies in Aging,” Cell Stem Cell 20 (2017): 161–175. 28157498 10.1016/j.stem.2017.01.008 PMC5681350

[290] D. Saavedra , A. Ane‐Kouri , N. Barzilai , et al., “Aging and Chronic Inflammation: Highlights From a Multidisciplinary Workshop,” Immun Ageing 20 (2023): 25. 37291596 10.1186/s12979-023-00352-w PMC10248980

[291] B. Liu , J. Qu , W. Zhang , J. Izpisua Belmonte , and G. Liu , “A Stem Cell Aging Framework, From Mechanisms to Interventions,” Cell reports 41 (2022): 111451. 36261013 10.1016/j.celrep.2022.111451

[292] A. Ahmed , M. Sheng , S. Wasnik , D. Baylink , and K. Lau , “Effect of Aging on Stem Cells,” World J Exp Med 7 (2017): 1–10. 28261550 10.5493/wjem.v7.i1.1 PMC5316899

[293] C. Chen , L. Zhang , R. Dutta , et al., “SRCAP Mutations Drive Clonal Hematopoiesis Through Epigenetic and DNA Repair Dysregulation,” Cell Stem Cell 30 (2023): 1503–1519. e1508. 37863054 10.1016/j.stem.2023.09.011 PMC10841682

[294] R. Yan , X. Cheng , C. Gu , et al., “Dynamics of DNA Hydroxymethylation and Methylation During Mouse Embryonic and Germline Development,” Nature Genetics 55 (2023): 130–143. 36539615 10.1038/s41588-022-01258-x

[295] H. Blau , B. Cosgrove , and A. Ho , “The central Role of Muscle Stem Cells in Regenerative Failure With Aging,” Nature Medicine 21 (2015): 854–862. 10.1038/nm.3918 PMC4731230 26248268

[296] Z. Chen , M. Rasheed , and Y. Deng , “The Epigenetic Mechanisms Involved in Mitochondrial Dysfunction: Implication for Parkinson's Disease,” Brain Pathology 32 (2022): e13012. 34414627 10.1111/bpa.13012 PMC9048811

[297] R. Wang , H. Sun , G. Wang , and H. Ren , “Imbalance of Lysine Acetylation Contributes to the Pathogenesis of Parkinson's Disease,” International Journal of Molecular Sciences 21 (2020): 7182. 33003340 10.3390/ijms21197182 PMC7582258

[298] X. Gao , J. Ding , J. Xie , and H. Xu , “Epigenetic Regulation of Iron Metabolism and Ferroptosis in Parkinson's disease: Identifying Novel Epigenetic Targets,” Acta Pharmacologica Sinica 46 (2025): 2075–2092. 40069488 10.1038/s41401-025-01499-6 PMC12274621

[299] M. Vermulst , J. Bielas , G. Kujoth , et al., “Mitochondrial Point Mutations Do Not Limit the Natural Lifespan of Mice,” Nature Genetics 39 (2007): 540–543. 17334366 10.1038/ng1988

[300] A. Trifunovic , A. Wredenberg , M. Falkenberg , et al., “Premature Ageing in Mice Expressing Defective Mitochondrial DNA Polymerase,” Nature 429 (2004): 417–423. 15164064 10.1038/nature02517

[301] E. Lesnefsky , Q. Chen , and C. Hoppel , “Mitochondrial Metabolism in Aging Heart,” Circulation Research 118 (2016): 1593–1611. 27174952 10.1161/CIRCRESAHA.116.307505 PMC5009371

[302] M. Falk , “Neurodevelopmental Manifestations of Mitochondrial Disease,” Journal of Developmental and Behavioral Pediatrics 31 (2010): 610–621. 20814259 10.1097/DBP.0b013e3181ef42c1 PMC3923321

[303] G. Zhang , H. Wei , A. Zhao , et al., “Mitochondrial DNA Leakage: Underlying Mechanisms and Therapeutic Implications in Neurological Disorders,” J Neuroinflammation 22 (2025): 34. 39920753 10.1186/s12974-025-03363-0 PMC11806845

[304] M. Yan , Y. Li , Q. Luo , et al., “Mitochondrial Damage and Activation of the Cytosolic DNA Sensor cGAS‐STING Pathway Lead to Cardiac Pyroptosis and Hypertrophy in Diabetic Cardiomyopathy Mice,” Cell Death Discov 8 (2022): 258. 35538059 10.1038/s41420-022-01046-w PMC9091247

[305] C. Wiley , M. Velarde , P. Lecot , et al., “Mitochondrial Dysfunction Induces Senescence With a Distinct Secretory Phenotype,” Cell metabolism 23 (2016): 303–314. 26686024 10.1016/j.cmet.2015.11.011 PMC4749409

[306] H. Zhang , D. Ryu , Y. Wu , et al., “NAD(+) Repletion Improves Mitochondrial and Stem Cell Function and Enhances Life Span in Mice,” Science 352 (2016): 1436–1443. 27127236 10.1126/science.aaf2693

[307] S. Stanciu , M. Jinga , D. Miricescu , et al., “mTOR Dysregulation, Insulin Resistance, and Hypertension,” Biomedicines 12 (2024): 1802. 39200267 10.3390/biomedicines12081802 PMC11351979

[308] D. Wang , D. Ji , C. Yu , D. Wu , and L. Qi , “Research Progress on the Mitochondrial Mechanism of Age‐related Non‐alcoholic Fatty Liver,” World Journal of Gastroenterology 29 (2023): 1982–1993. 37155524 10.3748/wjg.v29.i13.1982 PMC10122792

[309] J. Kim , Y. Wei , and J. Sowers , “Role of Mitochondrial Dysfunction in Insulin Resistance,” Circulation Research 102 (2008): 401–414. 18309108 10.1161/CIRCRESAHA.107.165472 PMC2963150

[310] R. Naik , M. Mir , I. Malik , et al., “The Potential Mechanism and the Role of Antioxidants in Mitigating Oxidative Stress in Alzheimer's Disease,” Front Biosci (Landmark Ed) 30 (2025): 25551. 40018917 10.31083/FBL25551

[311] A. Terman , H. Dalen , J. Eaton , J. Neuzil , and U. Brunk , “Aging of Cardiac Myocytes in Culture: Oxidative Stress, Lipofuscin Accumulation, and Mitochondrial Turnover,” Ann N Y Acad Sci 1019 (2004): 70–77. 15246997 10.1196/annals.1297.015

[312] J. Peoples , A. Saraf , N. Ghazal , T. Pham , and J. Kwong , “Mitochondrial Dysfunction and Oxidative Stress in Heart Disease,” Experimental & Molecular Medicine 51 (2019): 1–13. 10.1038/s12276-019-0355-7 PMC6923355 31857574

[313] K. Cao , K. Wang , M. Yang , X. Liu , W. Lv , and J. Liu , “Punicalagin Improves Hepatic Lipid Metabolism via Modulation of Oxidative Stress and Mitochondrial Biogenesis in Hyperlipidemic Mice,” Food Funct 11 (2020): 9624–9633. 32975274 10.1039/d0fo01545h

[314] R. Kanwal and S. Gupta , “Epigenetic Modifications in Cancer,” Clinical Genetics 81 (2012): 303–311. 22082348 10.1111/j.1399-0004.2011.01809.x PMC3590802

[315] A. Eden , F. Gaudet , A. Waghmare , and R. Jaenisch , “Chromosomal Instability and Tumors Promoted by DNA Hypomethylation,” Science 300 (2003): 455. 12702868 10.1126/science.1083557

[316] C. Nguyen , G. Liang , T. Nguyen , et al., “Susceptibility of Nonpromoter CpG Islands to De Novo Methylation in Normal and Neoplastic Cells,” JNCI: Journal of the National Cancer Institute 93 (2001): 1465–1472. 11584062 10.1093/jnci/93.19.1465

[317] L. Borkiewicz , “Histone 3 Lysine 27 Trimethylation Signature in Breast Cancer,” International Journal of Molecular Sciences 22 (2021): 12853. 34884658 10.3390/ijms222312853 PMC8657745

[318] G. Taylor , R. Eskeland , B. Hekimoglu‐Balkan , M. Pradeepa , and W. Bickmore , “H4K16 acetylation Marks Active Genes and Enhancers of Embryonic Stem Cells, but Does Not Alter Chromatin Compaction,” Genome Research 23 (2013): 2053–2065. 23990607 10.1101/gr.155028.113 PMC3847775

[319] P. Das and J. Taube , “Regulating Methylation at H3K27: A Trick or Treat for Cancer Cell Plasticity,” Cancers (Basel) 12 (2020): 2792. 33003334 10.3390/cancers12102792 PMC7600873

[320] Y. Peng , X. Zhang , X. Feng , X. Fan , and Z. Jin , “The Crosstalk Between microRNAs and the Wnt/Beta‐catenin Signaling Pathway in Cancer,” Oncotarget 8 (2017): 14089–14106. 27793042 10.18632/oncotarget.12923 PMC5355165

[321] H. Qian , M. Maghsoudloo , P. Kaboli , et al., “Decoding the Promise and Challenges of miRNA‐Based Cancer Therapies: An Essential Update on miR‐21, miR‐34, and miR‐155,” International journal of medical sciences 21 (2024): 2781–2798. 39512697 10.7150/ijms.102123 PMC11539376

[322] W. Zhang , K. Zhang , J. Shi , et al., “The Impact of the Senescent Microenvironment on Tumorigenesis: Insights for Cancer Therapy,” Aging Cell 23 (2024): e14182. 38650467 10.1111/acel.14182 PMC11113271

[323] A. Suraweera , K. O'Byrne , and D. Richard , “Epigenetic Drugs in Cancer Therapy,” Cancer and Metastasis Reviews 44 (2025): 37. 40011240 10.1007/s10555-025-10253-7 PMC11865116

[324] H. Hampel , R. Vassar , B. De Strooper , et al., “The Beta‐Secretase BACE1 in Alzheimer's Disease,” Biological Psychiatry 89 (2021): 745–756. 32223911 10.1016/j.biopsych.2020.02.001 PMC7533042

[325] L. Lossi , C. Castagna , and A. Merighi , “An Overview of the Epigenetic Modifications in the Brain Under Normal and Pathological Conditions,” International Journal of Molecular Sciences 25 (2024): 3881. 38612690 10.3390/ijms25073881 PMC11011998

[326] M. Caillet‐Boudin , L. Buee , N. Sergeant , and B. Lefebvre , “Regulation of human MAPT Gene Expression,” Mol Neurodegener 10 (2015): 28. 26170022 10.1186/s13024-015-0025-8 PMC4499907

[327] H. Mori , Y. Yoshino , M. Ueno , et al., “Blood MAPT Expression and Methylation Status in Alzheimer's Disease,” PCN Rep 1 (2022): e65. 38868661 10.1002/pcn5.65 PMC11114303

[328] L. de Mena , G. Pares , A. Garrido , et al., “alpha‐Synuclein Gene Hypomethylation in LRRK2 Parkinson's Disease Patients,” Movement Disorders 40 (2025): 550–555. 39711195 10.1002/mds.30094 PMC11926512

[329] L. Wang , L. Liu , C. Han , et al., “Histone Deacetylase 4 Inhibition Reduces Rotenone‐Induced Alpha‐Synuclein Accumulation via Autophagy in SH‐SY5Y Cells,” Brain Sci 13 (2023): 670. 37190635 10.3390/brainsci13040670 PMC10136981

[330] R. Wierda , S. Geutskens , J. Jukema , P. Quax , and P. van den Elsen , “Epigenetics in Atherosclerosis and Inflammation,” Journal of Cellular and Molecular Medicine 14 (2010): 1225–1240. 20132414 10.1111/j.1582-4934.2010.01022.x PMC3828841

[331] X. Chen , Y. He , W. Fu , et al., “Histone Deacetylases (HDACs) and Atherosclerosis: A Mechanistic and Pharmacological Review,” Frontiers in Cell and Developmental Biology 8 (2020): 581015. 33282862 10.3389/fcell.2020.581015 PMC7688915

[332] J. Nikolajevic , N. Ariaee , A. Liew , S. Abbasnia , B. Fazeli , and M. Sabovic , “The Role of MicroRNAs in Endothelial Cell Senescence,” Cells 11 (2022): 1185. 35406749 10.3390/cells11071185 PMC8997793

[333] M. Yamakuchi and T. Hashiguchi , “Endothelial Cell Aging: How miRNAs Contribute?” Journal of Clinical Medicine 7 (2018): 170. 29996516 10.3390/jcm7070170 PMC6068727

[334] K. Crider , T. Yang , R. Berry , and L. Bailey , “Folate and DNA Methylation: A Review of Molecular Mechanisms and the Evidence for Folate's Role,” Advances in nutrition 3 (2012): 21–38. 22332098 10.3945/an.111.000992 PMC3262611

[335] S. Ahmed , S. Ansari , E. Mensah‐Brown , and B. Emerald , “The Role of DNA Methylation in the Pathogenesis of Type 2 Diabetes Mellitus,” Clin Epigenetics 12 (2020): 104. 32653024 10.1186/s13148-020-00896-4 PMC7353744

[336] S. Klimczak and A. Sliwinska , “Epigenetic Regulation of Inflammation in Insulin Resistance,” Seminars in cell & developmental biology 154 (2024): 185–192. 36109307 10.1016/j.semcdb.2022.09.004

[337] H. Ibrahim , “Epigenetic Regulation of Obesity‐Associated Type 2 Diabetes,” Medicina (Kaunas, Lithuania) 58 (2022): 1028–1044. 36295527 10.3390/medicina58101366 PMC9607337

[338] C. Jazieh , T. Arabi , Z. Asim , et al., “Unraveling the Epigenetic Fabric of Type 2 Diabetes Mellitus: Pathogenic Mechanisms and Therapeutic Implications,” Front Endocrinol (Lausanne) 15 (2024): 1295967. 38323108 10.3389/fendo.2024.1295967 PMC10845351

[339] M. Zaiou , “Peroxisome Proliferator‐Activated Receptor‐gamma as a Target and Regulator of Epigenetic Mechanisms in Nonalcoholic Fatty Liver Disease,” Cells 12 (2023): 1205. 37190114 10.3390/cells12081205 PMC10136748

[340] S. Wang , L. Zha , X. Cui , et al., “Epigenetic Regulation of Hepatic Lipid Metabolism by DNA Methylation,” Adv Sci (Weinh) 10 (2023): e2206068. 37282749 10.1002/advs.202206068 PMC10369300

[341] Y. Shi and W. Qi , “Histone Modifications in NAFLD: Mechanisms and Potential Therapy,” International Journal of Molecular Sciences 24 (2023): 14653. 37834101 10.3390/ijms241914653 PMC10572202

[342] S. Pillai , H. Lakhani , M. Zehra , et al., “Predicting Nonalcoholic Fatty Liver Disease Through a Panel of Plasma Biomarkers and MicroRNAs in Female West Virginia Population,” International Journal of Molecular Sciences 21 (2020): 6698. 32933141 10.3390/ijms21186698 PMC7554851

[343] J. Shen , X. Lin , F. Dai , et al., “Ubiquitin‐specific Peptidases: Players in Bone Metabolism,” Cell Proliferation 56 (2023): e13444. 36883930 10.1111/cpr.13444 PMC10392067

[344] L. Hu , W. Chen , A. Qian , and Y. Li , “Wnt/Beta‐catenin Signaling Components and Mechanisms in Bone Formation, Homeostasis, and Disease,” Bone Res 12 (2024): 39. 38987555 10.1038/s41413-024-00342-8 PMC11237130

[345] Y. Zhang , X. Feng , B. Zheng , and Y. Liu , “Regulation and Mechanistic Insights Into Tensile Strain in Mesenchymal Stem Cell Osteogenic Differentiation,” Bone 187 (2024): 117197. 38986825 10.1016/j.bone.2024.117197

[346] L. Zhang , Q. Guan , Z. Wang , J. Feng , J. Zou , and B. Gao , “Consequences of Aging on Bone,” Aging Dis 15 (2023): 2417–2452. 38029404 10.14336/AD.2023.1115 PMC11567267

[347] J. Taylor , J. Wood , E. Mizerak , et al., “Sirt6 regulates Lifespan in Drosophila Melanogaster,” PNAS 119 (2022): e2111176119. 35091469 10.1073/pnas.2111176119 PMC8812521

[348] W. Yu , H. Wang , J. Zhang , and C. Yin , “The Effects of Epigenetic Modifications on Bone Remodeling in Age‐related Osteoporosis,” Connective Tissue Research 64 (2023): 105–116. 36271658 10.1080/03008207.2022.2120392

[349] K. Park‐Min , “Epigenetic Regulation of Bone Cells,” Connective Tissue Research 58 (2017): 76–89. 27082893 10.1080/03008207.2016.1177037 PMC5498111

[350] K. Takahashi and S. Yamanaka , “Induction of Pluripotent Stem Cells From Mouse Embryonic and Adult Fibroblast Cultures by Defined Factors,” Cell 126 (2006): 663–676. 16904174 10.1016/j.cell.2006.07.024

[351] P. Mehrotra , J. Jablonski , J. Toftegaard , et al., “Skeletal Muscle Reprogramming Enhances Reinnervation After Peripheral Nerve Injury,” Nature Communications 15 (2024): 9218. 10.1038/s41467-024-53276-4 PMC11511891 39455585

[352] V. Tabar , H. Sarva , A. Lozano , et al., “Phase I Trial of hES Cell‐derived Dopaminergic Neurons for Parkinson's disease,” Nature 641, no. 8064 (2025): 978–983. 40240592 10.1038/s41586-025-08845-y PMC12095069

[353] N. Sawamoto , D. Doi , E. Nakanishi , et al., “Phase I/II Trial of iPS‐cell‐derived Dopaminergic Cells for Parkinson's disease,” Nature 641, no. 8064 (2025): 971–977, 10.1038/s41586-025-08700-0. 40240591 PMC12095070

[354] L. Pozzo , Z. Xu , S. Lin , et al., “Role of Epigenetics in the Regulation of Skin Aging and Geroprotective Intervention: A New Sight,” Biomedicine & Pharmacotherapy 174 (2024): 116592. 38615608 10.1016/j.biopha.2024.116592

[355] M. Jeong , Y. Lin , S. Wennersten , et al., “Histone Deacetylase Activity Governs Diastolic Dysfunction Through a Nongenomic Mechanism,” Science Translational Medicine 10 (2018): eaao0144. 29437146 10.1126/scitranslmed.aao0144 PMC5908215

[356] M. Walsh , A. Bhattacharya , K. Sataranatarajan , et al., “The Histone Deacetylase Inhibitor Butyrate Improves Metabolism and Reduces Muscle Atrophy During Aging,” Aging Cell 14 (2015): 957–970. 26290460 10.1111/acel.12387 PMC4693467

[357] S. Huang , H. Shi , Z. Shi , J. Wu , and L. He , “Vorinostat, a Potential Hormetin, Extends Lifespan and Enhances Stress Resistance via the SKN‐1 Pathway in Caenorhabditis elegans,” Biogerontology 26 (2025): 97. 40278906 10.1007/s10522-025-10236-9

[358] C. Falckenhayn , A. Bienkowska , J. Sohle , et al., “Identification of Dihydromyricetin as a Natural DNA Methylation Inhibitor With Rejuvenating Activity in human Skin,” Front Aging 4 (2023): 1258184. 38500495 10.3389/fragi.2023.1258184 PMC10944877

[359] S. Yamaguchi , J. Irie , M. Mitsuishi , et al., “Safety and Efficacy of Long‐term Nicotinamide Mononucleotide Supplementation on Metabolism, Sleep, and Nicotinamide Adenine Dinucleotide Biosynthesis in Healthy, Middle‐aged Japanese Men,” Endocrine Journal 71 (2024): 153–169. 38191197 10.1507/endocrj.EJ23-0431

[360] K. Pencina , R. Valderrabano , B. Wipper , et al., “Nicotinamide Adenine Dinucleotide Augmentation in Overweight or Obese Middle‐Aged and Older Adults: A Physiologic Study,” Journal of Clinical Endocrinology and Metabolism 108 (2023): 1968–1980. 36740954 10.1210/clinem/dgad027 PMC11491622

[361] Y. Yang , X. Lu , N. Liu , et al., “Metformin Decelerates Aging Clock in Male Monkeys,” Cell 187 (2024): 6358–6378. e6329. 39270656 10.1016/j.cell.2024.08.021

[362] J. Mannick and D. Lamming , “Targeting the Biology of Aging With mTOR Inhibitors,” Nat Aging 3 (2023): 642–660. 37142830 10.1038/s43587-023-00416-y PMC10330278

[363] S. Horvath , A. Lu , H. Cohen , and K. Raj , “Rapamycin Retards Epigenetic Ageing of Keratinocytes Independently of Its Effects on Replicative Senescence, Proliferation and Differentiation,” Aging (Albany NY) 11 (2019): 3238–3249. 31136303 10.18632/aging.101976 PMC6555449

[364] J. Zhai , W. Kongsberg , Y. Pan , C. Hao , X. Wang , and J. Sun , “Caloric Restriction Induced Epigenetic Effects on Aging,” Frontiers in Cell and Developmental Biology 10 (2022): 1079920. 36712965 10.3389/fcell.2022.1079920 PMC9880295

[365] D. Hernandez‐Saavedra , L. Moody , G. Xu , H. Chen , and Y. Pan , “Epigenetic Regulation of Metabolism and Inflammation by Calorie Restriction,” Advances in nutrition 10 (2019): 520–536. 30915465 10.1093/advances/nmy129 PMC6520046

[366] M. Wei , S. Brandhorst , M. Shelehchi , et al., “Fasting‐mimicking Diet and Markers/Risk Factors for Aging, Diabetes, Cancer, and Cardiovascular Disease,” Science Translational Medicine 9 (2017): eaai8700. 28202779 10.1126/scitranslmed.aai8700 PMC6816332

[367] W. Hastings , Q. Ye , S. Wolf , et al., “Effect of Long‐term Caloric Restriction on Telomere Length in Healthy Adults: CALERIE 2 Trial Analysis,” Aging Cell 23 (2024): e14149. 38504468 10.1111/acel.14149 PMC11296136

[368] Y. Kim , T. Huan , R. Joehanes , et al., “Higher Diet Quality Relates to Decelerated Epigenetic Aging,” American Journal of Clinical Nutrition 115 (2022): 163–170. 34134146 10.1093/ajcn/nqab201 PMC8755029

[369] A. Yaskolka Meir , M. Keller , A. Hoffmann , et al., “The Effect of Polyphenols on DNA Methylation‐assessed Biological Age Attenuation: The DIRECT PLUS Randomized Controlled Trial,” BMC Medicine [Electronic Resource] 21 (2023): 364. 37743489 10.1186/s12916-023-03067-3 PMC10519069

[370] V. Dwaraka , L. Aronica , N. Carreras‐Gallo , et al., “Unveiling the Epigenetic Impact of Vegan vs. omnivorous Diets on Aging: Insights From the Twins Nutrition Study (TwiNS),” BMC Medicine [Electronic Resource] 22 (2024): 301. 39069614 10.1186/s12916-024-03513-w PMC11285457

[371] H. Bischoff‐Ferrari , S. Gangler , M. Wieczorek , et al., “Individual and Additive Effects of Vitamin D, Omega‐3 and Exercise on DNA Methylation Clocks of Biological Aging in Older Adults From the DO‐HEALTH Trial,” Nat Aging 5 (2025): 376–385. 39900648 10.1038/s43587-024-00793-y PMC11922767

[372] J. Chen , J. Xiang , M. Zhou , et al., “Dietary Timing Enhances Exercise by Modulating Fat‐muscle Crosstalk via Adipocyte AMPKalpha2 Signaling,” Cell metabolism (2025), 10.1016/j.cmet.2025.02.007. 40088888

[373] E. Sutton , R. Beyl , K. Early , W. Cefalu , E. Ravussin , and C. Peterson , “Early Time‐Restricted Feeding Improves Insulin Sensitivity, Blood Pressure, and Oxidative Stress Even Without Weight Loss in Men With Prediabetes,” Cell metabolism 27 (2018): 1212–1221. e1213. 29754952 10.1016/j.cmet.2018.04.010 PMC5990470

[374] J. Cavalcanti de Albuquerque , J. Hunter , R. Domingues , et al., “Brain Sensing of Metabolic state Regulates Circulating Monocytes,” Science Immunology 10 (2025): eadr3226. 40184437 10.1126/sciimmunol.adr3226

[375] F. Fox , D. Liu , M. Breteler , and N. Aziz , “Physical Activity Is Associated With Slower Epigenetic Ageing‐Findings From the Rhineland Study,” Aging Cell 22 (2023): e13828. 37036021 10.1111/acel.13828 PMC10265180

[376] A. Keiser , T. Dong , E. Kramar , et al., “Specific Exercise Patterns Generate an Epigenetic Molecular Memory Window That Drives Long‐term Memory Formation and Identifies ACVR1C as a Bidirectional Regulator of Memory in Mice,” Nature Communications 15 (2024): 3836. 10.1038/s41467-024-47996-w PMC11076285 38714691

[377] H. Lee , B. Kim , and T. Park , “The Association Between Sleep Quality and Accelerated Epigenetic Aging With Metabolic Syndrome in Korean Adults,” Clin Epigenetics 16 (2024): 92. 39014432 10.1186/s13148-024-01706-x PMC11253334

[378] K. Fitzgerald , R. Hodges , D. Hanes , et al., “Potential Reversal of Epigenetic Age Using a Diet and Lifestyle Intervention: A Pilot Randomized Clinical Trial,” Aging (Albany NY) 13 (2021): 9419–9432. 33844651 10.18632/aging.202913 PMC8064200

[379] H. Xu , O. Li , D. Kim , Z. Bao , and F. Yang , “Gut Microbiota and Epigenetic Age Acceleration: A bi‐directional Mendelian Randomization Study,” Aging Clinical and Experimental Research 36 (2024): 227. 39612063 10.1007/s40520-024-02877-6 PMC11607049

[380] K. Kim , Y. Chung , J. Huh , et al., “Gut Microbiota of the Young Ameliorates Physical Fitness of the Aged in Mice,” Microbiome 10 (2022): 238. 36567320 10.1186/s40168-022-01386-w PMC9791737

[381] T. Ghosh , F. Shanahan , and P. O'Toole , “The Gut Microbiome as a Modulator of Healthy Ageing,” Nature reviews Gastroenterology & hepatology 19 (2022): 565–584. 35468952 10.1038/s41575-022-00605-x PMC9035980

[382] T. Liu , D. Du , R. Zhao , Q. Xie , and Z. Dong , “Gut Microbes Influence the Development of central Nervous System Disorders Through Epigenetic Inheritance,” Microbiological Research 274 (2023): 127440. 37343494 10.1016/j.micres.2023.127440

[383] Y. Jing , X. Jiang , Q. Ji , et al., “Genome‐wide CRISPR Activation Screening in Senescent Cells Reveals SOX5 as a Driver and Therapeutic Target of Rejuvenation,” Cell Stem Cell 30 (2023): 1452–1471. e1410. 37832549 10.1016/j.stem.2023.09.007

[384] B. Zhang , D. Lee , A. Trapp , et al., “Multi‐omic Rejuvenation and Life Span Extension on Exposure to Youthful Circulation,” Nat Aging 3 (2023): 948–964. 37500973 10.1038/s43587-023-00451-9 PMC11095548

[385] J. Poganik , B. Zhang , G. Baht , et al., “Biological Age Is Increased by Stress and Restored Upon Recovery,” Cell metabolism 35 (2023): 807–820. e805. 37086720 10.1016/j.cmet.2023.03.015 PMC11055493

[386] J. Sanz‐Ros , N. Romero‐Garcia , C. Mas‐Bargues , et al., “Small Extracellular Vesicles From Young Adipose‐derived Stem Cells Prevent Frailty, Improve Health Span, and Decrease Epigenetic Age in Old Mice,” Science Advances 8 (2022): eabq2226. 36260670 10.1126/sciadv.abq2226 PMC9581480

[387] F. Galkin , P. Mamoshina , K. Kochetov , D. Sidorenko , and A. Zhavoronkov , “DeepMAge: A Methylation Aging Clock Developed With Deep Learning,” Aging Dis 12 (2021): 1252–1262. 34341706 10.14336/AD.2020.1202 PMC8279523

[388] A. Teschendorff and S. Horvath , “Epigenetic Ageing Clocks: Statistical Methods and Emerging Computational Challenges,” Nature Reviews Genetics 26 (2025): 350–368. 10.1038/s41576-024-00807-w 39806006

[389] F. Prattichizzo , C. Frige , V. Pellegrini , et al., “Organ‐specific Biological Clocks: Ageotyping for Personalized Anti‐aging Medicine,” Ageing Research Reviews 96 (2024): 102253. 38447609 10.1016/j.arr.2024.102253

[390] M. Buckley , E. Sun , B. George , et al., “Cell‐type‐specific Aging Clocks to Quantify Aging and Rejuvenation in Neurogenic Regions of the Brain,” Nat Aging 3 (2023): 121–137. 37118510 10.1038/s43587-022-00335-4 PMC10154228

[391] K. Browder , P. Reddy , M. Yamamoto , et al., “In Vivo Partial Reprogramming Alters Age‐associated Molecular Changes During Physiological Aging in Mice,” Nat Aging 2 (2022): 243–253. 37118377 10.1038/s43587-022-00183-2

[392] A. Yucel and V. Gladyshev , “The Long and Winding Road of Reprogramming‐induced Rejuvenation,” Nature Communications 15 (2024): 1941. 10.1038/s41467-024-46020-5 PMC10908844 38431638

[393] D. Gill , A. Parry , F. Santos , et al., “Multi‐omic Rejuvenation of human Cells by Maturation Phase Transient Reprogramming,” Elife 11 (2022): e71624. 35390271 10.7554/eLife.71624 PMC9023058

[394] G. Lopez‐Lluch , N. Hunt , B. Jones , et al., “Calorie Restriction Induces Mitochondrial Biogenesis and Bioenergetic Efficiency,” PNAS 103 (2006): 1768–1773. 16446459 10.1073/pnas.0510452103 PMC1413655

[395] J. Yoshino , J. Baur , and S. Imai , “NAD(+) Intermediates: The Biology and Therapeutic Potential of NMN and NR,” Cell metabolism 27 (2018): 513–528. 29249689 10.1016/j.cmet.2017.11.002 PMC5842119

[396] S. Smallwood , H. Lee , C. Angermueller , et al., “Single‐cell Genome‐wide Bisulfite Sequencing for Assessing Epigenetic Heterogeneity,” Nature Methods 11 (2014): 817–820. 25042786 10.1038/nmeth.3035 PMC4117646

[397] O. Schwartzman and A. Tanay , “Single‐cell Epigenomics: Techniques and Emerging Applications,” Nature Reviews Genetics 16 (2015): 716–726. 10.1038/nrg3980 26460349

[398] D. Rossi , D. Bryder , J. Zahn , et al., “Cell Intrinsic Alterations Underlie Hematopoietic Stem Cell Aging,” PNAS 102 (2005): 9194–9199. 15967997 10.1073/pnas.0503280102 PMC1153718

[399] X. Li , X. Zeng , Y. Xu , et al., “Mechanisms and Rejuvenation Strategies for Aged Hematopoietic Stem Cells,” Journal of hematology & oncology 13 (2020): 31. 32252797 10.1186/s13045-020-00864-8 PMC7137344

[400] N. Habib , I. Avraham‐Davidi , A. Basu , et al., “Massively Parallel Single‐nucleus RNA‐seq With DroNc‐seq,” Nature Methods 14 (2017): 955–958. 28846088 10.1038/nmeth.4407 PMC5623139

[401] Z. Shi , Y. Geng , J. Liu , et al., “Single‐cell Transcriptomics Reveals Gene Signatures and Alterations Associated With Aging in Distinct Neural Stem/Progenitor Cell Subpopulations,” Protein Cell 9 (2018): 351–364. 28748452 10.1007/s13238-017-0450-2 PMC5876182

[402] D. Puri and W. Wagner , “Epigenetic Rejuvenation by Partial Reprogramming,” BioEssays 45 (2023): e2200208. 36871150 10.1002/bies.202200208

[403] B. Grigorash , D. van Essen , G. Liang , et al., “p16(High) senescence Restricts Cellular Plasticity During Somatic Cell Reprogramming,” Nature Cell Biology 25 (2023): 1265–1278. 37652981 10.1038/s41556-023-01214-9

[404] B. Wang , J. Han , J. Elisseeff , and M. Demaria , “The Senescence‐associated Secretory Phenotype and Its Physiological and Pathological Implications,” Nature Reviews Molecular Cell Biology 25 (2024): 958–978. 38654098 10.1038/s41580-024-00727-x

[405] M. Baar , R. Brandt , D. Putavet , et al., “Targeted Apoptosis of Senescent Cells Restores Tissue Homeostasis in Response to Chemotoxicity and Aging,” Cell 169 (2017): 132–147. e116. 28340339 10.1016/j.cell.2017.02.031 PMC5556182

[406] Y. Lai , I. Ramirez‐Pardo , J. Isern , et al., “Multimodal Cell Atlas of the Ageing human Skeletal Muscle,” Nature 629 (2024): 154–164. 38649488 10.1038/s41586-024-07348-6 PMC11062927

[407] V. Moiseeva , A. Cisneros , V. Sica , et al., “Senescence Atlas Reveals an Aged‐Like Inflamed Niche That Blunts Muscle Regeneration,” Nature 613 (2023): 169–178. 36544018 10.1038/s41586-022-05535-x PMC9812788

[408] S. Bocklandt , W. Lin , M. Sehl , et al., “Epigenetic Predictor of Age,” PLoS ONE 6 (2011): e14821. 21731603 10.1371/journal.pone.0014821 PMC3120753

[409] M. Levine , “Modeling the Rate of Senescence: Can Estimated Biological Age Predict Mortality More Accurately Than Chronological Age?” Journals of Gerontology. Series A, Biological Sciences and Medical Sciences 68 (2013): 667–674. 23213031 10.1093/gerona/gls233 PMC3660119

[410] B. Joyce , T. Gao , Y. Zheng , et al., “Epigenetic Age Acceleration Reflects Long‐Term Cardiovascular Health,” Circulation Research 129 (2021): 770–781. 34428927 10.1161/CIRCRESAHA.121.318965 PMC8484046

[411] D. Belsky , A. Caspi , R. Houts , et al., “Quantification of Biological Aging in Young Adults,” PNAS 112 (2015): E4104–E4110. 26150497 10.1073/pnas.1506264112 PMC4522793

[412] E. Sillanpaa , A. Heikkinen , A. Kankaanpaa , et al., “Blood and Skeletal Muscle Ageing Determined by Epigenetic Clocks and Their Associations With Physical Activity and Functioning,” Clin Epigenetics 13 (2021): 110. 34001218 10.1186/s13148-021-01094-6 PMC8127311

[413] T. Tanaka , N. Basisty , G. Fantoni , et al., “Plasma Proteomic Biomarker Signature of Age Predicts Health and Life Span,” Elife 9 (2020): e61073. 33210602 10.7554/eLife.61073 PMC7723412

[414] R. Jansen , L. Han , J. Verhoeven , et al., “An Integrative Study of Five Biological Clocks in Somatic and Mental Health,” Elife 10 (2021): e59479. 33558008 10.7554/eLife.59479 PMC7872513

[415] W. Dai , X. Qiao , Y. Fang , et al., “Epigenetics‐targeted Drugs: Current Paradigms and Future Challenges,” Signal Transduct Target Ther 9 (2024): 332. 39592582 10.1038/s41392-024-02039-0 PMC11627502

[416] S. McCutcheon , D. Rohm , N. Iglesias , and C. Gersbach , “Epigenome Editing Technologies for Discovery and Medicine,” Nature Biotechnology 42 (2024): 1199–1217. 10.1038/s41587-024-02320-1 39075148

[417] X. Yu , H. Zhao , R. Wang , et al., “Cancer Epigenetics: From Laboratory Studies and Clinical Trials to Precision Medicine,” Cell Death Discov 10 (2024): 28. 38225241 10.1038/s41420-024-01803-z PMC10789753

[418] X. Xu and L. Qi , “A CRISPR‐dCas Toolbox for Genetic Engineering and Synthetic Biology,” Journal of Molecular Biology 431 (2019): 34–47. 29958882 10.1016/j.jmb.2018.06.037

[419] P. Jogam , D. Sandhya , A. Alok , V. Peddaboina , V. Allini , and B. Zhang , “A Review on CRISPR/Cas‐based Epigenetic Regulation in Plants,” International Journal of Biological Macromolecules 219 (2022): 1261–1271. 36057300 10.1016/j.ijbiomac.2022.08.182

[420] S. Xu , J. Kim , Q. Tang , et al., “CAS9 is a Genome Mutator by Directly Disrupting DNA‐PK Dependent DNA Repair Pathway,” Protein Cell 11 (2020): 352–365. 32170574 10.1007/s13238-020-00699-6 PMC7196600

[421] F. Adikusuma , S. Piltz , M. Corbett , et al., “Large Deletions Induced by Cas9 Cleavage,” Nature 560 (2018): E8–E9. 30089922 10.1038/s41586-018-0380-z

[422] S. Ling , X. Zhang , Y. Dai , et al., “Customizable Virus‐Like Particles Deliver CRISPR‐Cas9 ribonucleoprotein for Effective Ocular Neovascular and Huntington's disease Gene Therapy,” Nature Nanotechnology 20 (2025): 543–553. 10.1038/s41565-024-01851-7 PMC12015117 39930103

[423] X. Bian , L. Zhou , Z. Luo , et al., “Emerging Delivery Systems for Enabling Precision Nucleic Acid Therapeutics,” ACS Nano 19 (2025): 4039–4083. 39834294 10.1021/acsnano.4c11858

[424] Y. Zuo , C. Zhang , Y. Zhou , et al., “Liver‐specific in Vivo Base Editing of Angptl3 via AAV Delivery Efficiently Lowers Blood Lipid Levels in Mice,” Cell Biosci 13 (2023): 109. 37322547 10.1186/s13578-023-01036-0 PMC10273718

[425] C. Aging Biomarker , H. Bao , J. Cao , et al., “Biomarkers of Aging,” Sci China Life Sci 66 (2023): 893–1066. 37076725 10.1007/s11427-023-2305-0 PMC10115486

[426] A. Kane and D. Sinclair , “Epigenetic Changes During Aging and Their Reprogramming Potential,” Critical Reviews in Biochemistry and Molecular Biology 54 (2019): 61–83. 30822165 10.1080/10409238.2019.1570075 PMC6424622

[427] S. Liesenfelder , M. Elsafi Mabrouk , J. Iliescu , et al., “Epigenetic Editing at Individual Age‐associated CpGs Affects the Genome‐wide Epigenetic Aging Landscape,” Nat Aging 5, no. 6 (2025): 997–1009, 10.1038/s43587-025-00841-1. 40128456 PMC12176646

[428] M. Ehrlich , “DNA Hypermethylation in Disease: Mechanisms and Clinical Relevance,” Epigenetics 14 (2019): 1141–1163. 31284823 10.1080/15592294.2019.1638701 PMC6791695

[429] S. Yi and K. Kim , “New Insights Into the Role of Histone Changes in Aging,” International Journal of Molecular Sciences 21 (2020): 8241. 33153221 10.3390/ijms21218241 PMC7662996

[430] F. Rossiello , D. Jurk , J. Passos , and F. d'Adda di Fagagna , “Telomere Dysfunction in Ageing and Age‐related Diseases,” Nature Cell Biology 24 (2022): 135–147. 35165420 10.1038/s41556-022-00842-x PMC8985209

[431] J. Amorim , G. Coppotelli , A. Rolo , C. Palmeira , J. Ross , and D. Sinclair , “Mitochondrial and Metabolic Dysfunction in Ageing and Age‐related Diseases,” Nature reviews Endocrinology 18 (2022): 243–258. 10.1038/s41574-021-00626-7 PMC9059418 35145250

[432] D. Cheishvili , L. Boureau , and M. Szyf , “DNA Demethylation and Invasive Cancer: Implications for Therapeutics,” British Journal of Pharmacology 172 (2015): 2705–2715. 25134627 10.1111/bph.12885 PMC4439869

[433] A. Ocampo , P. Reddy , and J. Belmonte , “Anti‐Aging Strategies Based on Cellular Reprogramming,” Trends in Molecular Medicine 22 (2016): 725–738. 27426043 10.1016/j.molmed.2016.06.005

[434] S. Velychko , K. Adachi , K. Kim , et al., “Excluding Oct4 From Yamanaka Cocktail Unleashes the Developmental Potential of iPSCs,” Cell Stem Cell 25 (2019): 737–753. e734. 31708402 10.1016/j.stem.2019.10.002 PMC6900749

[435] J. Wu , T. Shao , Z. Tang , et al., “Highly Efficient Construction of Monkey Blastoid Capsules From Aged Somatic Cells,” Nature Communications 16 (2025): 1130. 10.1038/s41467-025-56447-z PMC11775175 39875393

[436] W. Dong , S. Liu , S. Li , and Z. Wang , “Cell Reprogramming Therapy for Parkinson's Disease,” Neural Regen Res 19 (2024): 2444–2455. 38526281 10.4103/1673-5374.390965 PMC11090434

[437] S. Wen , R. Zheng , C. Cai , and W. Jiang , “Chemical‐based Epigenetic Reprogramming to Advance Pluripotency and Totipotency,” Nature Chemical Biology 21, no. 5 (2025): 635–647, 10.1038/s41589-025-01874-8. 40251434

[438] M. Habets , M. Rots , and L. Chiapperino , “Meeting Report on the Round Table Discussions ‘Epigenetics and Society’ CLEPIC24,” Epigenetics Communications 5 (2025), 10.1186/s43682-025-00035-1.

[439] T. Tollefsbol , “Advances in Epigenetic Technology,” Methods in Molecular Biology 791 (2011): 1–10. 21913067 10.1007/978-1-61779-316-5_1 PMC3227536

[440] N. Rivero‐Segura , O. Bello‐Chavolla , O. Barrera‐Vazquez , L. Gutierrez‐Robledo , and J. Gomez‐Verjan , “Promising Biomarkers of human Aging: In Search of a Multi‐omics Panel to Understand the Aging Process From a Multidimensional Perspective,” Ageing Research Reviews 64 (2020): 101164. 32977058 10.1016/j.arr.2020.101164

[441] F. Xiao , H. Wang , L. Zhao , et al., “Methylome Analysis in Long‐lived Men Deciphers DNA Methylation Modifications Associated With Male Longevity in Humans,” Cell reports 44 (2025): 115158. 39772390 10.1016/j.celrep.2024.115158

[442] W. Xie , Y. Ke , Q. You , et al., “Single‐Cell RNA Sequencing and Assay for Transposase‐Accessible Chromatin Using Sequencing Reveals Cellular and Molecular Dynamics of Aortic Aging in Mice,” Arteriosclerosis, Thrombosis, and Vascular Biology 42 (2022): 156–171. 34879708 10.1161/ATVBAHA.121.316883

[443] K. Miskimen , E. Chan , and J. Haines , “Assay for Transposase‐Accessible Chromatin Using Sequencing (ATAC‐seq) Data Analysis,” Current protocols in human genetics 92 (2017), 10.1002/cphg.32. 20 24 21‐20 24 13. 28075484

[444] I. Beerman and D. Rossi , “Epigenetic Control of Stem Cell Potential During Homeostasis, Aging, and Disease,” Cell Stem Cell 16 (2015): 613–625. 26046761 10.1016/j.stem.2015.05.009 PMC4469343

[445] Y. Gao , Y. Chi , Y. Chen , et al., “Multi‐omics Analysis of human Mesenchymal Stem Cells Shows Cell Aging That Alters Immunomodulatory Activity Through the Downregulation of PD‐L1,” Nature Communications 14 (2023): 4373. 10.1038/s41467-023-39958-5 PMC10359415 37474525

[446] J. Si , Y. Ma , C. Yu , et al., “DNA Methylation Age Mediates Effect of Metabolic Profile on Cardiovascular and General Aging,” Circulation Research 135 (2024): 954–966. 39308399 10.1161/CIRCRESAHA.124.325066

[447] Z. Zheng , J. Li , T. Liu , et al., “DNA Methylation Clocks for Estimating Biological Age in Chinese Cohorts,” Protein Cell 15 (2024): 575–593. 38482631 10.1093/procel/pwae011 PMC11259550

[448] J. Rutledge , H. Oh , and T. Wyss‐Coray , “Measuring Biological Age Using Omics Data,” Nature Reviews Genetics 23 (2022): 715–727. 10.1038/s41576-022-00511-7 PMC10048602 35715611

[449] H. Huang , Y. Chen , W. Xu , et al., “Decoding Aging Clocks: New Insights From Metabolomics,” Cell metabolism 37 (2025): 34–58. 39657675 10.1016/j.cmet.2024.11.007

[450] K. Ying , H. Liu , A. Tarkhov , et al., “Causality‐enriched Epigenetic Age Uncouples Damage and Adaptation,” Nat Aging 4 (2024): 231–246. 38243142 10.1038/s43587-023-00557-0 PMC11070280

[451] M. He , X. Zhou , Z. Li , et al., “Programmable Transcriptional Modulation With a Structured RNA‐Mediated CRISPR‐dCas9 Complex,” Journal of the American Chemical Society 144 (2022): 12690–12697. 35792375 10.1021/jacs.2c02271

[452] M. Cappelluti , V. Mollica Poeta , S. Valsoni , et al., “Durable and Efficient Gene Silencing in Vivo by Hit‐and‐run Epigenome Editing,” Nature 627 (2024): 416–423. 38418872 10.1038/s41586-024-07087-8 PMC10937395

[453] Y. Wang , R. Huang , S. Feng , and R. Mo , “Advances in Nanocarriers for Targeted Drug Delivery and Controlled Drug Release,” Chin J Nat Med 23 (2025): 513–528. 40383609 10.1016/S1875-5364(25)60861-2

[454] C. Policarpi , M. Munafo , S. Tsagkris , V. Carlini , and J. Hackett , “Systematic Epigenome Editing Captures the Context‐dependent Instructive Function of Chromatin Modifications,” Nature Genetics 56 (2024): 1168–1180. 38724747 10.1038/s41588-024-01706-w PMC11176084

[455] A. Majchrzak‐Celinska , A. Warych , and M. Szoszkiewicz , “Novel Approaches to Epigenetic Therapies: From Drug Combinations to Epigenetic Editing,” Genes (Basel) 12 (2021): 208. 33572577 10.3390/genes12020208 PMC7911730

[456] D. Burdusel , T. Doeppner , R. Surugiu , D. Hermann , D. Olaru , and A. Popa‐Wagner , “The Intersection of Epigenetics and Senolytics in Mechanisms of Aging and Therapeutic Approaches,” Biomolecules 15 (2024): 18. 39858413 10.3390/biom15010018 PMC11762397

[457] J. de Toro‐Martin , B. Arsenault , J. Despres , and M. Vohl , “Precision Nutrition: A Review of Personalized Nutritional Approaches for the Prevention and Management of Metabolic Syndrome,” Nutrients 9 (2017): 913. 28829397 10.3390/nu9080913 PMC5579706

[458] R. Nativio , Y. Lan , G. Donahue , et al., “An Integrated Multi‐omics Approach Identifies Epigenetic Alterations Associated With Alzheimer's Disease,” Nature Genetics 52 (2020): 1024–1035. 32989324 10.1038/s41588-020-0696-0 PMC8098004

[459] A. Herman , J. Occean , and P. Sen , “Epigenetic Dysregulation in Cardiovascular Aging and Disease,” J Cardiovasc Aging 1 (2021), doi:10.20517/jca.2021.16. PMC8594871 34790973

