# Correction to “Epigenetic Regulation of Aging and Its Rejuvenation”

**DOI:** 10.1002/mco2.70755

**Published:** 2026-08-05

Y. An, Q. Wang, K. Gao, et al., “Epigenetic Regulation of Aging and Its Rejuvenation,” *MedComm* 6, no. 9 (2025): e70369, https://doi.org/10.1002/mco2.70369.

In Table 1 (pages 21–22 of 35) of the above‐mentioned review article, the headers of the last two columns were inadvertently reversed. The column header labeled “Clinical implication” actually contains ClinicalTrials.gov ID data, while the column header labeled “Clinicaltrials.gov ID” contains clinical implication information. This misalignment may cause confusion for readers.

Correction

The headers of the last two columns in Table 1 should be swapped to ensure accurate correspondence between column titles and content. Specifically:

Rename the column currently labeled “Clinical implication” to “ClinicalTrials.gov ID”

Rename the column currently labeled “Clinicaltrials.gov ID” to “Clinical implication”

We apologize for this error.

